# *In Silico* Modeling of Itk Activation Kinetics in Thymocytes Suggests Competing Positive and Negative IP_4_ Mediated Feedbacks Increase Robustness

**DOI:** 10.1371/journal.pone.0073937

**Published:** 2013-09-16

**Authors:** Sayak Mukherjee, Stephanie Rigaud, Sang-Cheol Seok, Guo Fu, Agnieszka Prochenka, Michael Dworkin, Nicholas R. J. Gascoigne, Veronica J. Vieland, Karsten Sauer, Jayajit Das

**Affiliations:** 1 Battelle Center for Mathematical Medicine, The Research Institute at the Nationwide Children’s Hospital, Columbus, Ohio, United States of America; 2 Department of Pediatrics, The Ohio State University, Columbus, Ohio, United States of America; 3 Department of Physics, The Ohio State University, Columbus, Ohio, United States of America; 4 Department of Statistics, The Ohio State University, Columbus, Ohio, United States of America; 5 Department of Mathematics, The Ohio State University, Columbus, Ohio, United States of America; 6 Biophysics Graduate Program, The Ohio State University, Columbus, Ohio, United States of America; 7 Department of Immunology and Microbial Science, The Scripps Research Institute, La Jolla, California, United States of America; 8 Institute of Computer Science, Polish Academy of Sciences, Warsaw, Poland; University of Catania, Italy

## Abstract

The inositol-phosphate messenger inositol(1,3,4,5)tetrakisphosphate (IP_4_) is essential for thymocyte positive selection by regulating plasma-membrane association of the protein tyrosine kinase Itk downstream of the T cell receptor (TCR). IP_4_ can act as a soluble analog of the phosphoinositide 3-kinase (PI3K) membrane lipid product phosphatidylinositol(3,4,5)trisphosphate (PIP_3_). PIP_3_ recruits signaling proteins such as Itk to cellular membranes by binding to PH and other domains. In thymocytes, low-dose IP_4_ binding to the Itk PH domain surprisingly promoted and high-dose IP_4_ inhibited PIP_3_ binding of Itk PH domains. However, the mechanisms that underlie the regulation of membrane recruitment of Itk by IP_4_ and PIP_3_ remain unclear. The distinct Itk PH domain ability to oligomerize is consistent with a cooperative-allosteric mode of IP_4_ action. However, other possibilities cannot be ruled out due to difficulties in quantitatively measuring the interactions between Itk, IP_4_ and PIP_3_, and in generating non-oligomerizing Itk PH domain mutants. This has hindered a full mechanistic understanding of how IP_4_ controls Itk function. By combining experimentally measured kinetics of PLCγ1 phosphorylation by Itk with *in silico* modeling of multiple Itk signaling circuits and a maximum entropy (MaxEnt) based computational approach, we show that those *in silico* models which are most robust against variations of protein and lipid expression levels and kinetic rates at the single cell level share a cooperative-allosteric mode of Itk regulation by IP_4_ involving oligomeric Itk PH domains at the plasma membrane. This identifies MaxEnt as an excellent tool for quantifying robustness for complex TCR signaling circuits and provides testable predictions to further elucidate a controversial mechanism of PIP_3_ signaling.

## Introduction

Hydrolysis of plasma membrane phospholipids generates various cellular messengers [1]. Among these, multiple isomeric inositol phosphates (IP) [1]–[4] form an “IP code” [5] whose members can regulate critical decision processes downstream of many receptors in diverse cell types. However, the specific mechanisms and precise molecular circuitries that underlie the regulation of cell functions by soluble IPs are poorly understood. We and others previously reported an essential role for inositol(1,3,4,5) tetrakisphosphate (IP_4_) in regulating T cell development [2], [3], [6], [7].

T cells are key mediators of adaptive immune responses. Through a plasma-membrane anchored TCR, they recognize pathogen-derived peptides bound to Major Histocompatibility Complex proteins (pMHC) on the surface of antigen-presenting cells. TCR engagement triggers activation, proliferation and effector functions in peripheral T cells that then kill pathogen-infected cells and control immune responses. During T cell development in the thymus, somatic mutation of the antigen-binding TCR α/β subunit genes creates a thymocyte repertoire with random TCR specificities. However, many of these TCRs are non-functional or interact with the body’s self-antigens with high affinity, causing autoimmune disorders if the respective T cells were allowed to mature. To prevent this, thymic selection processes eliminate thymocytes carrying TCRs that fail to interact with, or interact with too strong affinity with self-peptide-MHC (pMHC) complexes. The latter process is known as negative selection, a key mechanism of central tolerance. Only those thymocytes whose TCR generates mild signals are positively selected to mature into T cells, which then populate peripheral organs. Balanced positive and negative selections are critical for generating a diverse but self-tolerant T cell repertoire [8]–[10]. Recent experiments provided a more complex picture of thymic selection, where certain high affinity peptides can ‘agonist select’ distinct regulatory T cell types [11], [12].

TCR-pMHC binding triggers a series of signaling reactions, resulting in the formation of a plasma membrane-proximal signalosome containing Src (Lck, Fyn) and Syk family protein tyrosine kinases (Zap70), cytosolic (such as SLP-76, Gads, Grb-2), and transmembrane adapter proteins (such as LAT). TCR-activation of phosphoinositide 3-kinase (PI3K) converts the abundant membrane phospholipid phosphatidylinositol(4,5) bisphosphate (PIP_2_) into phosphatidylinositol(3,4,5) trisphosphate (PIP_3_). By binding to pleckstrin homology (PH) or other protein-domains, PIP_3_ recruits key effectors such as the Tec family protein tyrosine kinase Itk (IL-2 inducing T cell activation kinase). Itk also contains SH2 and SH3 domains that bind to signalosome components. The Src kinase Lck phosphorylates Y_511_ in the A-loop of the murine (Y_512_ in the human) Itk kinase domain [13]. Subsequently, Itk propagates TCR signals by phosphorylating and activating signalosome-recruited phospholipase Cγ1 (PLCγ1). PLCγ1 then hydrolyzes PIP_2_ into the second messenger molecules diacylglycerol (DAG) and inositol(1,4,5) trisphosphate (IP_3_). The membrane lipid DAG further recruits and activates Rasgrp1 and PKCs that in turn activate the GTPase Ras and the Bcl-10/CARMA1/MALT complex, ultimately triggering thymocyte positive and negative selection, or peripheral T cell responses [14], [15]. Soluble IP_3_ mobilizes Ca^2+^ from the endoplasmic reticulum (ER). Moreover, IP_3_ 3-kinases such as ItpkB can phosphorylate IP_3_ at its 3-position into IP_4_ [2], [6], [7], [14], [16]. IP_4_ chemically resembles the PH domain binding PIP_3_ tetraphosphoinositol headgroup [14], [17].

We and others identified ItpkB as essential for thymocyte positive selection [2], [6], [7]. *ItpkB^−/−^* DP thymocytes show intact proximal TCR signaling but defective IP_4_ production, Itk PIP_3_-binding, signalosome recruitment and activation with ensuing reduced PLCγ1 activation, DAG production, and, Ras/Erk activation [2]. The ability of soluble IP_4_ to bind to the Itk PH domain and in low µM doses promote PIP_3_ binding, and the ability of the Itk PH domain to oligomerize suggested that IP_4_ might promote Itk recruitment to membrane-PIP_3_ through a cooperative-allosteric mechanism. In this model, IP_4_-binding to one PH domain in an oligomer allosterically increases the ligand affinities of the other PH domains in the same oligomer [2]. IP_4_ promoted Itk activation appears to be required for sufficient Itk activation to ensure positive selection, because an exogenous DAG-analog restored positive selection of *ItpkB^−/−^* thymocytes [2]. However, high-dose IP_4_ inhibited Itk PH domain binding to PIP_3_ *in vitro* [2]. Whether it does so *in vivo* is unknown [14]. In neutrophils, NK cells and myeloid progenitors, IP_4_ competitively limits Akt PH domain binding to membrane PIP_3_ [18]–[20]. Which PH domains are positively versus negatively controlled by IP_4_, and what determines whether IP_4_ promotes or inhibits PH domain binding to PIP_3_ or leaves it unaffected are important open questions [14], [21]. In particular, the Itk PH domain might be bi-modally regulated by IP_4_. However, the detailed molecular interactions between Itk, PIP_3_ and IP_4_ *in vivo* are not well characterized. This leaves room for multiple alternate hypotheses/mechanisms. For example, one could also propose that the binding affinity of PIP_3_ and IP_4_ for Itk changes from a low to a fixed high value above a threshold IP_4_ concentration. Such a mechanism implies that the interaction of Itk with IP_4_ and PIP_3_ after the threshold IP_4_ concentration is reached does not involve a positive feedback. The situation is further confounded by elusive results from experiments probing Itk oligomerization [2], [22]–[28].

The current lack of a mechanistic understanding of how IP_4_ controls Itk PIP_3_-interactions and whether Itk PH domain oligomerization is physiologically relevant arises from difficulties in quantitatively measuring the interactions between Itk, IP_4_ and PIP_3_, and in generating soluble Itk PH domain preparations for biophysical studies and non-oligomerizing Itk PH domain mutants for genetic analyses. Additional limitations arise from difficulties in measuring membrane recruitment of Itk in cell population based assays. It is also difficult to measure PIP_3_ bound Itk or phosphorylation of PLCγ1, a substrate of PIP_3_ bound Itk, in large numbers of individual cells using flow cytometry techniques due to limited antibody quality. *In vitro* and cell-based studies based on ectopic Itk expression suggest the existence of several different monomeric and oligomeric Itk species, including head-to-head and head-to-tail dimers [2], [22]–[28]. Andreotti and colleagues [22] showed that Itk molecules can self associate via their SH2–SH3 domains into auto-inhibitory oligomers. This is hindered by SLP-76 interactions with the Itk SH2–SH3 domains. It was suggested that Itk molecules might exist as auto-inhibited multimers in the cytosol, but after plasma membrane recruitment, Itk monomers might mediate downstream activation [22], [26]. Other experiments [27], [28] employing fluorescence complementation showed that formation of Itk head-to-head and head-to-tail dimers requires the PH domain and may primarily occur at the plasma membrane, although low-abundance cytoplasmic dimers have not been excluded. Here, monomeric Itk was proposed to be primarily cytoplasmic and autoinhibited [27]. At least head-to-head dimerization is unaffected by mutations in the other (SH2/SH3) domains [28]. We found that the Itk PH domain can oligomerize with other Itk PH domains or full length Itk [2]. Thus, the PH domain is well suited to contribute to at least certain modes of Itk oligomerization, some of which could have positive or a combination of positive and negative functions regulated by IP_4_/PIP_3_. This could account for the limited activity-enhancing effect of disrupting SH3/SH2-domain mediated Itk dimerization [26].

Altogether, whether Itk PH domain dimerization has a physiological function, whether it promotes or inhibits Itk activation, whether IP_4_ controls Itk function through positive or negative feedback, or both, and whether IP_4_ has additional unrelated functions in thymocytes, are all contentious questions in the field. Resolving them is very important, because PI3K is a paramount regulator of signaling from many receptors in most cells. PIP_3_ hyperactivity is a major contributor to immune, metabolic and other diseases including cancers [29], [30]. IP_3_ 3-kinases are broadly expressed and IP_4_ has been found in many cell types. Thus, IP_4_ regulation of PIP_3_ function could be broadly important and elucidating the precise molecular mechanisms through which IP_4_ controls PIP_3_ signaling improves our understanding of a very fundamental and important signaling pathway with great therapeutic relevance [14].

To further explore how the presence or absence of Itk PH domain oligomerization, of positive or negative IP_4_ feedback or both, or of specific molecular modes of association of Itk, PIP_3_ and IP_4_ impact TCR signaling, we constructed seven different molecular models (Table 1 and Figure S1B). We used a Maximum Entropy (MaxEnt) [31]–[33] based approach to quantify the robustness of each model against variations in rate constants and protein expression levels at the single cell level. Each model was constrained to reproduce the Itk activation kinetics of an entire cell population measured in biochemical experiments. We found that those models involving dimeric Itk molecules with IP_4_ mediated competing positive and negative feedbacks are most robust. As in many other cell signaling systems [34], the actual signaling kinetics in thymocytes are likely to be robust against such variations, while retaining their sensitivity to small variations in antigen affinity or dose. On this basis, our simulations best support biphasic Itk regulation by IP_4_ in thymocytes. Future testing of this exciting hypothesis will require the so far unsuccessful generation of non-oligomerizing Itk PH domain mutants and their expression in *Itk^−/−^* mice, along with currently impossible single-cell measurements of IP_4_ levels in large cell populations.

**Table 1:** Molecular models describing interactions between Itk, IP_4_ and PIP_3_.

	M1	M2	M3	M7	M4	M5	M6
	Contain IP_4_ induced +ve feedback					No +ve feedback	
	Contain Itk dimers				Contains Itk monomers	Contains Itk dimers	Contains Itk monomers
**Effect of IP_4_ binding to one PH domain of an Itk dimer**	Increases affinity of the other PH domain toward IP_4_ and PIP_3_.	Same as in M1	Same as in M1.	Increases affinity of the other PH domain toward PIP_3_ and IP_4_.	IP_4_ and PIP_3_ bind to the Itk PH domain with weak affinities. However, IP_4_ bound to Itk gets replaced by PIP_3_ with high affinity, and then the PIP_3_ bound to Itk can get replaced by IP_4_ with high affinity.	No change in affinity	The monomeric PH domain binds IP_4_ and PIP_3_ with equal but always low affinity.
**Effect of PIP_3_ binding to one PH domain of an Itk dimer**	Increases affinity of the other PH domain for IP_4_ *and* PIP_3_.	*Does not* increase the affinity of the other PH domain for IP_4_ or PIP_3_.	Increases affinity of the other PH domain only for IP_4_ but *not* for PIP_3_.	Increases affinity of the other PH domain for PIP_3_ but *not* for IP_4_.		No change in affinity	
**Number of parameters (Rate constants+initial concentrations)**	5+3	5+3	5+3	5+3	4+3	3+3	3+3

## Results

### Multiple Molecular Models can be Constructed to Probe Itk, IP_4_, and PIP_3_ Interactions *in silico*

**Figure 1:**
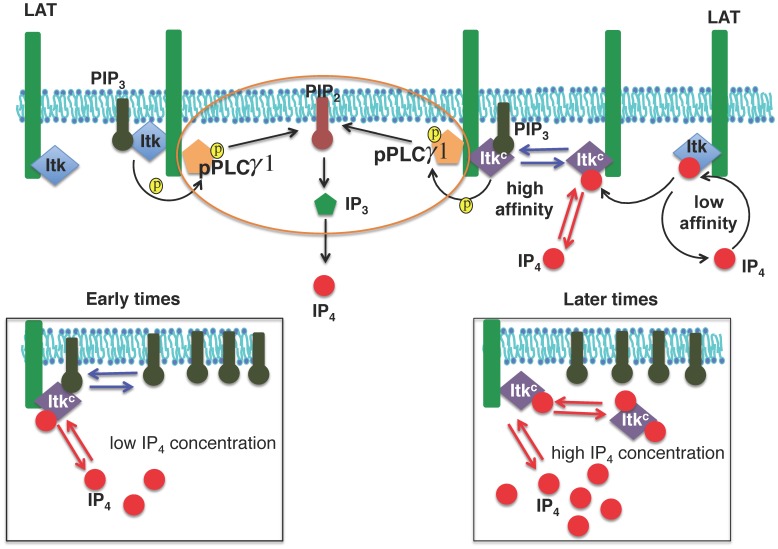
Relevant basic interactions between Itk, PIP_3_ and IP_4_. Following TCR-pMHC binding, Itk molecules are bound by the LAT signalosome via SLP-76 (not shown). Itk molecules (monomers or dimers, blue diamonds), bind the membrane lipid PIP_3_ with low affinity through their PH domains. PIP_3_ bound Itk phosphorylates and thereby activates LAT-bound PLCγ1. Activated PLCγ1 then hydrolyzes the membrane lipid PIP_2_ into the soluble second messenger IP_3_, a key mediator of Ca^2+^ mobilization. IP_3_ 3-kinase B (ItpkB) converts IP_3_ into IP_4_ (red filled circle). For our *in silico* models, we simplified this series of reactions, encircled by the orange oval, into a single second order reaction where PIP_3_ bound Itk converts PIP_2_ into IP_4_. In models M1–M4 and M7, IP_4_ modifies the Itk PH domain (denoted as Itk^C^, purple diamonds) to promote PIP_3_ and IP_4_ binding to the Itk PH domain. At the onset of the signaling, when the concentration of IP_4_ is smaller than that of PIP_3_, IP_4_ helps Itk^C^ to bind to PIP_3_ (left lower panel). However, as the concentration of IP_4_ is increased at later times, IP_4_ outcompetes PIP_3_ for binding to Itk^C^ and sequesters Itk^C^ to the cytosol (right lower panel). In models M5/M6, IP_4_ and PIP_3_ do not augment each other’s binding to Itk. However, IP_4_ still outcompetes PIP_3_ for Itk PH domain binding when the number of IP_4_ molecules becomes much larger than that of PIP_3_ molecules at later times.

We constructed seven different molecular models (Table 1, Figure S1B) based on available details about interactions between Itk, PIP_3_ and IP_4_ from the biochemical studies described above. Including Itk kinase domain activation by Lck only caused qualitative changes in the relative robustness of the models (Fig. S17, Tables S9–S15). Therefore, for simplicity, we considered models that do not contain Itk activation by Lck explicitly. We also did not consider Itk autophosphorylation explicitly in the models as it does not affect Itk catalytic activity. In addition, the role of Itk autophosphorylation in PLCγ1 activation remains unclear [22]. Since we aimed to elucidate general characteristics of the kinetics of PIP_3_ binding to Itk, we used a simplified modeling scheme (Fig. 1) and did not consider the detailed molecular composition of the TCR and the LAT associated signalsome. The models also do not investigate different mechanisms for formation of Itk oligomers. Rather, they probe the functional consequences of having Itk PH domain dimers versus monomers and how these can affect interactions between Itk, PIP_3_ and IP_4_ in the presence or absence of IP_4_ mediated positive feedback. The kinetics of PIP_3_ production due to signal-dependent recruitment of PI3K are not considered explicitly as PIP_3_ is produced at a much faster time scale (in seconds, [35] [36] [37]) than the time scales of PLCγ1 activation (up to 60 min, Figure S18). The concentrations of LAT bound Itk and of PIP_3_ were considered approximate markers for the strength of the stimulation generated by antigen-TCR interactions. Therefore, we considered fixed initial concentrations of Itk and PIP_3_ in the models. We approximated the production of IP_4_ from PIP_2_ by a single one-step reaction to simplify the models further.

The models can be broadly classified into two types: (i) Models M1–M4 and M7 containing IP_4_ mediated positive feedbacks. (ii) Models M5 and M6 lacking IP_4_ positive feedback. In each type, we further considered models that contained Itk dimers (models M1–M3, M5, M7), or monomers (models M4, M6). In models M1–M3, each of the two PH domains in the Itk dimer can independently bind to either IP_4_ or PIP_3_ with a weak affinity when the other PH domain is unoccupied. However, once a PH domain is bound to an IP_4_ molecule, it allosterically increases the affinity of the other PH domain for PIP_3_ and IP_4_. Models M1–M3 differ from each other in the relative increase in the affinities of one PH domain in the Itk dimer toward IP_4_ vs. PIP_3_ caused by IP_4_ or PIP_3_ binding to the other PH domain in the dimer. In contrast, in M7, binding of PIP_3_ to one PH domain in a dimer increases the affinity of the other PH domain for PIP_3_ but not for IP_4_. These models probed potential secondary interactions between Itk dimers and the membrane lipids. In the monomeric model, M4, IP_4_ binds the single Itk PH domain with a weak affinity and induces a conformational change that increases the affinity of this PH domain for both PIP_3_ and IP_4_. Models M5 and M6 lack positive IP_4_ feedback. Instead, the Itk PH domain binds to IP_4_ and PIP_3_ with equal affinity. These models probed a mechanism where the Itk PH domain interacts with IP_4_ and PIP_3_ once a small threshold IP_4_ concentration is generated. We assumed that the small threshold level of IP_4_ is generated at a time scale much smaller than the timescale (min) of robust Itk activation and did not consider the kinetics generating the threshold level of IP_4_ explicitly in M5 and M6. The models are summarized in Table 1, Figure S1B, and Tables S1–S8.

### The Shape of Transient Itk Activation Kinetics Depends on Specific Molecular Wirings and Feedbacks in the Different Models

**Figure 2:**
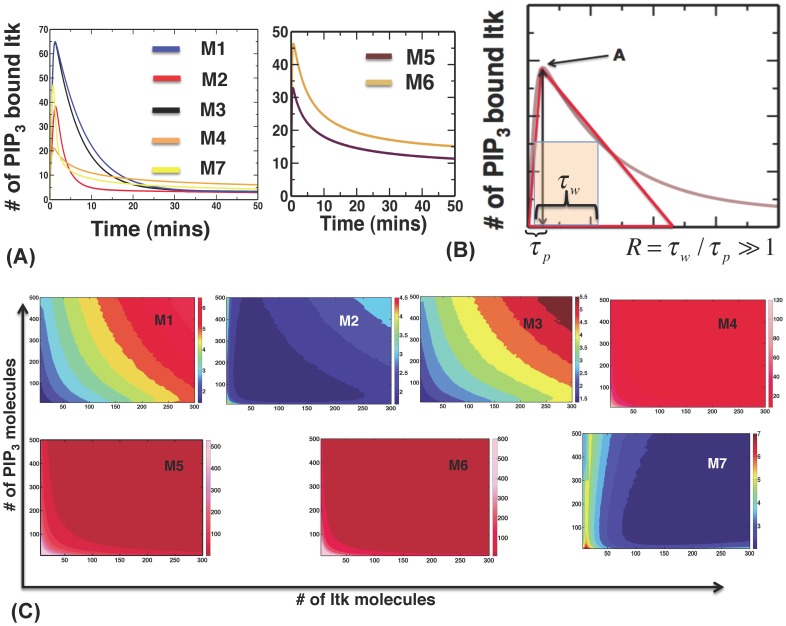
Different molecular interactions in models M1–M7 produce different temporal profiles of PIP_3_ binding to Itk. (**A**) Kinetics of PIP_3_ association of Itk for fixed initial PIP_3_ and Itk concentrations (100 and 370 molecules, respectively) in models with feedbacks (M1–M4, and M7, left panel) and no feedbacks (M5–M6, right panel). (B) The shapes of the temporal profiles can be characterized by the parameters peak time (*τ*_p_), peak width (*τ*_w_), and peak value or amplitude (*A*). The dimensionless asymmetry ratio *R* = *τ*_w_/*τ*_p_ quantifies how symmetric the shape of the time profile is. A larger R value indicates larger asymmetry. (C) Variations in R in models M1–M7 for different initial concentrations of Itk and PIP_3_. Color scales for R values are shown on the right of each panel.

We studied the kinetics of Itk binding to PIP_3_ using deterministic mass-action kinetic rate equations described by ordinary differential equations (ODE) for all the models, ignoring stochastic fluctuations in the copy numbers of signaling proteins occurring due to the intrinsic random nature of biochemical reactions [38]. Including such fluctuations did not change the kinetics qualitatively (Figures S2–S3). In all seven models, the kinetics of PIP_3_ bound Itk showed a transient behavior (Fig. 2A); PIP_3_ bound Itk started with a low concentration, reached a peak value at an intermediate time, and then fell back to a small concentration at later times. We found that initially few Itk molecules were bound to PIP_3_. With increasing time, more Itk molecules became associated with PIP_3_ molecules due to the binding reactions between Itk and PIP_3_. This produced the rise in the Itk-PIP_3_ concentration. However, as the concentrations of PIP_3_ bound Itk molecules increased, they also induced increased production of IP_4_ molecules. IP_4_ competed with PIP_3_ for binding to the Itk PH domain, and when the number of IP_4_ molecules exceeded that of PIP_3_ molecules, most of the Itk molecules were sequestered to the cytosol by forming stable complexes only with IP_4_. This reduced the rate of PIP_3_ association of Itk and eventually resulted in the decrease of the PIP_3_ bound Itk molecules. IP_4_ outnumbered PIP_3_ at later times because the number of PIP_2_ molecules, the source of IP_3_ and IP_4_ in a cell, is considered not limiting in contrast to PIP_3_ [37], [39]. We emphasize that the results of our models do not depend on the cytosolic nature of Itk-IP_4_ complexes, but on the model assumption that Itk (or Itk oligomers) bound to IP_4_ at every PH domain does not induce any PLCγ1 activation.

We characterized the ‘shape’ of the temporal profile of PIP_3_ bound Itk in terms of (i) the largest concentration of PIP_3_ bound Itk in the entire temporal profile (peak amplitude value *A*); (ii) the time taken for PIP_3_ bound Itk to reach the peak value (peak time *τ*_p_); and (iii) the time interval during which the PIP_3_ bound Itk concentration is greater than or equal to half of the peak value (peak duration *τ*_w_, Fig. 2B). A dimensionless variable quantifying the asymmetry in the shape of the kinetics, denoted as the asymmetry ratio R* = τ_w_/τ_p_* (Fig. 2B), turned out to be a useful indicator for differentiating temporal profiles of concentrations of PIP_3_ bound Itk in simulations and experiments. R also quantifies if the time scale for the decay of the concentration of PIP_3_ bound Itk after the peak value is reached is larger than or comparable to *τ*_p_ (the timescale for producing the peak value *A*). E.g.,when *R* ≅ 1, it implies that the *τ*_p_ is comparable to decay time. *R* > 1 indicates a more persistent signal with long decay times. Differences (transient vs. persistent) in the shapes of kinetic profiles of signals downstream of Itk activation have been observed to influence thymocyte decision outcomes [2], [40]. Therefore, R, which characterizes the persistent or transient nature of Itk activation, also contains details directly relevant for thymic selection outcomes. We found that the shape of the transient kinetics of PIP_3_ binding to Itk varied substantially depending on the feedbacks and the molecular wiring of the networks. Since the reaction rates used in the models are difficult to measure *in vivo* for thymocytes, we estimated the rates based on interaction strengths measured *in vitro* between PH domains and inositol phosphates in other cells, and from temporal profiles of PLCγ1 activation measured in experiments with T cells (Tables S1–S7). Previous work demonstrated the essential role of phosphorylated PLCγ1 and its kinetics in regulating thymocyte positive, negative and agonist selection [41], [42]. Phospho-PLCγ1 is also known to mirror other indicators of T cell activation such as TCRζ- or LAT-phosphorylation, or Erk-activation [2], [40]. Therefore phospho-PLCγ1 is a relevant marker for functional T cell responses.

We studied variations in the kinetics of PIP_3_ bound Itk for different initial concentrations of Itk and PIP_3_. This probed how different ligand doses or affinities affected the PIP_3_ binding of Itk. We found that the peak concentration of PIP_3_ bound Itk increased in a graded manner with increasing initial Itk and PIP_3_ concentrations in all models (Figure S4). However, the peak time *τ*_p_ (Figure S5), and the asymmetry ratio R (Fig. 2C), were affected differently in different models. Among the feedback models, M1–M3 and M7 containing Itk dimers generated smaller values (varied between 2 to 6) of R compared to monomeric model M4 which produced a much larger range of R (∼20 -120) (Fig. 2C). The models lacking positive feedbacks (M5 and M6) generated large values (∼100–700) of *R* compared to feedback models with Itk dimers (Fig. 2C). In the feedback models, the initial low affinity binding-unbinding interactions between Itk and PIP_3_/IP_4_ are converted into high affinity interactions due to the positive feedback. Therefore, a large part of *τ*_p_ is spent in building up the positive feedback interactions controlled primarily by the weak affinity binding-unbinding rates (or K_D_). The small values of R in models M1–M3 and M7 occurred because stronger negative feedbacks resulted in much smaller timescales for substantially reducing the concentration of PIP_3_ bound Itk after it reached its peak value compared to the other models. In the models lacking positive feedback (M5, M6), concentrations of PIP_3_ bound Itk decreased at a much slower rate than the peak time leading to large values of R. In the monomeric model, the relatively weaker strength of positive and negative feedbacks resulted in larger decay time scales for the PIP_3_ bound Itk, producing large values of R. These results are analyzed in detail in the web supplement and Figures S6–S11. We will show below how the ability of feedback models with Itk dimers to produce R values within a small range leads to higher robustness of these models against parameter variations at the single cell level.

### Models Containing Dimeric Itk and IP_4_ Mediated Dueling Positive and Negative Feedbacks are the Most Robust Models

#### Quantification of robustness in *in silico* models

The reaction rates describing non-covalent primary and secondary interactions between Itk, PIP_3_ and IP_4_ can depend on specific properties of the local cellular environment, such as local membrane curvature [43], molecular crowding [44], [45], and the presence of different lipid molecules in the proximity [46]. Since these factors can vary from cell to cell, the reaction rates can vary at the single cell level. In addition, protein expression levels can vary between cells. Such variations are also known as extrinsic noise fluctuations [47], [48]. The IP_4_ production rate depends on the concentrations of ItpkB, Calmodulin (CaM), and released calcium [3]. Hence, the IP_4_ production rates in our models which approximate all such dependencies with a one-step reaction will vary between individual cells as well. The above variations are capable of producing differences in the shapes of temporal profiles of activation of signaling proteins in individual cells [49]. In the coarse-grained or approximate models we have constructed, many molecular details have been approximated. For example, multiple phosphorylation sites or SH2/SH3 binding sites of Itk, LAT, SLP-76 and their regulation via TCR induced signaling are not considered explicitly [3], [22], [50], [51]. These detailed molecular signaling events can depend on the concentrations of proteins, enzymes, and lipids, and can thus be regulated differently in different cells due to extrinsic noise fluctuations. Consequently, the rates in our *in silico* models that effectively describe those detailed signaling events can vary from cell to cell. Consistent with this view, our simulations with the ODE models showed that the shape of the kinetics of PIP_3_ bound Itk, characterized by, *A*, *τ*_p_, and R, changed significantly as the rate constants and initial concentrations in a model were varied (Figures S12–S14). Thus, activation kinetics of a marker molecule (e.g. PLCγ1) measured in experiments (e.g., immunoblots) assaying a large cell population represent averages over a range of temporal profiles with different shapes occurring at the single cell level.

We found that for some ranges of the reaction rates, multiple different *in silico* models can produce the same values of *A*, *τ*_p_, and R (Figures S12–S14). This implied that more than one *in silico* model *could* reproduce the mean temporal profile measured in cell population assays. However, it is possible that each model could show a different degree of robustness to variations in reaction rates and initial concentrations at the single cell level. Robustness of time dependent responses in a cell population against variations at the single cell levels has been observed in several systems, e.g., oscillations in adenosine 3′,5′ cyclic monophosphate (cAMP) concentrations in a population of *Dyctostelium* [52], [53], or damped oscillations of protein 53 (p53) in a population of human breast cancer epithelial cells [54]. Robustness of cellular functions against variations in external conditions and cell-to-cell variability has been proposed as a required design principle for a wide range of biochemical networks [55]–[58]. We therefore decided to ask: Which model(s) can accommodate the largest variation in reaction rates and initial concentrations, while reproducing the mean temporal profile of PIP_3_ bound Itk measured as generation of phosphorylated PLCγ1 in cell population experiments? We postulate that the answer to this question will point us to the molecular circuitry most likely to be the relevant model, in the sense that it robustly produces a specific temporal response at the cell population level despite variations in the kinetics in individual cells.

**Figure 3:**
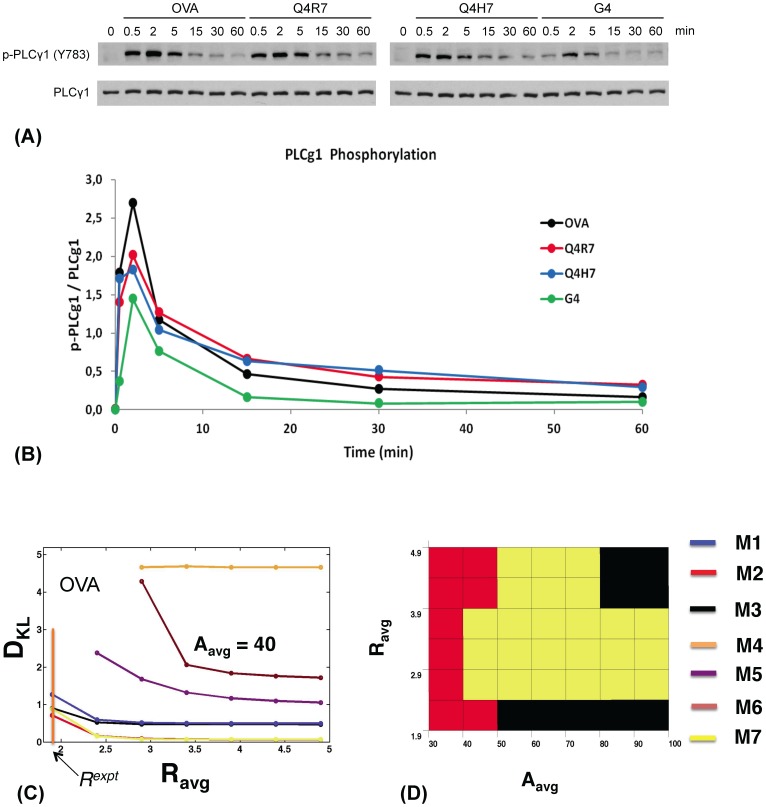
Experimentally measured PLCγ1activation kinetics in DP thymocytes stimulated with TCR ligands of different affinities and robustness of *in silico* models. (A) Immunoblots showing Y_783_-phosphorylated (upper panels) and total (lower panels) PLCγ1 protein amounts in *RAG2^−/−^MHC^−/−^ OT1 TCR-transgenic* DP thymocytes stimulated for the indicated times with MHCI tetramers presenting the indicated altered peptide ligands (APL). (B) Phospho-PLCγ1 levels normalized to total PLCγ1 protein amounts plotted over time for the indicated APLs. Their TCR affinity decreases in the order OVA (black)>Q4R7 (red)>Q4H7 (blue)>G4 (green). Band intensities were quantified via scanning and analysis with *ImageJ* software. Representative of several independent experiments. (C) Variation of the Kulback-Leibler distance D_KL_ with *R* for models M1–M3 (blue, red and black, respectively), M7 (yellow), and M4–M6 (orange, purple, and maroon, respectively) at high initial Itk (Itk^0^ = 140 molecules) and PIP_3_ concentrations (PIP_3_^0^ = 530 molecules), representing high-affinity OVA stimulation for *τ*_p_ = 2 min and *A* (shown as *A*_avg_) = 40 molecules. Note we use *A* to represent the amplitude *A* ^expt^ in experiments measuring fold change in Itk phosphorylation (see the main text for further details). The vertical orange bar indicates R*^expt^* for OVA. Color legend in (D). (D) The color map shows which model is most robust (has the lowest D_KL_) as *R^expt^* and *A* (shown as *A*_avg_) are varied for the same parameters as in (C). The color legend is depicted on the right.

To identify the most robust model(s), we quantified robustness using a method based on the principle of Maximum Entropy (MaxEnt) [31]–[33]. MaxEnt provides a mechanism for estimating the probability distribution of the rate constants and initial Itk and PIP_3_ concentrations under constraints derived from experimental data (Fig. 3A–B, 4A–B). Here, we used the experimentally obtained values *τ_p_^expt^* and *R^expt^* as the constraints. It is difficult to directly relate the amplitude (in units of number of molecules in the simulation box) in the *in silico* models to experiments, where amplitudes are calculated from the fold change of the immunoblot intensities upon stimulation. Therefore, the experimental values of *A* can be related to the number of activated molecules, at best, through a proportionality constant dependent on specific protocols used in an assay. Because of these issues we chose a value of *A^expt^*, representing *A* in experiments, where every *in silico* model produced amplitudes at *A^expt^* for a set of parameters within the range of variations considered here. We then varied *A^expt^* to investigate the change in robustness of the models and address the arbitrariness in the choice of *A^expt^*. We constructed a relative entropy measure (Kullback-Leibler distance, D_KL_, calculated on the log_10_ scale) [59] that measures the deviation of the constrained MaxEnt distribution from the unconstrained MaxEnt distribution, in which all values of the rate constants and initial concentrations are equally likely (uniform distribution). Thus D_KL_ is being used as a measure of how “close” each model can get to one which is completely indifferent to the values of the rate constants, given the experimental constraints. We then compared D_KL_ across our models in order to find the most robust model compatible with experimental results. Note that the minimum value of D_KL_ is 0, with smaller values indicating greater robustness. We have also analyzed D_KL_ for different models when *τ_p_^expt^* and *R^expt^* were constrained but the amplitude *A^expt^* was not constrained (Figure S23). The results are qualitatively similar to that of the case when *τ_p_^expt^*, *R^expt^*, and *A^expt^* were constrained. This indicates that the robustness of the temporal shape of Itk membrane recruitment kinetics rather than the amplitude contributes substantially toward the increased robustness of the feedback models with Itk dimers.

**Figure 4:**
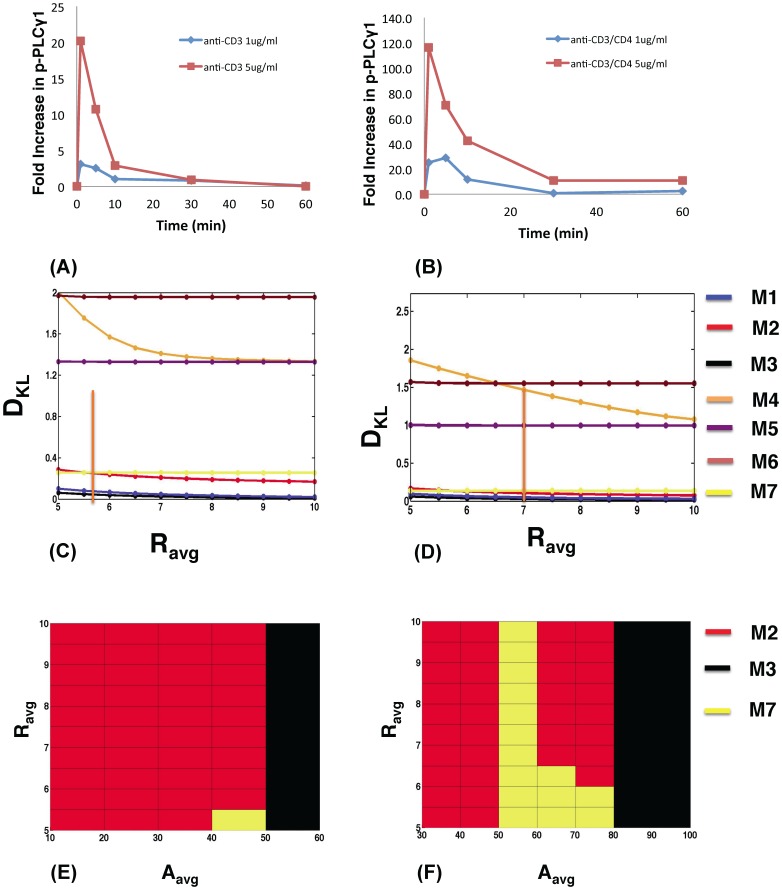
Models containing Itk dimers and dueling feedbacks also show higher robustness for polyclonal T cells stimulated by anti-CD3 antibodies. PLCγ1 phosphorylation kinetics in *MHC^−/−^* T cells stimulated by antibodies against (A) CD3 or (B) CD3 and CD4 at 1 µg/ml versus 5 µg/ml. (C) Variation of D_KL_ with R for the *in silico models* M1–M3 (blue, red and black, respectively), M7 (yellow), and M5–M6 (purple and maroon, respectively) at initial Itk (Itk^0^ = 100 molecules) and PIP_3_ concentrations (PIP_3_^0^ = 370 molecules) at *τ*_p_ = 1 min and *A*_avg_ = 60 molecules, representing anti-CD3 stimulation at 5 µg/ml. The orange bar indicates R*^expt^*. Note we use *A*_avg_ to represent the amplitude A^expt^ in experiments measuring fold change in Itk phosphorylation (see the main text for further details). (D) Variation of D_KL_ with R for anti-CD3/CD4 stimulation at 5 µg/ml at *τ*_p_ = 1 min and *A*_avg_ = 80 molecules. The initial Itk (Itk^0^ = 140 molecules) and PIP_3_ concentrations (PIP_3_^0^ = 530 molecules) were used. The orange bar indicates R*^expt^*. (E) and (F) show maps of the most robust models (with the lowest D_KL_) as R*^expt^* and *A* (shown as *A*_avg_) are varied for the same parameters as in (C) and (D), respectively.

#### Experimental analysis of Itk activation kinetics in mouse thymocytes

To determine which Itk activation profile predicted by models M1–M7 produces maximum robustness while reproducing experimental data, we analyzed Itk activation kinetics in mouse CD4^+^CD8^+^ double-positive (DP) thymocytes, the developmental stage where positive selection occurs [9], [60]. To generate a homogeneous cell population in which every cell expresses the same TCR and in which the TCR has not been stimulated by endogenous ligands prior to *in vitro* stimulation, we used *OT1 TCR*-transgenic, *RAG2^−/−^*, *MHCI(β2m)* ^−/−^ mice. Their DP cells express exclusively the transgenic OT1 TCR, which recognizes the ovalbumin-derived peptide ligand OVA and recently identified endogenous peptide ligands presented by MHCI molecules [40], [61]. In *MHCI^−/−^* mice, no endogenous ligands are presented to OT1 TCR-transgenic T cells and their development is blocked at the DP stage due to impaired positive selection. *In vitro*, OT1 TCR transgenic DP cells can be stimulated with MHCI tetramers loaded with OVA peptide [40], [61]. Due to its high affinity for the OT1 TCR, OVA stimulation generates strong TCR signals and induces DP cell deletion. A number of OVA-derived altered peptide ligands (APL) have been generated which carry single or multiple amino acid substitutions compared to OVA. In the peptide series OVA>Q4R7>Q4H7>G4, such substitutions progressively reduce OT1 TCR affinity and signaling capacity [40]. Consequently, OVA and Q4R7 cause *OT1 TCR-transgenic* DP cell negative selection, whereas Q4H7 and G4 trigger positive selection.

We used MHCI tetramers presenting either one of these four peptides to stimulate *RAG2^−/−^MHC^−/−^ OT1 TCR-transgenic* DP cells for various time points. We analyzed PLCγ1 phosphorylation at Y_783_, normalized to total PLCγ1 protein levels, as a measure for Itk activation [2] (Fig. 3A). Stimulation by all peptides induced fast PLCγ1 phosphorylation already at 1 min which peaked at 2 min and then decreased over the next 60 min to very low levels which, however, were still above background levels in unstimulated cells. The decrease was fastest between 2 and 5 min and then progressively slowed down. As expected, overall levels of PLCγ1 phosphorylation progressively decreased with decreasing peptide affinity/signaling capacity in the order OVA>Q4R7>Q4H7>G4. An asymmetric peak shape with an extended right flank was preserved across all signal intensities. We calculated the peak durations (*τ_w_*), peak times (*τ_p_*) and asymmetry ratios *R = τ_w_/τ_p_* in Table 2 for stimulation with OVA, Q4R7, Q4H7 and G4, respectively. Consistent with preserved peak asymmetry, all ratios R were >1.

**Table 2:** Values of peak time, peak width, and asymmetry ratio R calculated from the PLCγ1 activation kinetics in Fig. 3 for different ligands.

Ligand	Pεακ τιμε(*τ*_p_) (min)	Pεακ ωιδτη(*τ*_w_) (min)	R
OVA	2.0	3.9	1.9
Q4R7	2.0	8.6	4.3
Q4H7	2.0	7.5	3.8
G4	2.0	4.3	2.1

#### Comparison between experiments and conclusions

The phospho-PLCγ1 levels (representing active Itk) for different affinity peptides peaked at *τ*_p_ = 2 mins with *R* values from 1.9–4.3 (Table 2). Therefore, we fixed *τ*_p_^expt^ = 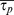 = 2 mins (the bar indicates average over the cell population) for quantifying robustness in the *in silico* modeling. The low, medium and large initial Itk and PIP_3_ concentrations represent stimulation by weak (G4), moderate (Q4R7, Q4H7) and high affinity (OVA) ligands, respectively. Analyzing D_KL_ (Fig. 3C) showed that for large initial PIP_3_ and Itk concentrations (representing OVA stimulation) the feedback models incorporating Itk PH domain dimers (M1–M3, M7) were substantially more robust (Smaller D_KL_ values) than the models lacking feedbacks (M5, M6) for small values of R (<3). Monomeric feedback model M4 produced large D_KL_ values (1.5–5). M5, M6 and M4 produced much larger ranges of R (Figure S14) as the parameters were varied compared to the feedback models with Itk dimers where the values of *R* were clustered around *R^expt^* ∼2. This behavior contributed substantially to the increased robustness of the feedback models with dimers as these models could accommodate for larger ranges of parameter variations while being able to maintain the constraint imposed by *R^expt^*. The relative robustness of the feedback versus feedback-free models showed similar qualitative trends for the other ligands, Q4R7, Q4H7, and G4 (Figure S15–S16). This suggests that the models containing feedbacks and Itk dimers are substantially more robust than models with Itk monomers or lacking feedbacks.

#### Evaluation of robustness in polyclonal thymocytes

The molecular wiring of Itk, PIP_3_ and IP_4_ interactions is unlikely to depend on the clonal nature of the T cells. Thus, the feedback models with Itk dimers should also be more robust than the other models when used to describe the kinetics of PLCγ1 activation in polyclonal DP thymocytes expressing many different TCRs with different ligand specificities, stimulated by antibodies against the common TCR subunit CD3 alone or with co-ligation of the common coreceptor CD4. Stimulation of non TCR-transgenic *MHC^−/−^* DP cells with 1 µg/ml or 5 µg/ml of αCD3 or combined αCD3/αCD4 antibodies produced different *R^expt^* and *τ*_p_ *^expt^* values than the OT1 system above (Fig. 4A–4B, Figure S18, Table. S16). Calculation of the robustness constrained by *R^expt^*, *τ*_p_ *^expt^* and *A* showed that feedback models M1, M2, M3 and M7 are again substantially more robust than the other models (Fig. 4C–4F, Figure S19, S20). Large variations of *R* in M4, M5 and M6 as parameters were varied again made these models substantially less robust than the feedback models with Itk dimers.

## Discussion

Here, we used *in silico* simulations combined with a novel Maximum Entropy (MaxEnt) based method and cell population averaged measurements of PLCγ1 activation kinetics to distinguish between multiple models constructed to elucidate different mechanisms of Itk activation in TCR signaling. Our analysis quantified the robustness of seven different models employing monomeric or dimeric Itk PH domains with or without positive and negative IP_4_ feedback against variations of parameters (rates and concentrations) at the single cell level. MaxEnt has been widely used in diverse disciplines ranging from physics [62] via information theory [63] to biology [64]–[67] to estimate probability distributions of variables subject to constraints imposed by experimental data [33], [65]. However, to our knowledge these methods have not been used for evaluating the robustness of dynamic models in cell signaling or gene regulatory systems. Using thymocyte positive selection as a physiologically important model process, our results show the usefulness of MaxEnt methods for such studies. We are currently working on extending the methods to include additional information from experiments (such as variances), and also evaluating their performance in comparison with closely related approaches such as Bayesian analysis [68].

Our simulations predict that the models containing IP_4_ feedbacks and Itk dimers are most robust. This is consistent with our previously proposed model of cooperative-allosteric regulation of Itk-PIP_3_ interactions via IP_4_-binding to oligomeric Itk PH domains [2]. Thymocyte selection critically depends on TCR induced signals. Small differences in antigen peptide concentration or affinities for the same TCR can produce opposite (negative vs. positive) selection outcomes [40]. Thus, we consider it plausible that for a fixed antigen dose and affinity (or average initial concentrations of Itk and PIP_3_ in our models), TCR signaling in thymocytes should be robust against cell-to-cell variations of protein/lipid concentrations, rate constants and local environment. But TCR signaling should retain sensitivity to small variations in antigen affinity or dose. A direct experimental validation of this assumption will require to test the probability distributions of *τ*_p_, *R*, and *A* in cell populations where PLCγ1 activation kinetics are measured in individual cells. However, we were unable to perform such single cell comparisons due to the insensitivity of FACS-based PLCγ1 signaling assays. This indicates the importance of studying the effects of network architecture, rate constants, protein and lipid concentrations on system robustness in DP thymocyte selection in detail in the future. Thymocytes are an excellent *in vivo* model to probe the exquisite dependency of cell fate decisions on the affinity of TCR ligands with important physiological and pathological implications. This provides a valuable addition to the experimental and theoretical investigations of robustness in synthetic systems or transformed tissue culture cells *in vitro.*

On the basis of robustness, our simulations support bimodal positive and negative Itk regulation by IP_4_ in thymocytes. They make a supportive argument that Itk PH domain oligomerization and IP_4_ feedback are physiologically important, consistent with the severely defective TCR signaling, IP_4_ production, Itk/PLCγ1 activation, positive selection and resulting immunodeficiency in *ItpkB^−/−^* mice, the ability of IP_4_ to bimodally control Itk PH domain binding to PIP_3_ *in vitro*, and the reported Itk PH domain oligomerization [2], [6], [7], [28]. They do, however, not exclude the possibility that IP_4_ also has additional, unknown functions in DP cells [14].

Testing this exciting hypothesis will require currently impossible single-cell measurements of IP_4_ levels in large cell populations. Moreover, the physiological roles and modes of Itk oligomerization, the specific PH domain contributions to Itk oligomerization, whether Itk oligomerization occurs in the cytoplasm or at the plasma membrane or both, whether it exclusively inhibits or can also promote Itk activation, and whether IP_4_ promotes or inhibits Itk PH domain binding to PIP_3_ or does both depending on its local concentration are all matters of active debate [2], [22]–[28]. Their conclusive elucidation requires quantitative biophysical studies of full length Itk with or without mutational perturbation of individual and combined interactions among the different Itk domains implicated in its monomeric and oligomeric self-association, and the reconstitution of *Itk^−/−^* mice with these mutants at endogenous expression levels. Unfortunately, difficulties to produce sufficient quantities of soluble full-length Itk or Itk PH domain protein, and a tendency of Itk and its PH domain to aggregate *in vitro* have precluded more quantitative analyses of Itk PH domain oligomerization and IP_4_/PIP_3_ interactions, as well as the generation of non-oligomerizing Itk PH domain mutants. Despite progress regarding SH2/SH3/proline-rich domain interactions [22]–[26] and some evidence for PH domain involvement [2], [27], [28], formation of several different homotypic Itk dimers with differing subcellular localization and functions further complicates such analyses and their interpretation. Our *in silico* results suggest that by enabling competing positive and negative IP_4_ induced feedback, Itk PH domain oligomerization could render Itk signaling in DP thymocytes much more robust to parameter fluctuation between individual cells than could be achieved without Itk dimers, or without IP_4_ feedback. Models M1–M3 and M7 involving Itk dimers and IP_4_ feedbacks showed substantially larger robustness than models lacking feedbacks (M5–M6) or containing only monomeric Itk (M4). M1–M3 and M7 can describe the experimentally observed PLCγ1 kinetics with similar robustness. They differ only at the level of secondary Itk/IP_4_/PIP_3_ interactions. Similar robustness and the inherent variability of experimental data preclude the identification of one of these dimeric Itk feedback models as the only one operative *in vivo* thus far.

## Materials and Methods

### Signaling Kinetics in the *in silico* Models

We constructed ODE based models. The ODEs described kinetics of concentrations of proteins and lipids in two well-mixed compartments representing plasma membrane and cytosol (Figure S1A). The biochemical signaling reactions for each model are shown in Tables S1–S7. The details regarding the construction of the ODEs and the parameters are given in the web supplement and Figure S1. We use the rule based modeling software package BioNetGen [69] to generate time courses for the species kinetics for the signaling networks described by models M1–M7. This program produces a set of ODEs corresponding to the mass-action kinetics describing biochemical reactions in the networks and solves them numerically using the CVODE solver [70]. The ODEs for each model are listed in the supplementary material.

### Quantification of Robustness Based on the Maximum Entropy Principle

When a variable *x* can assume multiple values and is distributed according to a probability distribution *p*(*x*), then the uncertainty associated with the distribution can be quantified by the entropy (S) defined as,
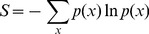
(1)


*S* is non-negative and is maximized when *x* is distributed according to a uniform distribution (i.e., *x* can take any value within a range with equal probability). Suppose *p(x)* is unknown, but we do know the average value of a variable, *f*, that is a function of *x, i.e., f = f(x)*. We can then maximize S under the constraint
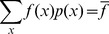
(2)


The constrained MaxEnt distribution is given by *p*(*x*) ∝ exp(−λ*x*), where the constant λ, αλσο κνοων as the Lagrange multiplier, is determined by solving Eq. (2) for λ when the above MaxEnt distribution for *p*(*x*) is used in Eq. (2). The method can be easily generalized to accommodate multiple variables and constraints. We used the constraints imposed by *τ*_p_^expt^, *R* ^expt^, and *A* ^expt^, or, *τ*_p_^expt^ and *R* ^expt^ that are measured over a cell population. Therefore, the MaxEnt distribution of the parameters in our calculation is given by, *p*({*k*_i_}) ∝ exp(−λ_1_ *τ*_p_({*k*_i_}) – λ_2_ *R*({*k*_i_}) − λ_3_ *A*({*k*_i_})), where λ_1_, λ_2_ ανδ λ_3_ denote the Lagrange’s multipliers, and {*k*_i_} denote the values of rate constants and initial concentrations in individual cells. The Lagrange multipliers can be calculated from the constraint equations,
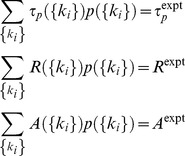
(3)


The MaxEnt distribution thus describes how *τ*_p_, *R*, and *A*, in individual cells are distributed over a cell population. The distribution also produces an estimation of the probability distributions for the rate constants and initial concentrations that regulate τ_p_, *R*, and *A*, through the functions *τ*_p_({*k*_i_}), *R*({*k*_i_}), and, *A*({*k*_i_}), respectively. The specific relationship between the parameters, {*k*_i_}, and the observables (*τ*_p_, *R*, and *A*) is dependent on the molecular details of the models, M1–M7. In all the models prior to the MaxEnt calculation, the rate constants were chosen from a uniform distribution with lower and upper bounds equal to 1/10 and 10 times, respectively, of the base values shown in Tables S1–S7. Similarly, the initial concentrations of proteins (e.g., Itk) and lipids (such as PIP_3_) were varied within a 35% [71] range from uniform distributions centered at the base values shown in Table S8. The joint uniform distribution in the parameters is given by q({*k*_i_}). We then used these MaxEnt distributions to quantify relative robustness of the models by calculating the Kullback-Leibler distance [59]

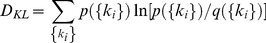
(4)


That is, for each model, we first find the probability distribution for the rate constants and initial concentrations that maximizes the entropy (robustness) for that model under the experimental constraints, giving the model a kind of “maximum benefit of the doubt.” We then compare the resulting MaxEnt models with one another to evaluate their relative robustness to variation in the rate constants, in order to select the model(s) most likely to correctly represent the actual kinetics. When *p*({*k*_i_}) is equal to *q*({*k*_i_}), *D*_KL_ assumes the minimum value 0; as the distribution *p*({*k*_i_}) starts deviating from the uniform distribution, say by becoming sharply peaked around a particular value, *D*_KL_ increases. Thus maximizing the entropy S, is equivalent to minimizing *D*_KL_ in Eq. (4). The calculations of *D*_KL_ were done at a specific antigen dose which fixed the average values of initial concentrations of Itk and PIP_3_. Therefore, the robustness calculations did not exclude the sensitivity of PLCγ1 activation to changes in PIP_3_ concentrations resulting from antigen dose variations. We calculated *p*({*k*_i_}) by by minimizing the *D*_KL_ subject to the constraints imposed by Eq. (3). We used *D*_KL_ to rank order the models for a particular measured value of *τ_p_^expt^*, R^expt^, and, *A*_avg_. All the calculations were carried out using MATLAB. Additional details can be found in the supplementary material (Figures S12–S15). Note that *D*_KL_ is unaffected by inclusion of additional parameters that do not influence the experimentally measured variables (Figure S21, Table S17). Thus having extra variables in a model does not in and of itself affect the relative robustness of models with variable numbers of parameters. We have used 100,000 sample points, which we have shown to be statistically sufficient in Figure S22 for the faithful calculation of *D*_KL_.

### Thymocyte Stimulation and Immunoblot Analysis

All mice were housed in The Scripps Research Institute specific pathogen-free vivarium monitored by The Scripps Research Institute Department of Animal Resources. All animal studies were approved by The Scripps Research Institute IACUC and conform to all relevant regulatory standards.

DP cells were prepared as in [2] and rested at 37°C for 3 hours. Then, 10^7^ DP cells per sample were incubated on ice for 15 min with 2.4 µM MHCI tetramers pre-loaded with either one of the altered peptide ligands OVA, Q4R7, Q4H7 or G4 [40], stimulated by rapidly adding 37°C warm PBS for the indicated times and quickly lysed in 100 mM Tris, pH 7.5, 600 mM NaCl, 240 mM n-octyl-β-D-glucoside, 4% Triton, 4 mM EDTA and a protease/phosphatase inhibitor cocktail (Roche). Lysates were cleared by centrifugation at 14000 rpm for 10 minutes at 4°C, resolved by SDS-PAGE and analyzed via immunoblot as previously described [2]. Band intensities were quantified via densitometry using NIH *ImageJ* software, and phosphoY_783_-PLCγ1 intensities normalized to total PLCγ1 amounts.

## Supporting Information

Figure S1 (A) Details of the simulation box. We used L = 2 µm, *l* = 2 nm and d = 0.02 µm for our simulations. **(B) Graphical networks describing the signaling reactions in models M1–M7.** Itk shown in this figure represents an Itk molecule that is bound to the TCR and LAT signalosome (not shown). High affinity binding reactions are shown as green arrows. PIP_2_ hydrolysis into DAG and IP_3_ which ultimately produces IP_4_ (S) is shown as red arrows. (M1) In model M1, both IP_4_ and PIP_3_ can equally induce allosteric modifications of the PH domains in Itk dimers. (M2) Model M2. Similar to M1, however, modification of the PH domains by PIP_3_ cannot stabilize IP_4_ or PIP_3_ binding to the Itk PH domains. (M3) Model M3. Similar to M1, however, modification of the PH domains by PIP_3_ can only stabilize IP_4_ but not PIP_3_ binding to the Itk PH domains. (M4) Model M4. The Itk PH domains are monomeric and unable to interact allosterically. IP_4_ or PIP_3_, upon binding with a weak affinity, instantaneously changes Itk to a high affinity conformation (Itk^*^) where IP_4_ (or PIP_3_) can replace PH domain bound PIP_3_ (or IP_4_) with high affinity. (M5) Model M5. Both IP_4_ and PIP_3_ bind to the PH domains of the Itk dimer with low affinity. No allosteric modification occurs. (M6) Model M6. Similar to model M5 but Itk exists only in monomers. (M7) Model M7. Similar to M1, however, modification of the PH domains by PIP_3_ can only stabilize PIP_3_ but not IP_4_ binding to the Itk PH domains.
(TIF)

Figure S2 **Presence of Intrinsic fluctuations does not lead to qualitatively different temporal profiles as compared with the deterministic model.** We show 11 different stochastic trajectories for Itk^0^ = 20 molecules and PIP_3_^0^ = 50 molecules, the lowest concentration used in our simulations, for model M3. The stochastic trajectories for concentrations of PIP_3_ bound Itk were obtained by solving the Master equation associated with the signaling reactions (Table S3) using the Gillespie algorithm. The curve in red is the solution of the mass action kinetics given by a set of ODEs. We use the same kinetic rates and initial concentrations for the stochastic simulations and the ODEs.
(TIFF)

Figure S3 **Comparison between the ODE solutions and the stochastic trajectories averaged over a small number of cells.** We compared the temporal profiles of concentrations of PIP_3_ bound Itk obtained in simulations including stochastic copy number variations due to intrinsic noise fluctuations (red) with the solutions of the deterministic mass action reaction kinetics that ignored such fluctuations (solid black lines). The stochastic simulations were carried out by using Gillespie’s method which provided exact numerical solution of the Master equations associated with the models. We used the same rate constants and initial concentrations for the stochastic simulations and ODE solutions. The kinetic trajectories were averaged over 500 realizations (or *in silico* “cells”) for the stochastic simulations. We show the results for the smallest concentrations of Itk^0^ (20 molecules) and PIP_3_^0^ (50 molecules) where the effect of the stochastic fluctuations is expected to be the largest.
(TIFF)

Figure S4 Variation of the peak value (*A*) with Itk^0^ and PIP_3_^0^ for all seven models.
(TIFF)

Figure S5 **Variation of τ_p_ with Itk^0^ and PIP_3_^0^ for all six models.** The peak time (τ_p_) of the temporal profile of the concentration of PIP_3_ bound Itk varied by an order of magnitude (roughly from 1 min to 10 mins) in models M1–M4 and M7, while the peak time did not change appreciably in models M5 and M6 over the entire range of variation. However,τ_p_ did not vary appreciably over a large range of initial Itk (>100) and PIP_3_ concentrations (>150) even in the models M1–M4 and M7. Most of the large variations occurred at small concentrations of Itk and PIP_3_.
(TIFF)

Figure S6 **Estimation of the reaction rates in the effective binding-unbinding reaction.** A) The transient kinetics of PIP_3_ bound Itk in M3 (red) is compared with the case when the negative feedback is removed (black). We use τ_1/2_ and the steady state concentration of the kinetics of PIP_3_ bound Itk in the absence of the negative feedback to calculate the rates in the effective binding-unbinding reaction 1. B) Kinetics of PIP_3_ bound Itk in the absence of the negative feedback in model M3 (black). Blue, kinetics of PIP_3_ bound Itk in the corresponding binding unbinding process where the τ_1/2_ and the steady state concentration of PIP_3_ bound Itk is exactly the same as the black curve. (See Text S1)
(TIFF)

Figure S7 **Variation of K_D_ as a function of the sum of Itk^0^ and PIP_3_^0^ for models M1 to M4.** The K_D_ for the binding unbinding process has been estimated using the steady state values of the Itk kinetics in presence of the positive but not negative feedback. For models M1–M3, K_D_ does not change significantly with increasing concentrations of initial Itk and PIP_3_. The value of K_D_ is much smaller than the sum of (Itk^0^+PIP_3_^0^) as well. For M4 however, K_D_ increases significantly (by an order of magnitude). The absolute value of the K_D_ is still a lot less than (Itk^0^+PIP_3_^0^).
(TIFF)

Figure S8 **Variation of k_1_ as a function of the sum of Itk^0^ and PIP_3_^0^ for models M1 to M4.** *k_1_* decreased roughly 2 fold with the increase in Itk^0^ and PIP_3_^0^ for M1 and M3, while, for model M2, *k_1_* increased 4 times. In M4, *k_1_* did not change appreciably.
(TIFF)

Figure S9 **The saturation of the width in the feedback models.** A) We have varied both Itk^0^ and PIP_3_^0^ such that PIP_3_^0^ ≥ Itk^0^. The plot of the width of PIP_3_ bound Itk as a function of (Itk^0^+PIP_3_^0^) is shown for M1 (black line) and M2 (red line). For large values of (Itk^0^+PIP_3_^0^) the width saturates (the orange oval) both for M1 and M2. For M2 however the rate of decay of the width of Itk – PIP_3_ kinetics is much faster than for M1 as can be seen from the fact that the red curve decays from roughly 12 mins to 3 mins where as the black curve goes down from 7 mins to 5 mins. B) The transient activation kinetics of the membrane bound Itk in M1 are shown in black. PIP_3_^0^ = 500, Itk^0^ = 200. The dotted red curve is the exponential decay curve of the form e^−*kt*^ with the time constant equal to the inverse of the high affinity PIP_3_ unbinding rate.
(TIFF)

Figure S10 **A large concentration of IP_4_ is required to replace PIP_3_ in models M5–M6.** A) Variation of the steady state *x*_s_ (Itk-PIP_3_) as a function of initial substrate (PIP_2_) concentration *S* ^0^ when the K_D_ = 2000. B) Variation of the steady state *x*_s_ as a function of initial substrate concentration *S* ^0^ when the K_D_ = 200.
(TIFF)

Figure S11 **A large concentration of** ***IP_4_*** **is required to replace** ***PIP_3_*** **in model M4.** A) Variation of the steady state *x_s_* (Itk-PIP_3_) as a function of initial substrate (PIP_2_) concentration *S* ^0^ when the K_D_ = 2000. B) Same as in A) for K_D_ = 200.
(TIFF)

Figure S12 **The histograms for** ***R*** **and τ as the parameters are varied in all 7 models for moderately low initial concentrations of Itk^0^ and PIP_3_^0^.** All the rate constants are varied by two orders of magnitude with the constraint K_D_^low^ = α K_D_^high^. For M1–M3, α is distributed uniformly over 1 to 4000 while for M7 it is distributed uniformly over 1 to 50. The initial concentrations of species involved are varied in a 35% window about the base value of Itk^0^ = 40, PIP_3_^0^ = 130 and PIP_2_^0^ = 17000.
(TIFF)

Figure S13 **The histograms for R and τ as the parameters are varied in all 7 models for moderately high initial concentrations of Itk^0^ and PIP_3_^0^.** All the rate constants are varied by two orders of magnitude with the constraint K_D_^low^ = α K_D_^high^. For M1–M3, α is distributed uniformly over 1 to 4000 while for M7 it is distributed uniformly over 1 to 50. The initial concentrations of species involved are varied in a 35% window about the base value of Itk^0^ = 100, PIP_3_^0^ = 370 and PIP_2_^0^ = 17000.
(TIFF)

Figure S14 **The histograms for R and** τ **as the parameters are varied in all 7 models for high initial concentrations of Itk^0^ and PIP_3_^0^.** All the rate constants are varied by two orders of magnitude with the constraint K_D_^low^ = α K_D_^high^. For M1–M3, α is distributed uniformly over 1 to 4000 while for M7 it is distributed uniformly over 1 to 50. The initial concentrations of species involved are varied in a 35% window about the base value of Itk^0^ = 140, PIP_3_^0^ = 530 and PIP_2_^0^ = 17000.
(TIFF)

Figure S15 **Checkerboard plot of the most robust models for different ligand affinities as R_avg_ and A_avg_ are varied for a fixed τ_avg_ = 2 mins.** a) Plot of the most robust models for Itk^0^ = 140 and PIP_3_^0^ = 530 molecules. b) The same plot as a) for Itk^0^ = 100 and PIP_3_^0^ = 370 molecules. c) Same plot as a) for Itk^0^ = 40 and PIP_3_^0^ = 130 molecules. d) The same plot as a) for Itk^0^ = 20 and PIP_3_^0^ = 50 molecules.
(TIFF)

Figure S16 **Plots of the relative robustness of all the 7 models for a specific A_avg_ for different ligand affinities as R_avg_ is varies for a fixed τ_avg_ = 2 mins.** a) For Itk^0^ = 140 and PIP_3_^0^ = 530 molecules the D_KL_ is shown for an A_avg_ of 40 molecules. b) The same plot as a) for Itk^0^ = 100 and PIP_3_^0^ = 370 molecules when the A_avg_ is held fixed at 20 molecules. c) Same plot as a) for Itk^0^ = 40 and PIP_3_^0^ = 130 molecules when A_avg_ = 10 molecules. d) The same plot as **a)** for Itk^0^ = 20 and PIP_3_^0^ = 50 molecules when A_avg_ = 3 moelcules. The orange vertical bar in all the plots show the experimentally observed value of R_avg_.
(TIFF)

Figure S17 **The effect of Lck mediated phosphorylation of Itk-PIP_3_ on the relative robustness of M1–M7.** Upper panel (left most corner): For Itk^0^ = 100 and PIP_3_^0^ = 370 the most robust models are shown as amplitude and the ratio of the Itk-PIP_3_ kinetics are varied in presence of the Lck mediated phosphorylation of membrane recruited Itk at its Y511 residue. The average peak time is held at 2 mins. Upper panel (right most corner): The same plot without any Lck mediated activation. Lower panel (left most corner): The relative robustness of the models M1–M7 for an amplitude average of 20 molecules in presence of Lck mediated activation of Itk. Lower panel (right most corner): Same plot without the explicit Lck mediated activation.
(TIFF)

Figure S18 Kinetics of induction of PLCγ1 phosphorylation represented as the fold increase over non stimulated cells using total PLCγ1 protein as a loading control.
(TIFF)

Figure S19 **Checkerboard plot of the most robust models as R_avg_ and A_avg_ are varied for different doses of anti-CD3 and anti-CD3/CD4 antibodies.** a) Itk^0^ = 40 and PIP_3_^0^ = 130 molecules are used to emulate the 1 µg/mL anti CD3 stimulation. The τ_avg_ is held at 1 mins. The checkerboard diagram of the most robust models is shown as R_avg_ and A_avg_ are varied. b) Same as plot a) but Itk^0^ = 100 and PIP_3_^0^ = 370 molecules are used as the initial concentrations. c) Itk^0^ = 100 and PIP_3_^0^ = 370 molecules are used to emulate the 1 µg/mL anti CD3/CD4 stimulation. The τ_avg_ is held at 5 mins. The checkerboard diagram of the most robust models is shown as R_avg_ and A_avg_ are varied. d) Itk^0^ = 140 and PIP_3_^0^ = 530 molecules are used to emulate the 5 µg/mL anti CD3/CD4 stimulation. The τ_avg_ is held at 1 mins. The checkerboard diagram of the most robust models is shown as R_avg_ and A_avg_ are varied.
(TIFF)

Figure S20 **The plot of D_KL_ for all the 7 models for a specific amplitude and different initial conditions for different doses of anti CD3 or anti CD3/CD4 antibodies.** a) Itk^0^ = 40 and PIP_3_^0^ = 130 molecules are used to emulate the 1 µg/mL anti CD3 stimulation. The τ_avg_ is held at 1 mins. The D_KL_ is shown for an A_avg_ = 16 molecules. b) Same as plot a) but Itk^0^ = 100 and PIP_3_^0^ = 370 molecules are used as the initial concentrations and A_avg_ = 60 molecules. c) Itk^0^ = 100 and PIP_3_^0^ = 370 molecules are used to emulate the 1 µg/mL anti CD3/CD4 stimulation. The τ_avg_ is held at 5 mins. A_avg_ = 60 molecules. d) Itk^0^ = 140 and PIP_3_^0^ = 530 molecules are used to emulate the 5 µg/mL anti CD3/CD4 stimulation. The τ_avg_ is held at 1 mins and A_avg_ is set equal to 80 molecules. The vertical orange bar shows the observed experimental values.
(TIFF)

Figure S21 **Addition of parameters which weakly affect the Itk-PIP_3_ kinetics, do not lead to any significant difference in the D_KL_.** For Itk^0^ = 100 and PIP_3_^0^ = 370, a) we have looked at the relative difference in the D_KL_ of our old M3 (black) and M3 with the added reactions (magenta) for an amplitude average of 30 molecules and peak time average of 2 mins. b) We have looked at the relative difference in the D_KL_ of our old M3 (black) and M3 with the added reactions (magenta) for an amplitude average of 40 molecules and peak time average of 2 mins.
(TIFF)

Figure S22 **The sample set of 100,000 is a good sample size.** We show the D_KL_ of M1–M7 for Itk^0^ = 100 and PIP_3_^0^ = 370 for a) 20,000 realizations and b) 100,000 realizations when the amplitude average is 20 molecules and the peak time average is 2 mins. The KL distances are identical.
(TIFF)

Figure S23 **D_KL_ without the constraint on amplitude.** Lower *D*KL values (shown in log10 scale) denote higher robustness for any given Ravg. Based on the data in Fig. 4, the average peak time was fixed at 2 mins in all cases. Experimentally measured Ravg values are indicated by vertical orange lines. (A) Robustness for models M1–M3 and M5–M6 at high initial Itk (Itk0 = 140 molecules) and PIP3 concentrations (PIP30 = 530 molecules), simulating high-affinity OVA stimulation. M2 appears most robust in the experimentally observed Rave range. M4 fails produce any R value in the range investigated here. (B) M2 shows maximal robustness for moderate concentrations of initial Itk ( = 100 molecules) and PIP3 ( = 370 molecules), simulating Q4R7 stimulation. (C) For lower values of Itk0 ( = 40 molecules) and PIP30 ( = 130 molecules), simulating Q4H7 stimulation, M1–M3 are most robust with similar *D*KL values in the experimentally observed Rave range. (D) For low initial concentrations of Itk (Itk0 = 20 molecules) and PIP3 (PIP30 = 50 molecules), simulating stimulation by the low affinity peptide G4, M1–M3 are again most robust inthe experimentally observed Ravg range. Models M4–M6 fail to produce any value of R in the range investigated here. Model M7 is not shown.
(TIFF)

Table S1 Reactions and rate constants for model M1.
(DOCX)

Table S2 Reactions and rate constants for model M2.
(DOCX)

Table S3 Reactions and rate constants for model M3.
(DOCX)

Table S4 Reactions and rate constants for model M4.
(DOCX)

Table S5 Reactions and rate constants for model M5.
(DOCX)

Table S6 Reactions and rate constants for model M6.
(DOCX)

Table S7 Reactions and rate constants for model M7.
(DOCX)

Table S8 Values of the concentrations of different molecular species used in the models.
(DOCX)

Table S9 Reactions and rate constants for model M1^lck^.
(DOCX)

Table S10 Reactions and rate constants for model M2^lck^.
(DOCX)

Table S11 Reactions and rate constants for model M3^lck^.
(DOCX)

Table S12 Reactions and rate constants for model M4^lck^.
(DOCX)

Table S13 Reactions and rate constants for model M5^lck^.
(DOCX)

Table S14 Reactions and rate constants for model M6^lck^.
(DOCX)

Table S15 Reactions and rate constants for model M7^lck^.
(DOCX)

Table S16 Values of peak time, peak width, and asymmetry ratio *R* calculated from the PLCγ1 activation kinetics in Figure S18.
(DOCX)

Table S17 New reactions added to M3.
(DOCX)

Text S1 Supporting calculations and discussions.
(DOCX)
