# Correction: *In Silico* Modeling of Itk Activation Kinetics in Thymocytes Suggests Competing Positive and Negative IP_4_ Mediated Feedbacks Increase Robustness

**DOI:** 10.1371/annotation/285bb79f-ef1a-467e-9fd3-4e518d65acd3

**Published:** 2014-01-06

**Authors:** Sayak Mukherjee, Stephanie Rigaud, Sang-Cheol Seok, Guo Fu, Agnieszka Prochenka, Michael Dworkin, Nicholas R. J. Gascoigne, Veronica J. Vieland, Karsten Sauer, Jayajit Das

In the Funding Statement, the grant from the NIH to the ninth author (K.S.) was incorrectly mentioned twice, and multiple grants were incorrectly omitted from the Funding Statement. The Funding Statement should read: "This work was supported by funding from the Research Institute at Nationwide Childrens Hospital, the NIH (AI090115), and the Ohio Supercomputer Center (OSC) to J.D., NIH grant AI070845 and The Leukemia and Lymphoma Society Scholar Award 1440-11 to K.S, and NIH grant MH086117 to V.J.V. Part of this work was supported by NIH grants DK094173 and GM065230 to N.R.J.G. The funders had no role in study design, data collection and analysis, decision to publish, or preparation of the manuscript."

In addition, there were multiple errors in the Materials and Methods. The first sentence of the third paragraph of the "Quantification of Robustness Based on the Maximum Entropy Principle" section of the Materials and Methods is incorrect. The correct sentence is: "The constrained MaxEnt distribution is given by α(x)/exp(—λx), where the constant λ, also known as the Lagrange multiplier, is determined by solving Eq. (2) for λ when the above MaxEnt distribution for p(x) is used in Eq. (2)." The fourth sentence of the same paragraph is also incorrect. The correct sentence is: "Therefore, the MaxEnt distribution of the parameters in our calculation is given by,

*p*({*k*_i_})αexp(—λ_1_τ_p_({*k*_i_})—λ_2_ *R*({*k*_i_}) — λ_3_ *A*({*k*_i_})), where λ_1_, λ_2_ and λ_3_ denote the Lagrange’s multipliers, and {*k*_i_} denote the values of rate constants and initial concentrations in individual cells." 

