# Supplementary figures and images for: In Silico Modeling of Itk Activation Kinetics in Thymocytes Suggests Competing Positive and Negative IP4 Mediated Feedbacks Increase Robustness
Source: PLoS One. 2013 Sep 16;8(9):e73937. doi: 10.1371/journal.pone.0073937 (PMC3774804; doi:10.1371/journal.pone.0073937)

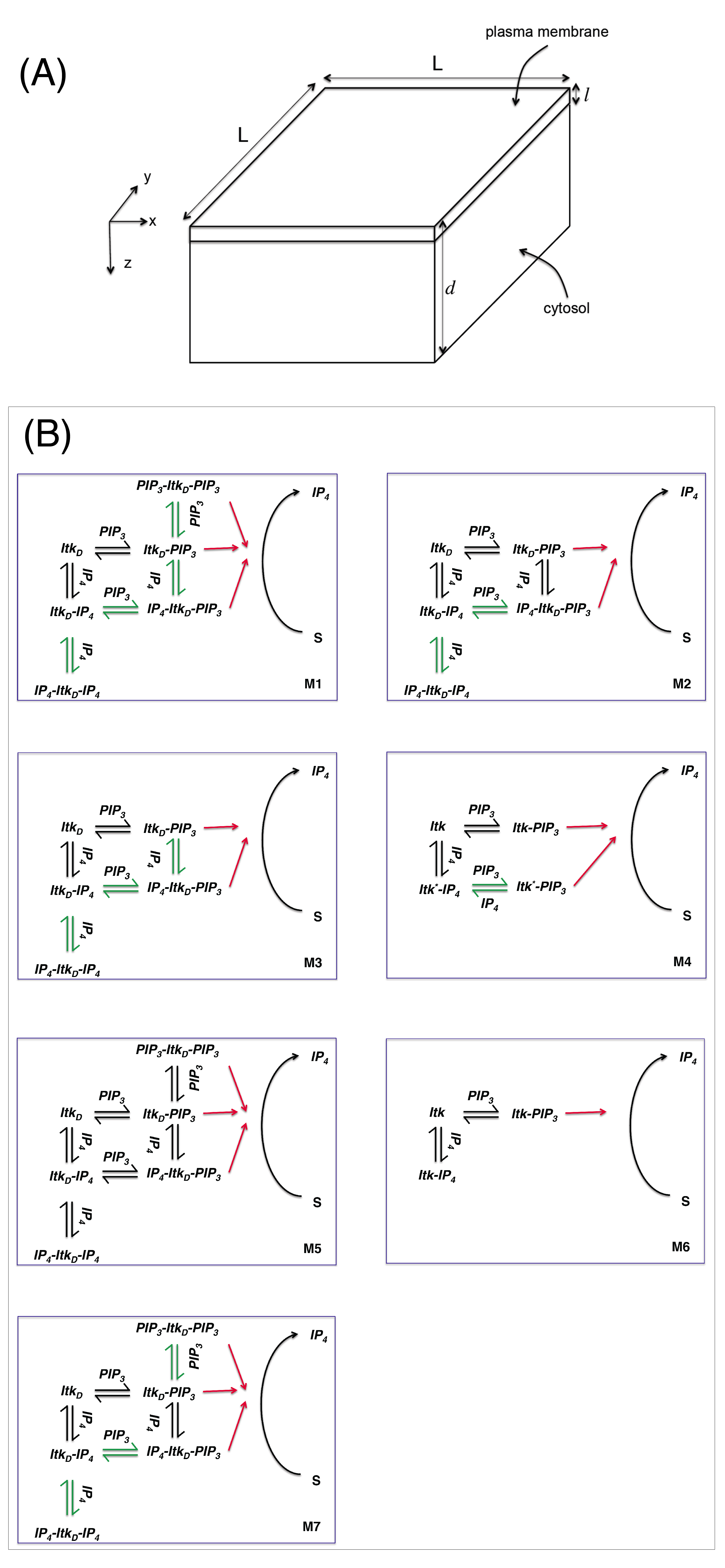

Supplement: Figure S1 — (A) Details of the simulation box. We used L = 2 µm, l = 2 nm and d = 0.02 µm for our simulations. (B) Graphical networks describing the signaling reactions in models M1–M7. Itk shown in this figure represents an Itk molecule that is bound to the TCR and LAT signalosome (not shown). High affinity binding reactions are shown as green arrows. PIP2 hydrolysis into DAG and IP3 which ultimately produces IP4 (S) is shown as red arrows. (M1) In model M1, both IP4 and PIP3 can equally induce allosteric modifications of the PH domains in Itk dimers. (M2) Model M2. Similar to M1, however, modification of the PH domains by PIP3 cannot stabilize IP4 or PIP3 binding to the Itk PH domains. (M3) Model M3. Similar to M1, however, modification of the PH domains by PIP3 can only stabilize IP4 but not PIP3 binding to the Itk PH domains. (M4) Model M4. The Itk PH domains are monomeric and unable to interact allosterically. IP4 or PIP3, upon binding with a weak affinity, instantaneously changes Itk to a high affinity conformation (Itk*) where IP4 (or PIP3) can replace PH domain bound PIP3 (or IP4) with high affinity. (M5) Model M5. Both IP4 and PIP3 bind to the PH domains of the Itk dimer with low affinity. No allosteric modification occurs. (M6) Model M6. Similar to model M5 but Itk exists only in monomers. (M7) Model M7. Similar to M1, however, modification of the PH domains by PIP3 can only stabilize PIP3 but not IP4 binding to the Itk PH domains. (TIF) [file pone.0073937.s001.tif]

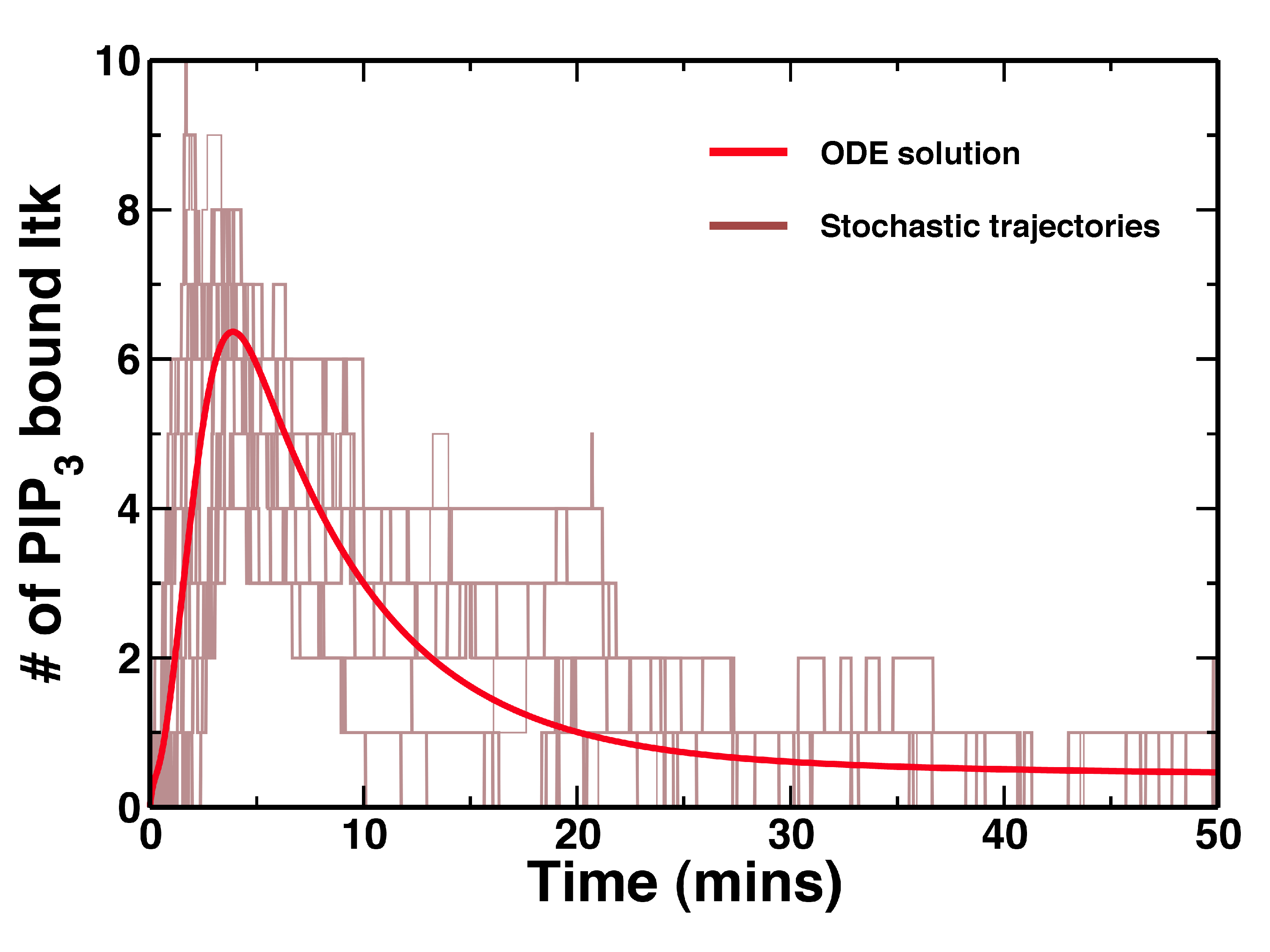

Supplement: Figure S2 — Presence of Intrinsic fluctuations does not lead to qualitatively different temporal profiles as compared with the deterministic model. We show 11 different stochastic trajectories for Itk0 = 20 molecules and PIP3 0 = 50 molecules, the lowest concentration used in our simulations, for model M3. The stochastic trajectories for concentrations of PIP3 bound Itk were obtained by solving the Master equation associated with the signaling reactions (Table S3) using the Gillespie algorithm. The curve in red is the solution of the mass action kinetics given by a set of ODEs. We use the same kinetic rates and initial concentrations for the stochastic simulations and the ODEs. (TIFF) [file pone.0073937.s002.tiff]

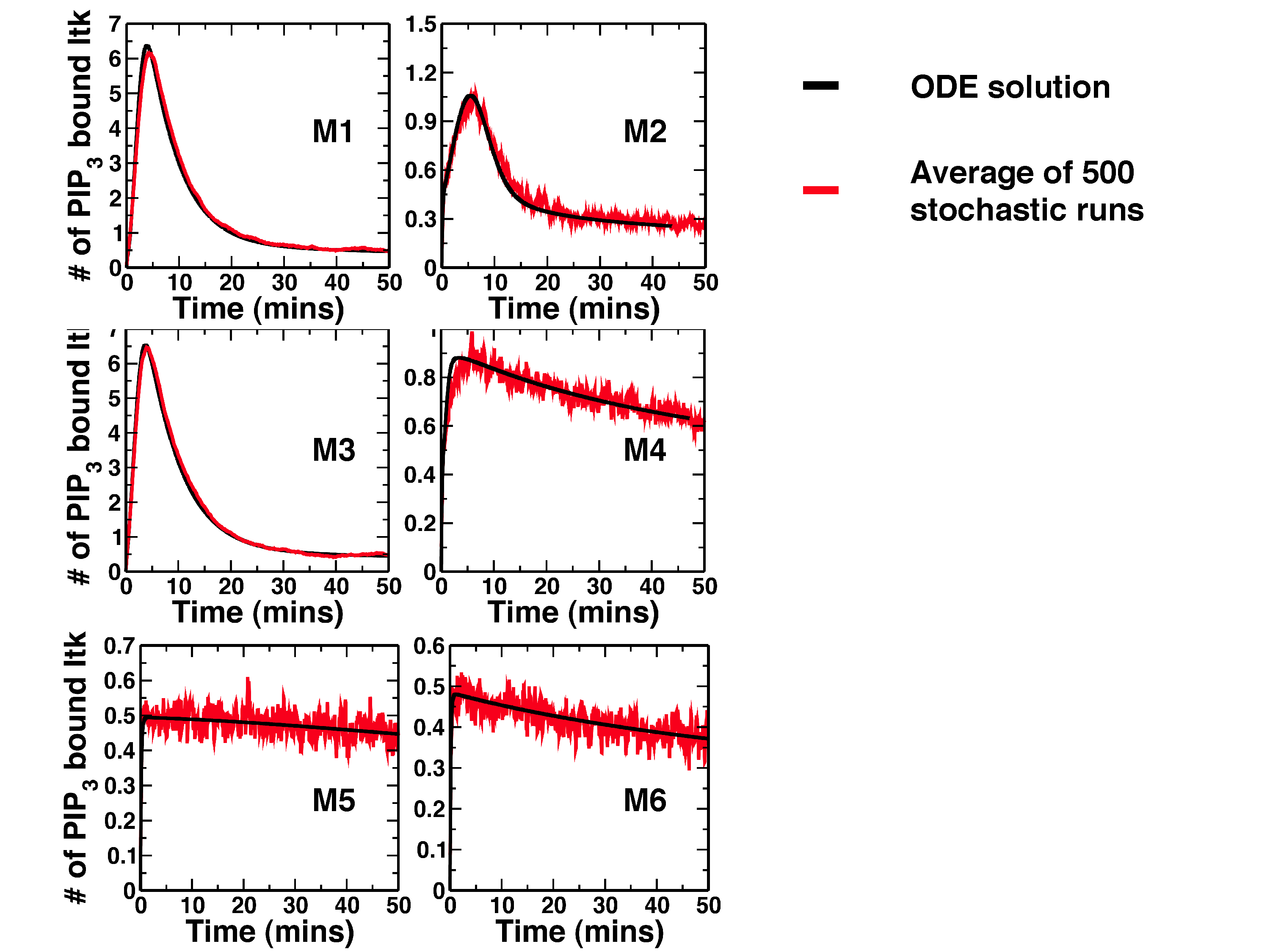

Supplement: Figure S3 — Comparison between the ODE solutions and the stochastic trajectories averaged over a small number of cells. We compared the temporal profiles of concentrations of PIP3 bound Itk obtained in simulations including stochastic copy number variations due to intrinsic noise fluctuations (red) with the solutions of the deterministic mass action reaction kinetics that ignored such fluctuations (solid black lines). The stochastic simulations were carried out by using Gillespie’s method which provided exact numerical solution of the Master equations associated with the models. We used the same rate constants and initial concentrations for the stochastic simulations and ODE solutions. The kinetic trajectories were averaged over 500 realizations (or in silico “cells”) for the stochastic simulations. We show the results for the smallest concentrations of Itk0 (20 molecules) and PIP3 0 (50 molecules) where the effect of the stochastic fluctuations is expected to be the largest. (TIFF) [file pone.0073937.s003.tiff]

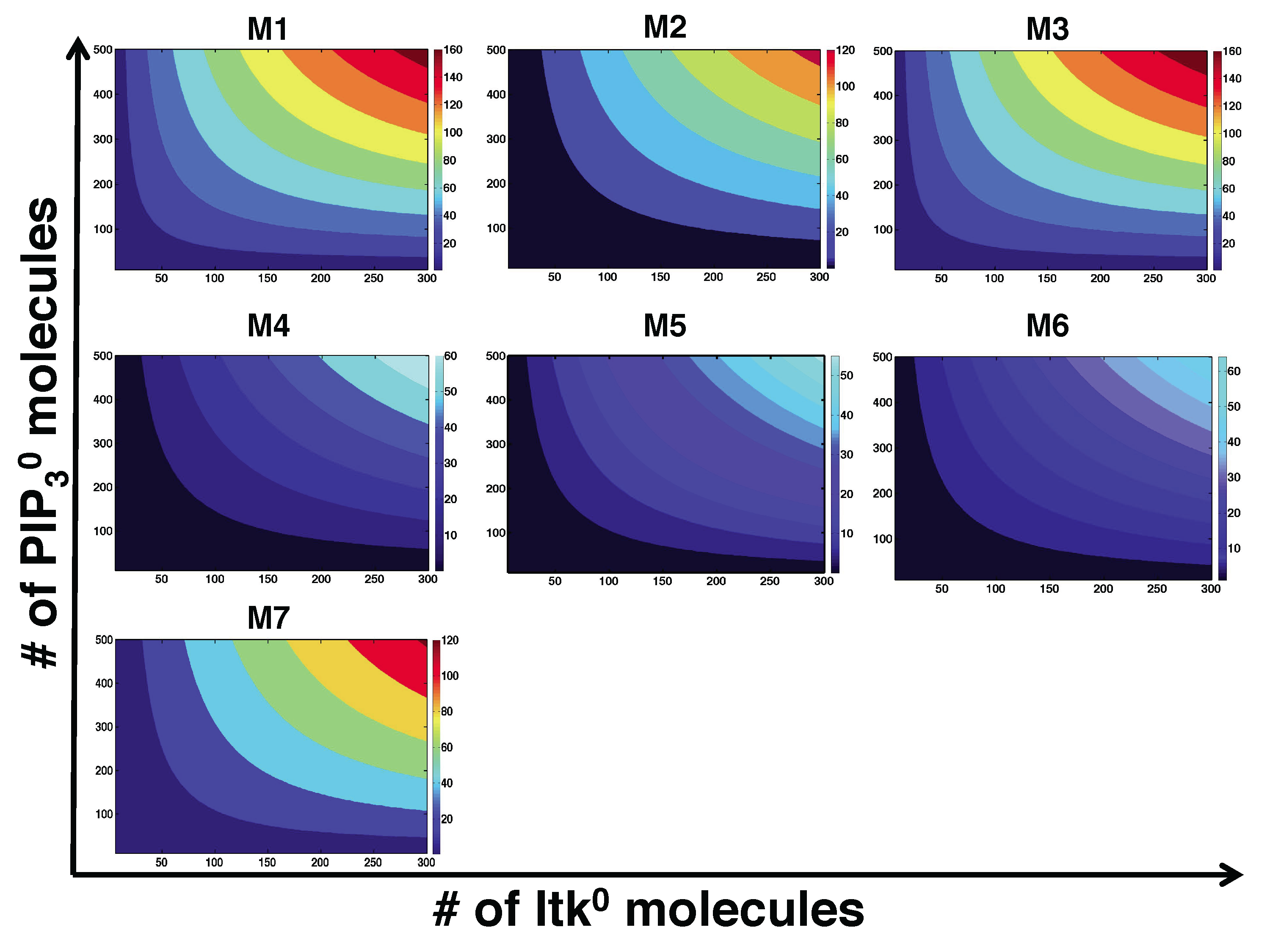

Supplement: Figure S4 — Variation of the peak value (A) with Itk0 and PIP3 0 for all seven models. (TIFF) [file pone.0073937.s004.tiff]

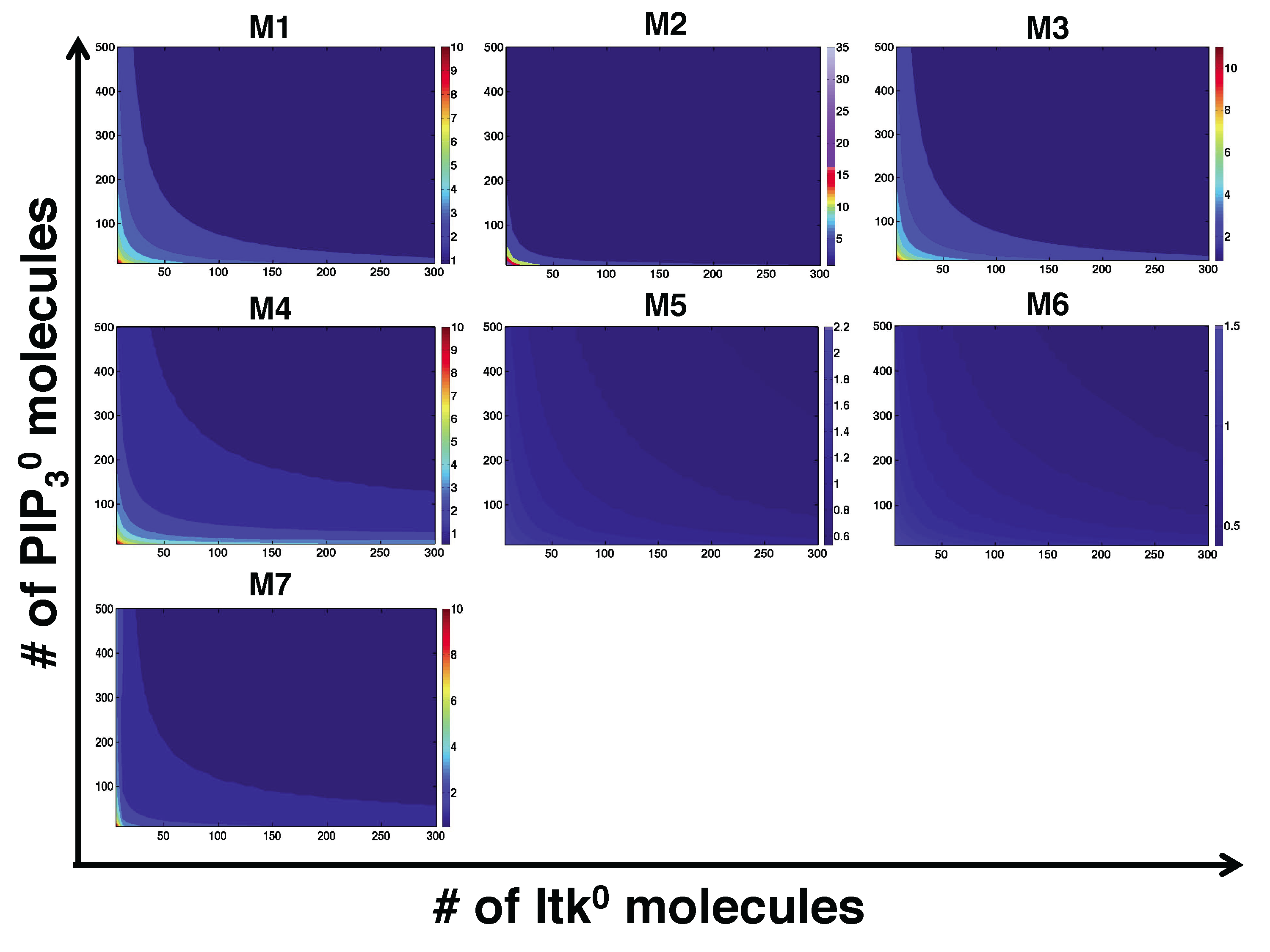

Supplement: Figure S5 — Variation of τp with Itk0 and PIP30 for all six models. The peak time (τp) of the temporal profile of the concentration of PIP3 bound Itk varied by an order of magnitude (roughly from 1 min to 10 mins) in models M1–M4 and M7, while the peak time did not change appreciably in models M5 and M6 over the entire range of variation. However,τp did not vary appreciably over a large range of initial Itk (>100) and PIP3 concentrations (>150) even in the models M1–M4 and M7. Most of the large variations occurred at small concentrations of Itk and PIP3. (TIFF) [file pone.0073937.s005.tiff]

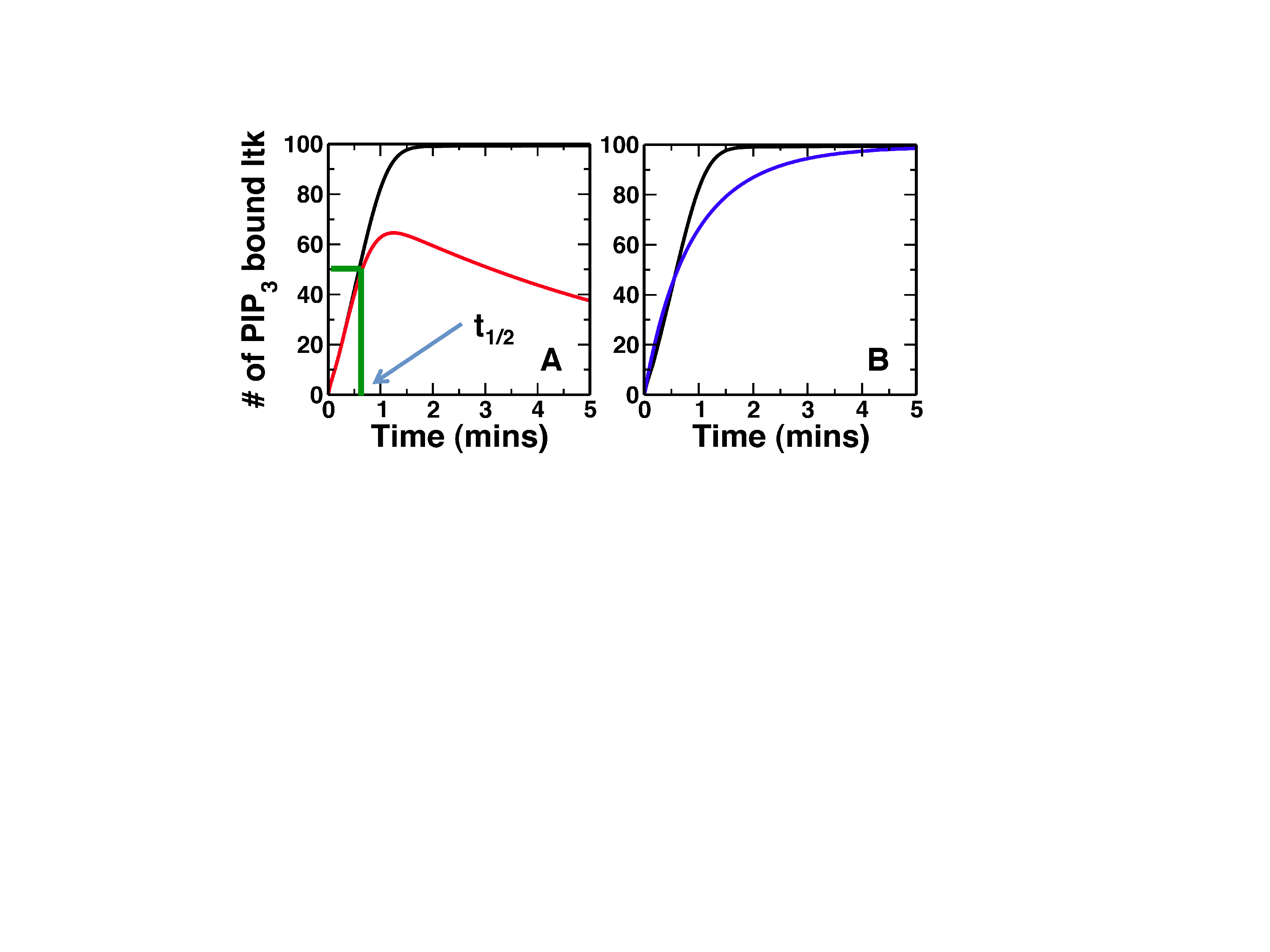

Supplement: Figure S6 — Estimation of the reaction rates in the effective binding-unbinding reaction. A) The transient kinetics of PIP3 bound Itk in M3 (red) is compared with the case when the negative feedback is removed (black). We use τ1/2 and the steady state concentration of the kinetics of PIP3 bound Itk in the absence of the negative feedback to calculate the rates in the effective binding-unbinding reaction 1. B) Kinetics of PIP3 bound Itk in the absence of the negative feedback in model M3 (black). Blue, kinetics of PIP3 bound Itk in the corresponding binding unbinding process where the τ1/2 and the steady state concentration of PIP3 bound Itk is exactly the same as the black curve. (See Text S1) (TIFF) [file pone.0073937.s006.tiff]

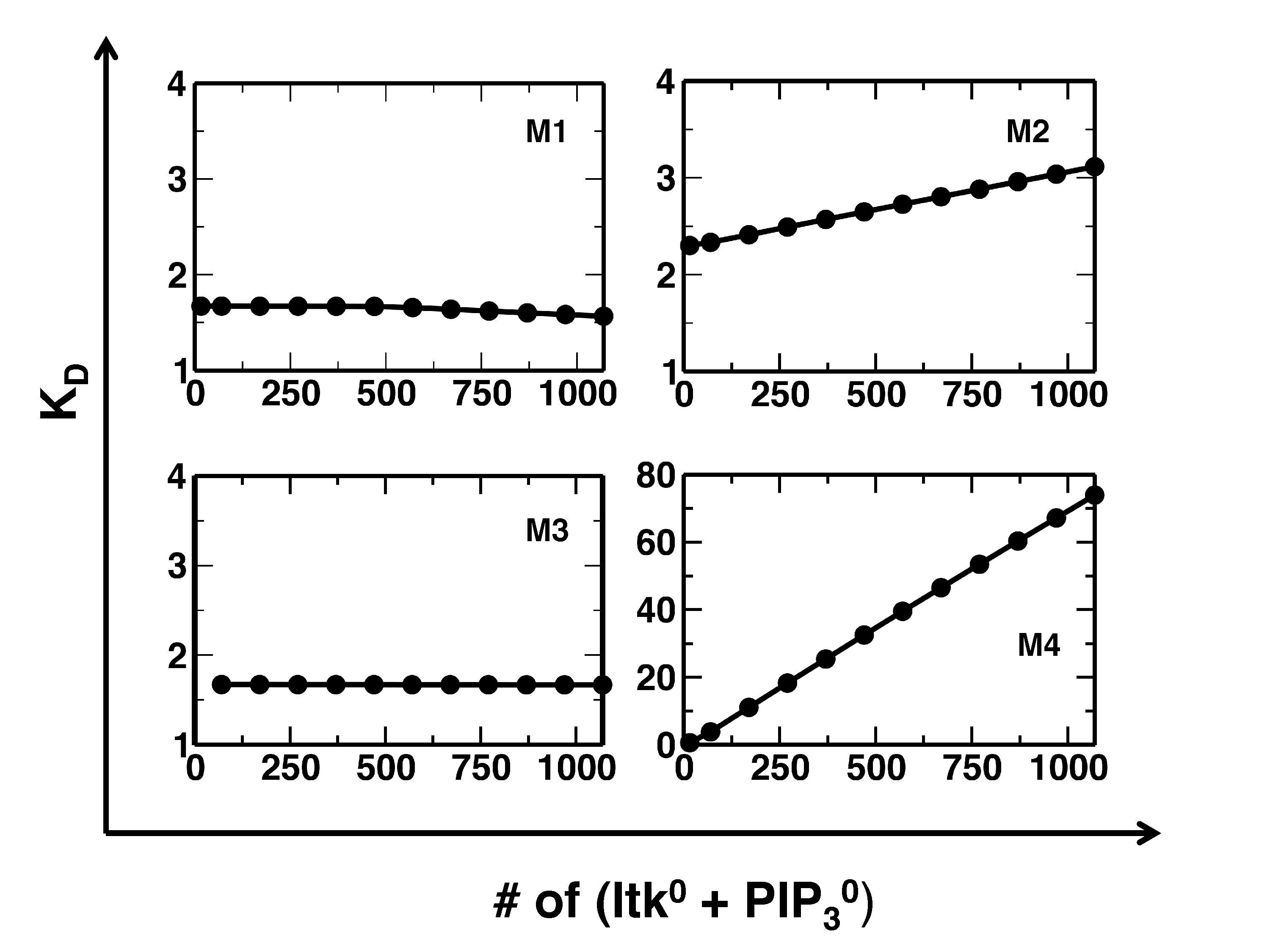

Supplement: Figure S7 — Variation of KD as a function of the sum of Itk0 and PIP30 for models M1 to M4. The KD for the binding unbinding process has been estimated using the steady state values of the Itk kinetics in presence of the positive but not negative feedback. For models M1–M3, KD does not change significantly with increasing concentrations of initial Itk and PIP3. The value of KD is much smaller than the sum of (Itk0+PIP3 0) as well. For M4 however, KD increases significantly (by an order of magnitude). The absolute value of the KD is still a lot less than (Itk0+PIP3 0). (TIFF) [file pone.0073937.s007.tiff]

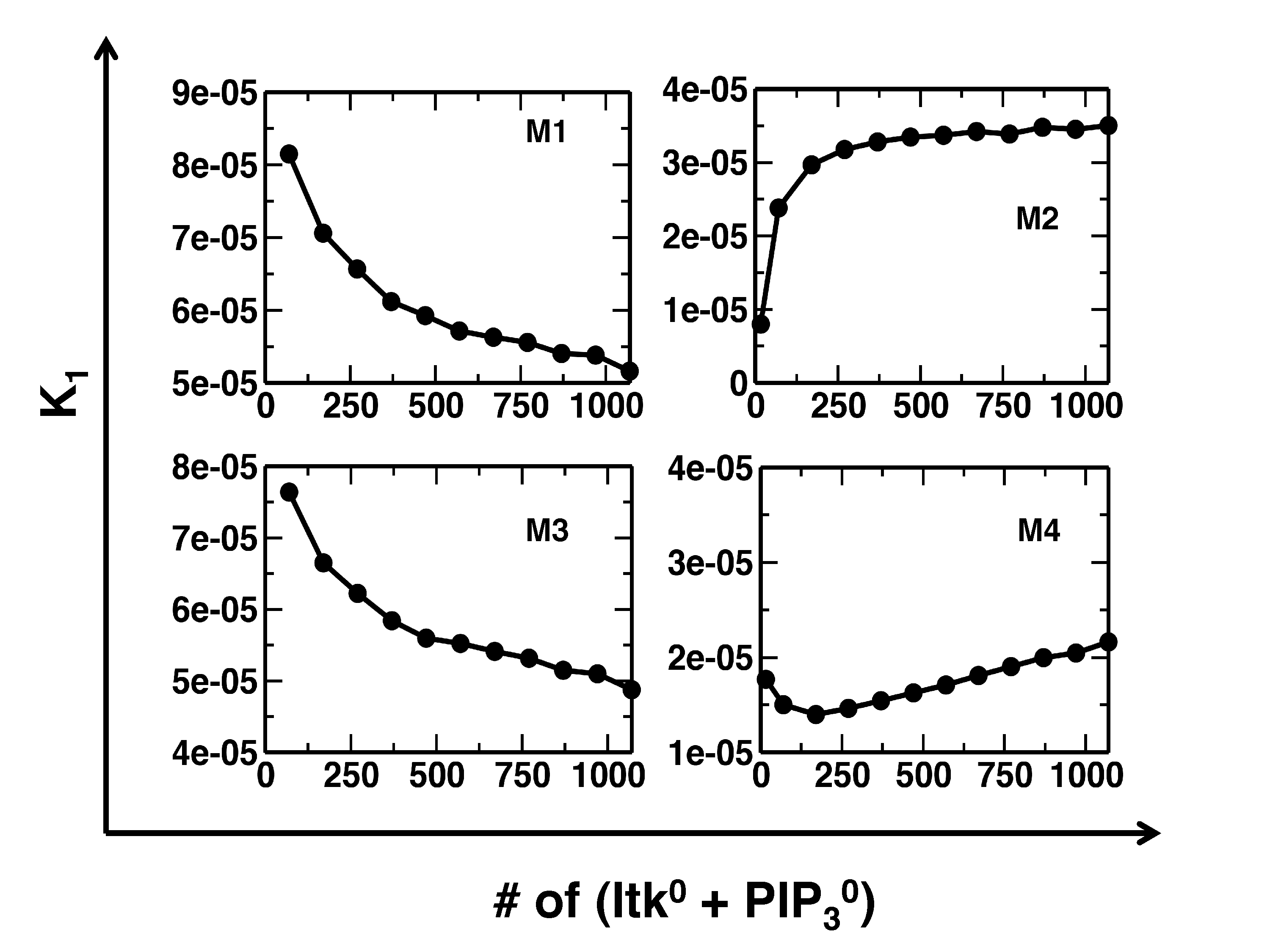

Supplement: Figure S8 — Variation of k1 as a function of the sum of Itk0 and PIP30 for models M1 to M4. k1 decreased roughly 2 fold with the increase in Itk0 and PIP3 0 for M1 and M3, while, for model M2, k1 increased 4 times. In M4, k1 did not change appreciably. (TIFF) [file pone.0073937.s008.tiff]

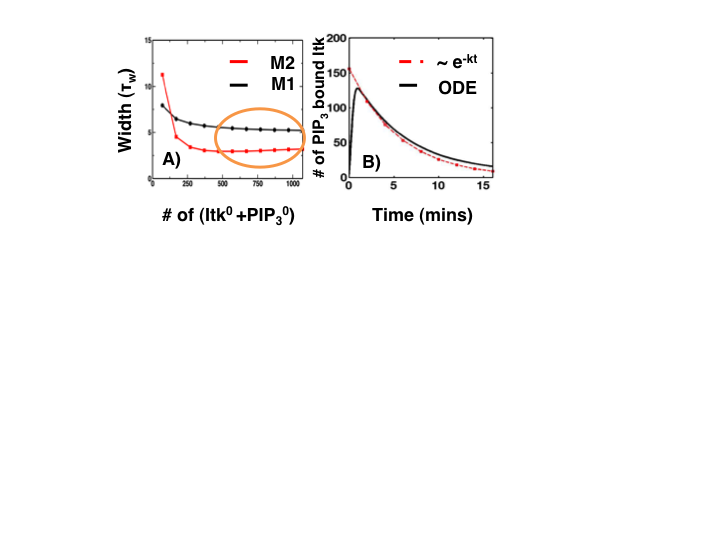

Supplement: Figure S9 — The saturation of the width in the feedback models. A) We have varied both Itk0 and PIP3 0 such that PIP3 0 ≥ Itk0. The plot of the width of PIP3 bound Itk as a function of (Itk0+PIP3 0) is shown for M1 (black line) and M2 (red line). For large values of (Itk0+PIP3 0) the width saturates (the orange oval) both for M1 and M2. For M2 however the rate of decay of the width of Itk – PIP3 kinetics is much faster than for M1 as can be seen from the fact that the red curve decays from roughly 12 mins to 3 mins where as the black curve goes down from 7 mins to 5 mins. B) The transient activation kinetics of the membrane bound Itk in M1 are shown in black. PIP3 0 = 500, Itk0 = 200. The dotted red curve is the exponential decay curve of the form e−kt with the time constant equal to the inverse of the high affinity PIP3 unbinding rate. (TIFF) [file pone.0073937.s009.tiff]

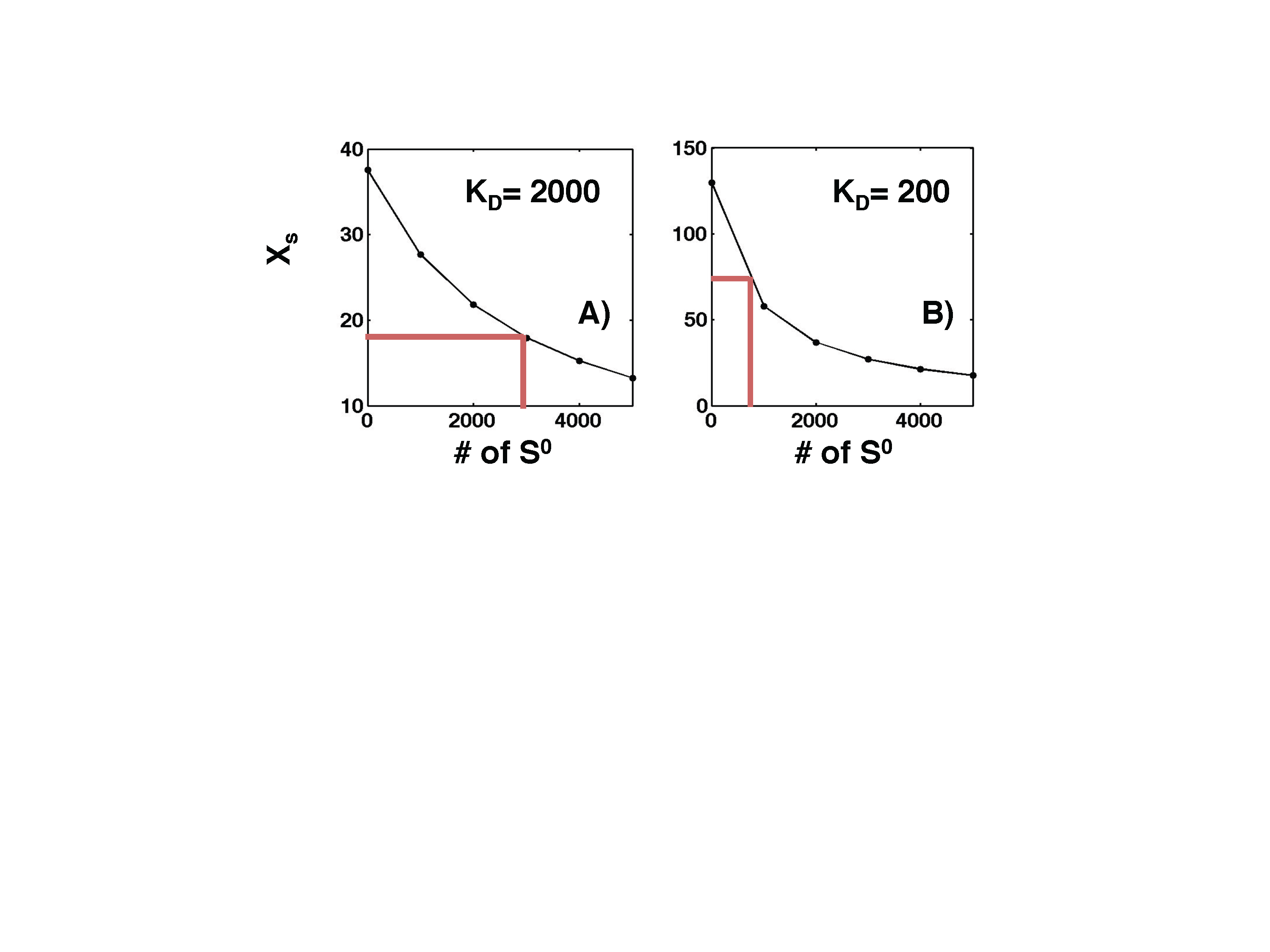

Supplement: Figure S10 — A large concentration of IP4 is required to replace PIP3 in models M5–M6. A) Variation of the steady state x s (Itk-PIP3) as a function of initial substrate (PIP2) concentration S 0 when the KD = 2000. B) Variation of the steady state x s as a function of initial substrate concentration S 0 when the KD = 200. (TIFF) [file pone.0073937.s010.tiff]

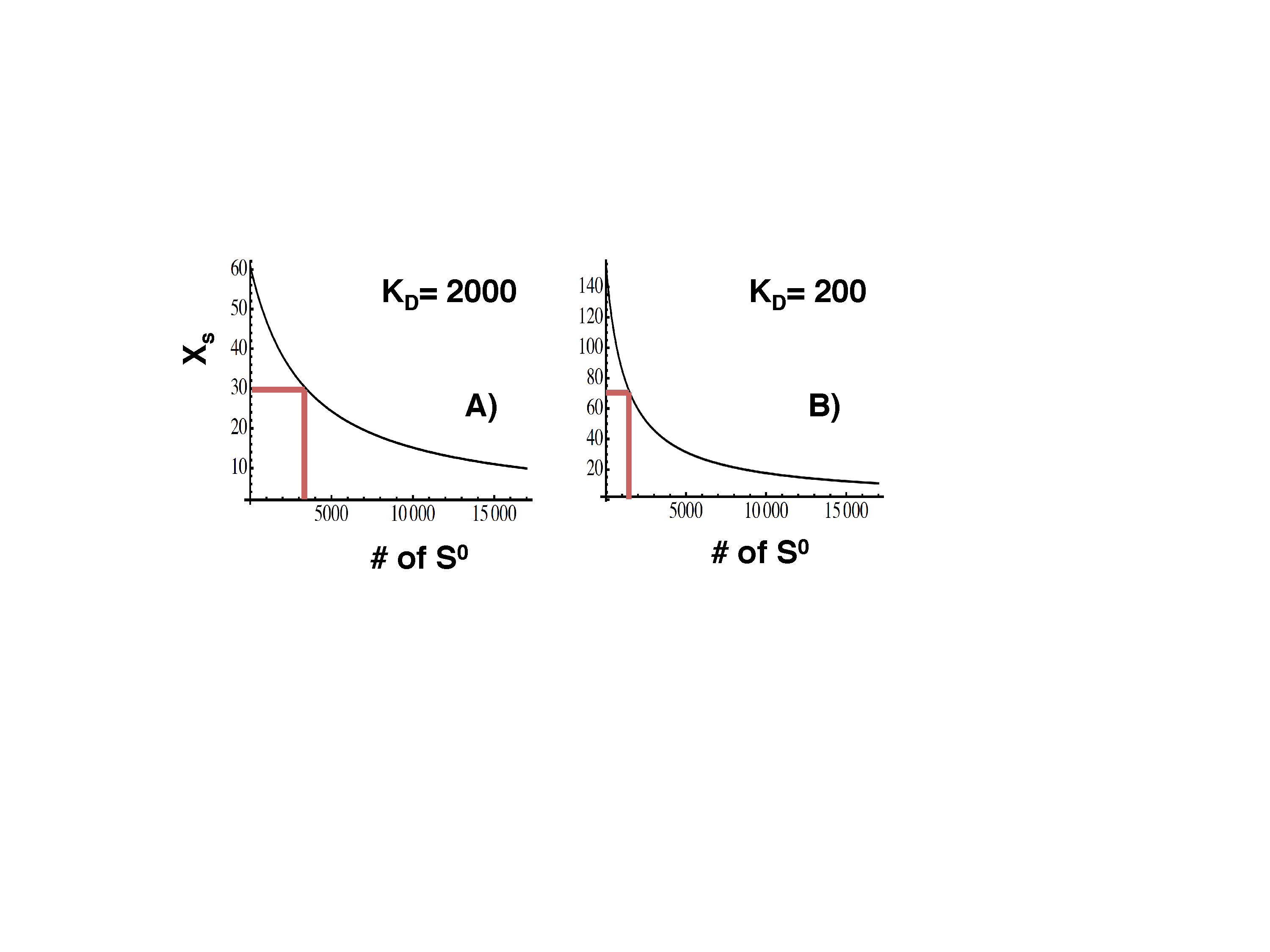

Supplement: Figure S11 — A large concentration of IP4 is required to replace PIP3 in model M4. A) Variation of the steady state xs (Itk-PIP3) as a function of initial substrate (PIP2) concentration S 0 when the KD = 2000. B) Same as in A) for KD = 200. (TIFF) [file pone.0073937.s011.tiff]

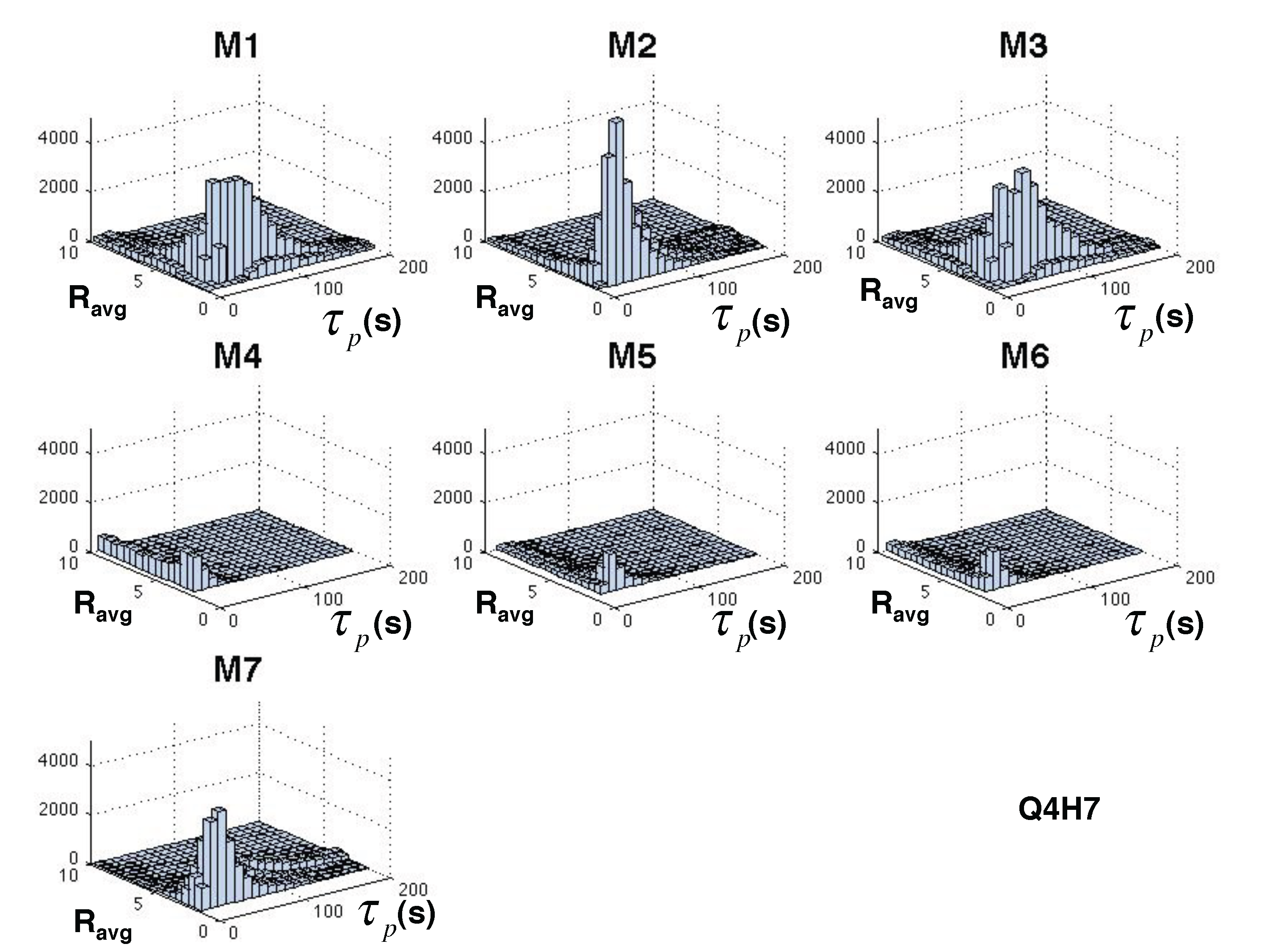

Supplement: Figure S12 — The histograms for R and τ as the parameters are varied in all 7 models for moderately low initial concentrations of Itk0 and PIP30. All the rate constants are varied by two orders of magnitude with the constraint KD low = α KD high. For M1–M3, α is distributed uniformly over 1 to 4000 while for M7 it is distributed uniformly over 1 to 50. The initial concentrations of species involved are varied in a 35% window about the base value of Itk0 = 40, PIP3 0 = 130 and PIP2 0 = 17000. (TIFF) [file pone.0073937.s012.tiff]

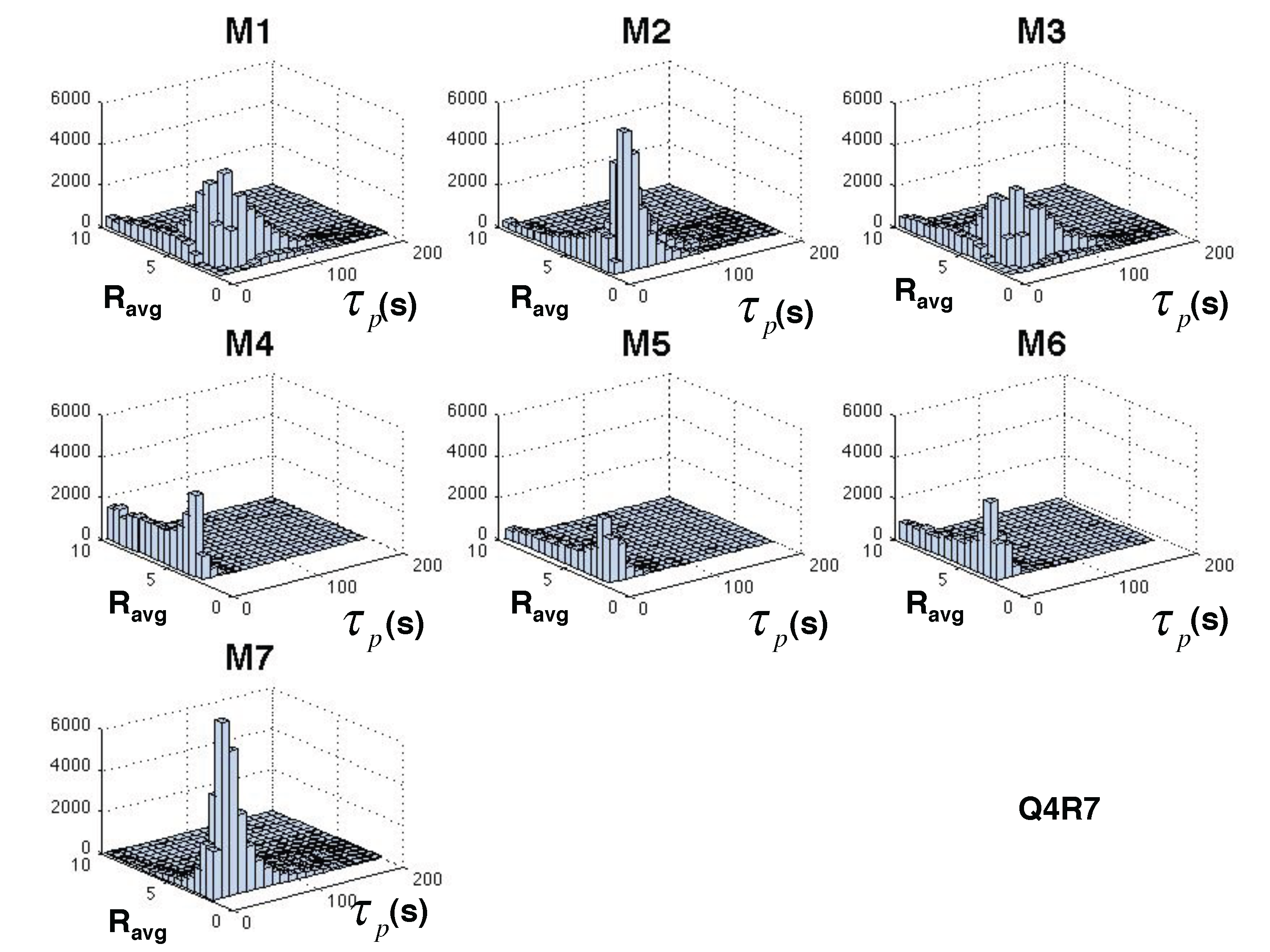

Supplement: Figure S13 — The histograms for R and τ as the parameters are varied in all 7 models for moderately high initial concentrations of Itk0 and PIP30. All the rate constants are varied by two orders of magnitude with the constraint KD low = α KD high. For M1–M3, α is distributed uniformly over 1 to 4000 while for M7 it is distributed uniformly over 1 to 50. The initial concentrations of species involved are varied in a 35% window about the base value of Itk0 = 100, PIP3 0 = 370 and PIP2 0 = 17000. (TIFF) [file pone.0073937.s013.tiff]

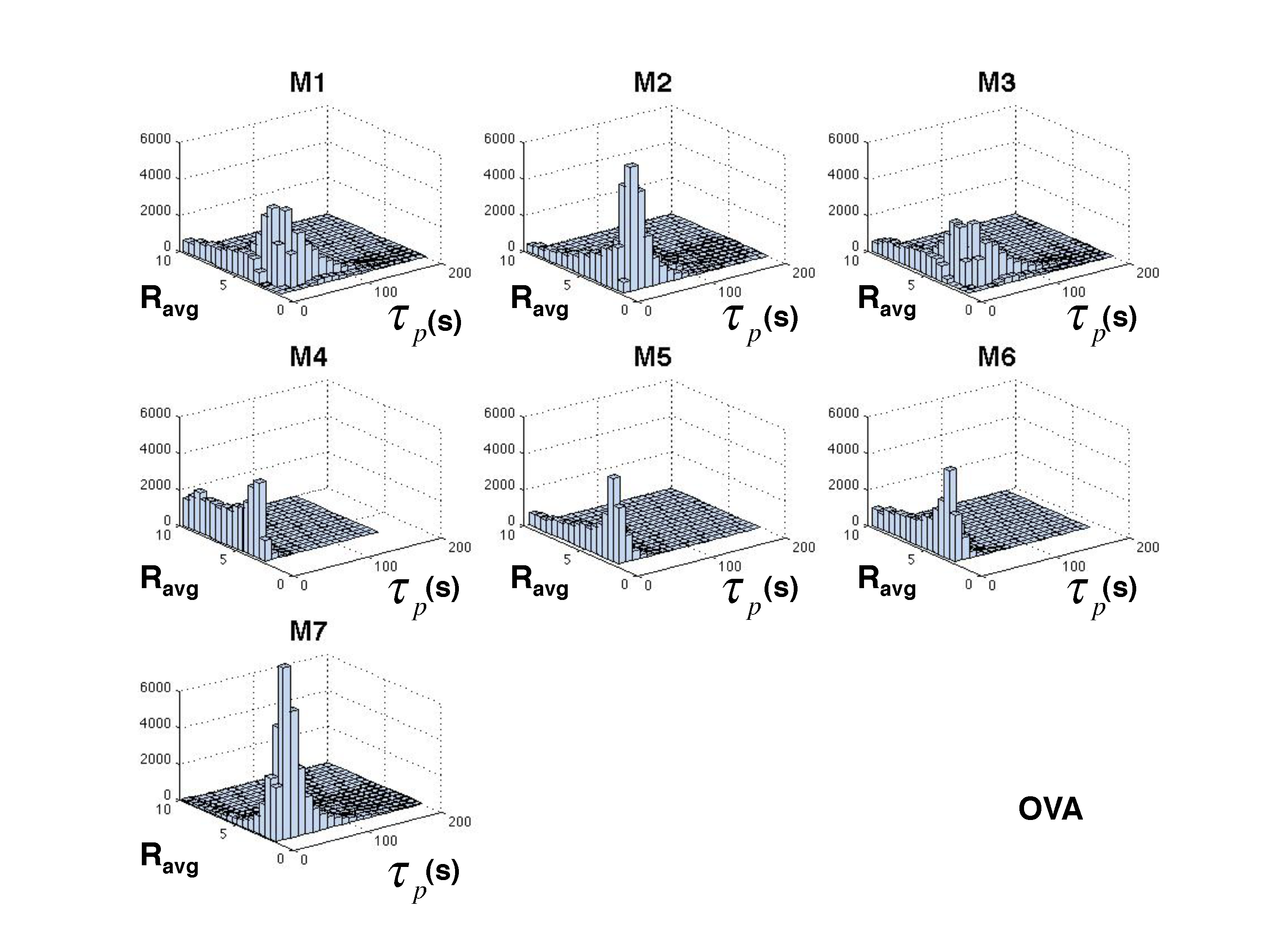

Supplement: Figure S14 — The histograms for R and τ as the parameters are varied in all 7 models for high initial concentrations of Itk0 and PIP30. All the rate constants are varied by two orders of magnitude with the constraint KD low = α KD high. For M1–M3, α is distributed uniformly over 1 to 4000 while for M7 it is distributed uniformly over 1 to 50. The initial concentrations of species involved are varied in a 35% window about the base value of Itk0 = 140, PIP3 0 = 530 and PIP2 0 = 17000. (TIFF) [file pone.0073937.s014.tiff]

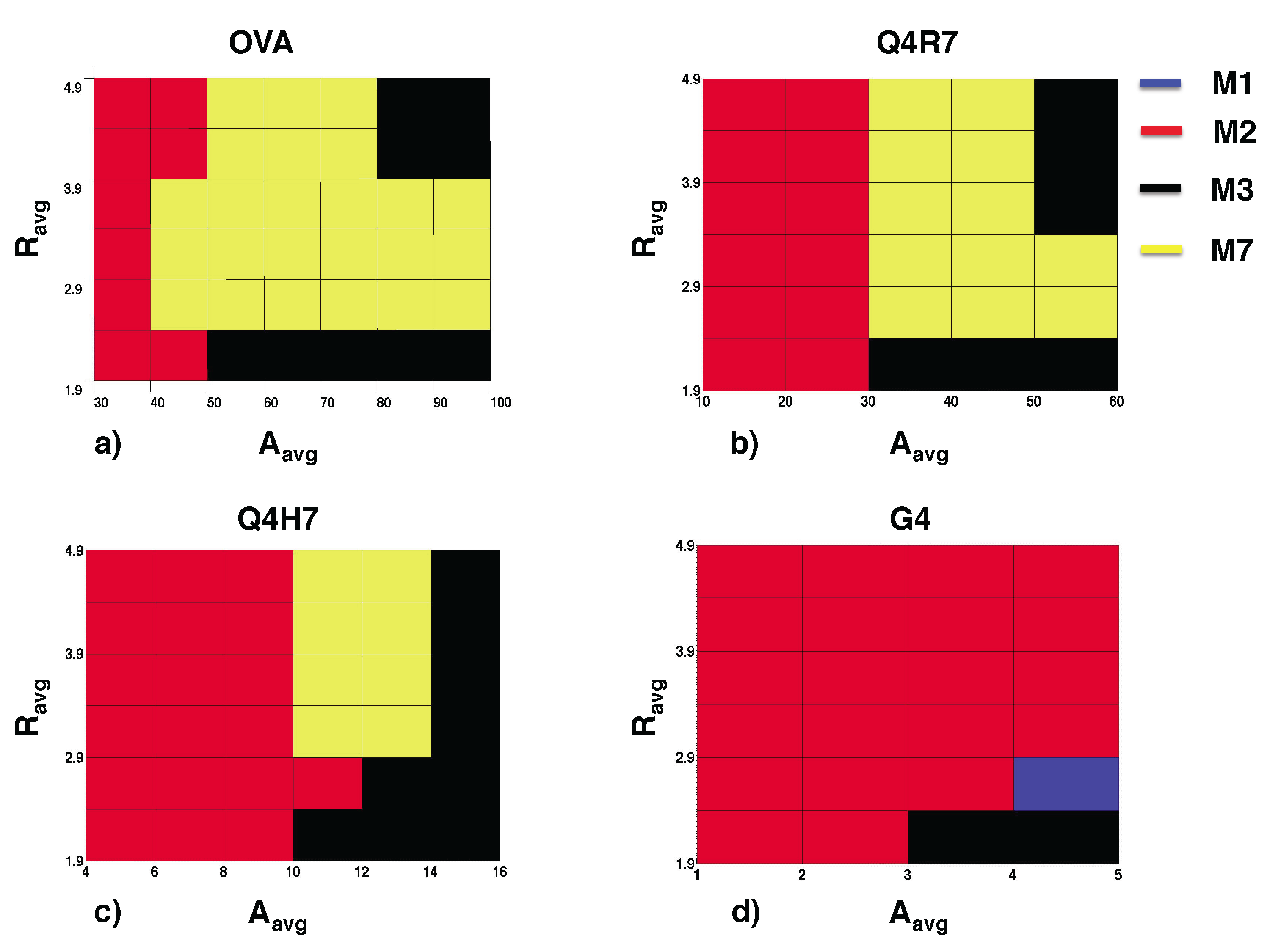

Supplement: Figure S15 — Checkerboard plot of the most robust models for different ligand affinities as Ravg and Aavg are varied for a fixed τavg = 2 mins. a) Plot of the most robust models for Itk0 = 140 and PIP3 0 = 530 molecules. b) The same plot as a) for Itk0 = 100 and PIP3 0 = 370 molecules. c) Same plot as a) for Itk0 = 40 and PIP3 0 = 130 molecules. d) The same plot as a) for Itk0 = 20 and PIP3 0 = 50 molecules. (TIFF) [file pone.0073937.s015.tiff]

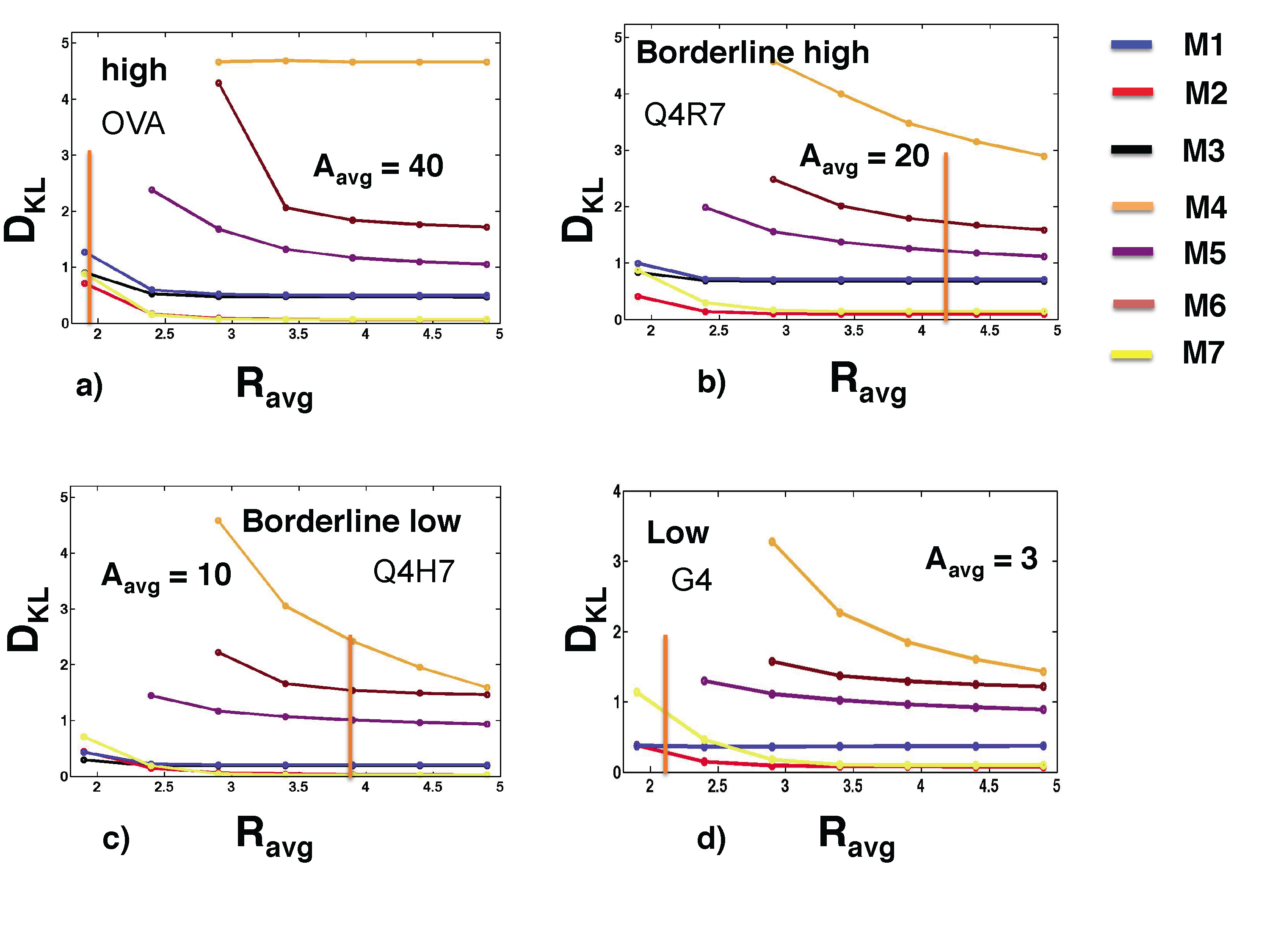

Supplement: Figure S16 — Plots of the relative robustness of all the 7 models for a specific Aavg for different ligand affinities as Ravg is varies for a fixed τavg = 2 mins. a) For Itk0 = 140 and PIP3 0 = 530 molecules the DKL is shown for an Aavg of 40 molecules. b) The same plot as a) for Itk0 = 100 and PIP3 0 = 370 molecules when the Aavg is held fixed at 20 molecules. c) Same plot as a) for Itk0 = 40 and PIP3 0 = 130 molecules when Aavg = 10 molecules. d) The same plot as a) for Itk0 = 20 and PIP3 0 = 50 molecules when Aavg = 3 moelcules. The orange vertical bar in all the plots show the experimentally observed value of Ravg. (TIFF) [file pone.0073937.s016.tiff]

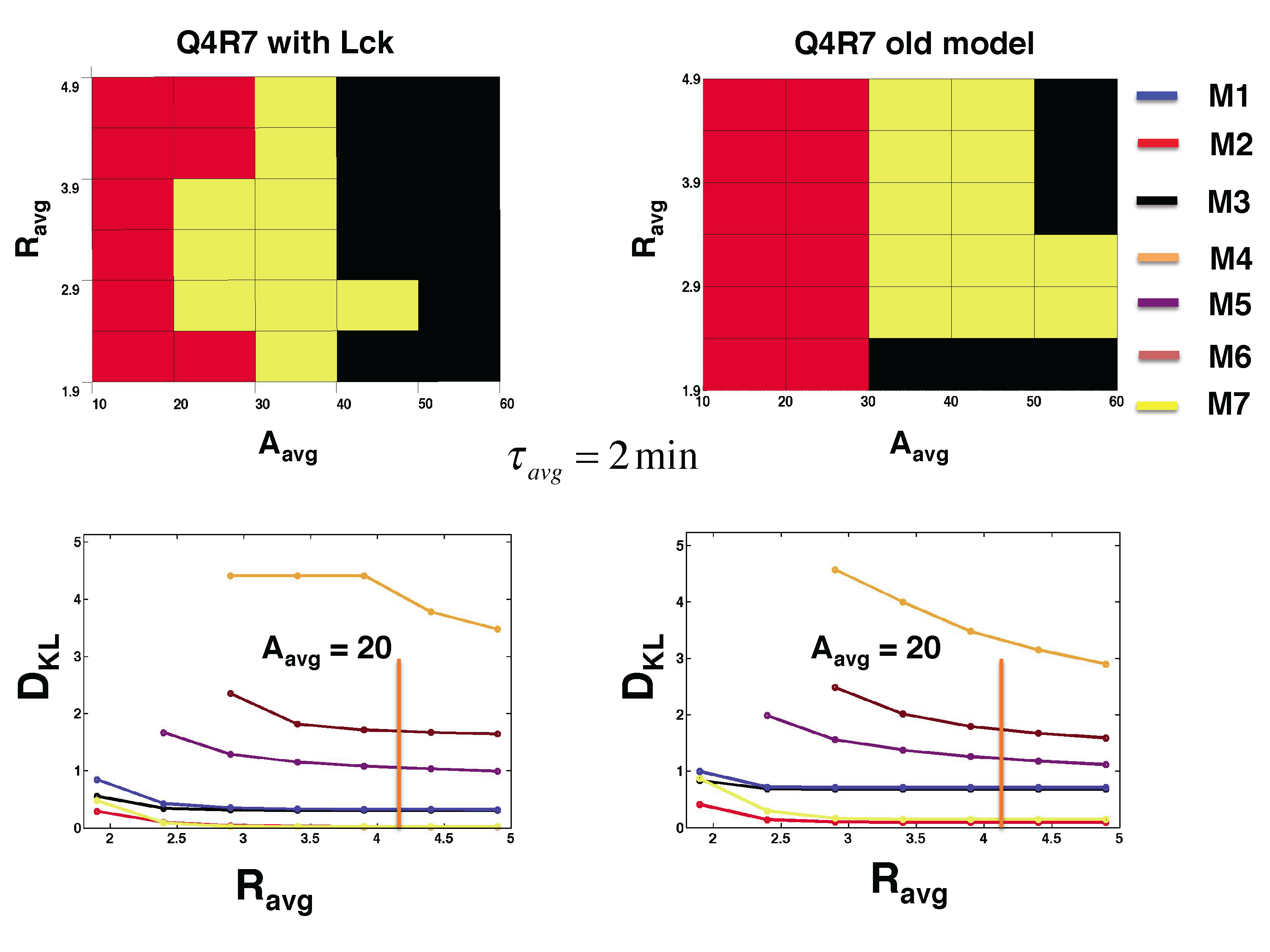

Supplement: Figure S17 — The effect of Lck mediated phosphorylation of Itk-PIP3 on the relative robustness of M1–M7. Upper panel (left most corner): For Itk0 = 100 and PIP3 0 = 370 the most robust models are shown as amplitude and the ratio of the Itk-PIP3 kinetics are varied in presence of the Lck mediated phosphorylation of membrane recruited Itk at its Y511 residue. The average peak time is held at 2 mins. Upper panel (right most corner): The same plot without any Lck mediated activation. Lower panel (left most corner): The relative robustness of the models M1–M7 for an amplitude average of 20 molecules in presence of Lck mediated activation of Itk. Lower panel (right most corner): Same plot without the explicit Lck mediated activation. (TIFF) [file pone.0073937.s017.tiff]

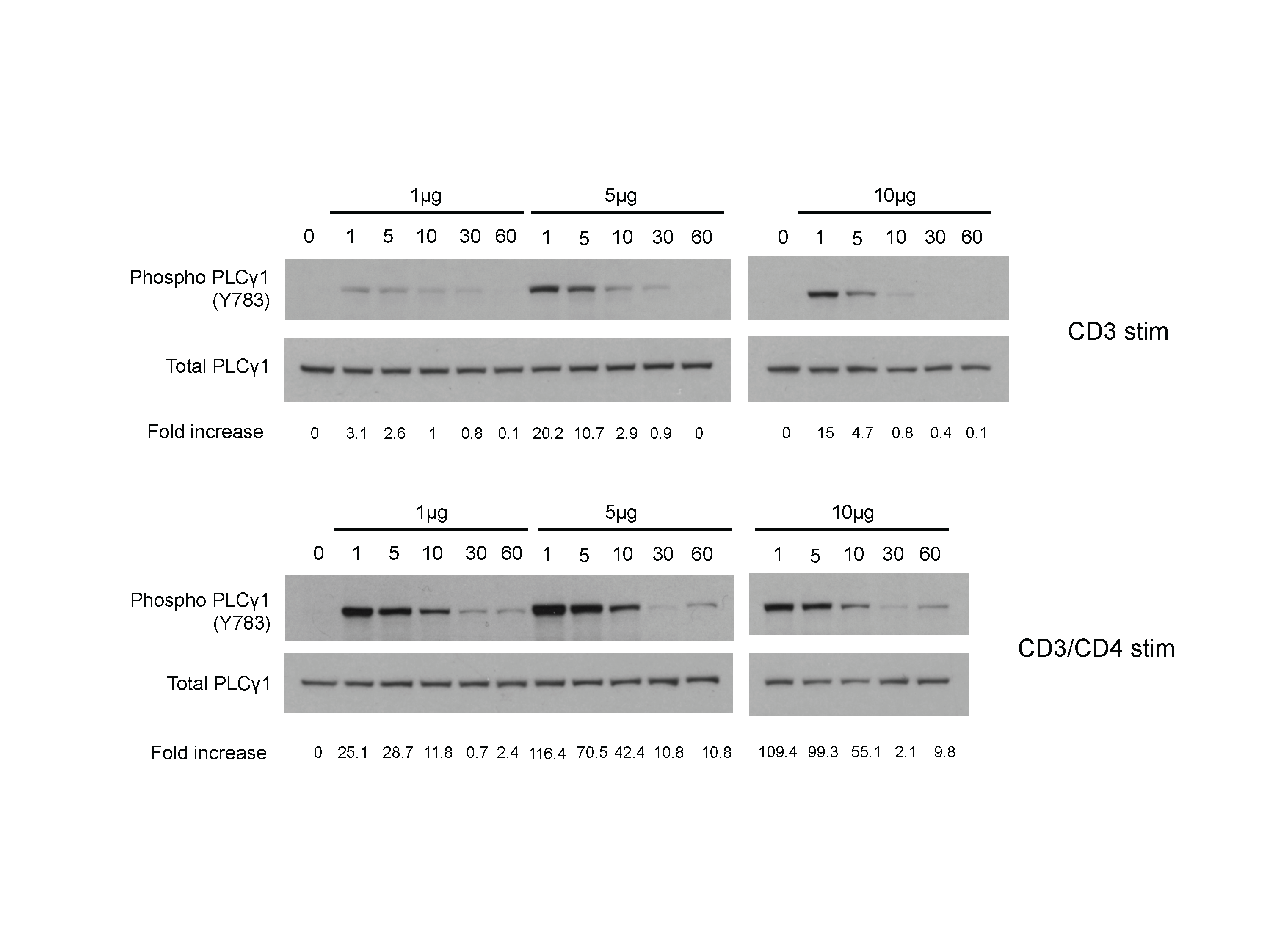

Supplement: Figure S18 — Kinetics of induction of PLCγ1 phosphorylation represented as the fold increase over non stimulated cells using total PLCγ1 protein as a loading control. (TIFF) [file pone.0073937.s018.tiff]

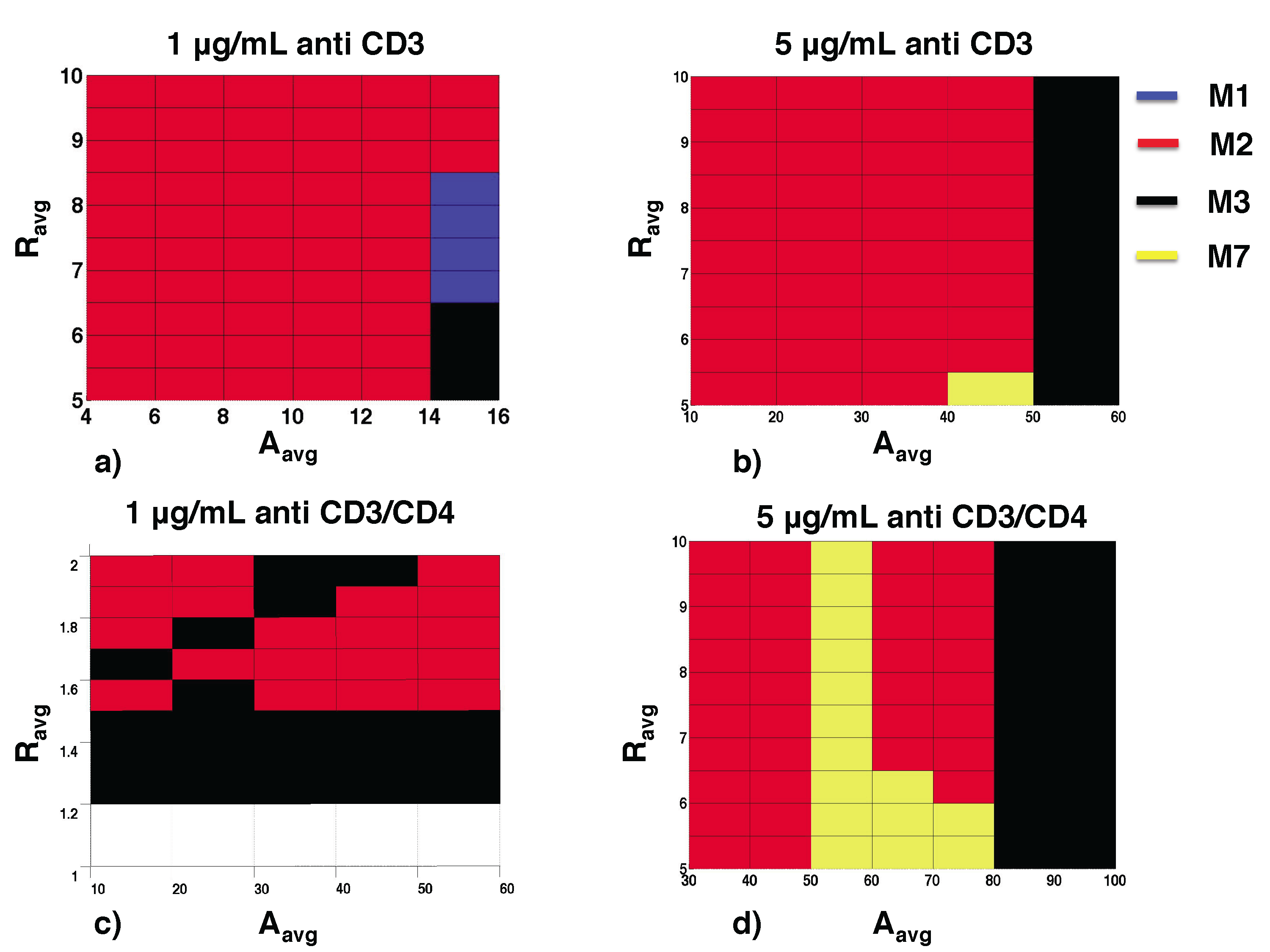

Supplement: Figure S19 — Checkerboard plot of the most robust models as Ravg and Aavg are varied for different doses of anti-CD3 and anti-CD3/CD4 antibodies. a) Itk0 = 40 and PIP3 0 = 130 molecules are used to emulate the 1 µg/mL anti CD3 stimulation. The τavg is held at 1 mins. The checkerboard diagram of the most robust models is shown as Ravg and Aavg are varied. b) Same as plot a) but Itk0 = 100 and PIP3 0 = 370 molecules are used as the initial concentrations. c) Itk0 = 100 and PIP3 0 = 370 molecules are used to emulate the 1 µg/mL anti CD3/CD4 stimulation. The τavg is held at 5 mins. The checkerboard diagram of the most robust models is shown as Ravg and Aavg are varied. d) Itk0 = 140 and PIP3 0 = 530 molecules are used to emulate the 5 µg/mL anti CD3/CD4 stimulation. The τavg is held at 1 mins. The checkerboard diagram of the most robust models is shown as Ravg and Aavg are varied. (TIFF) [file pone.0073937.s019.tiff]

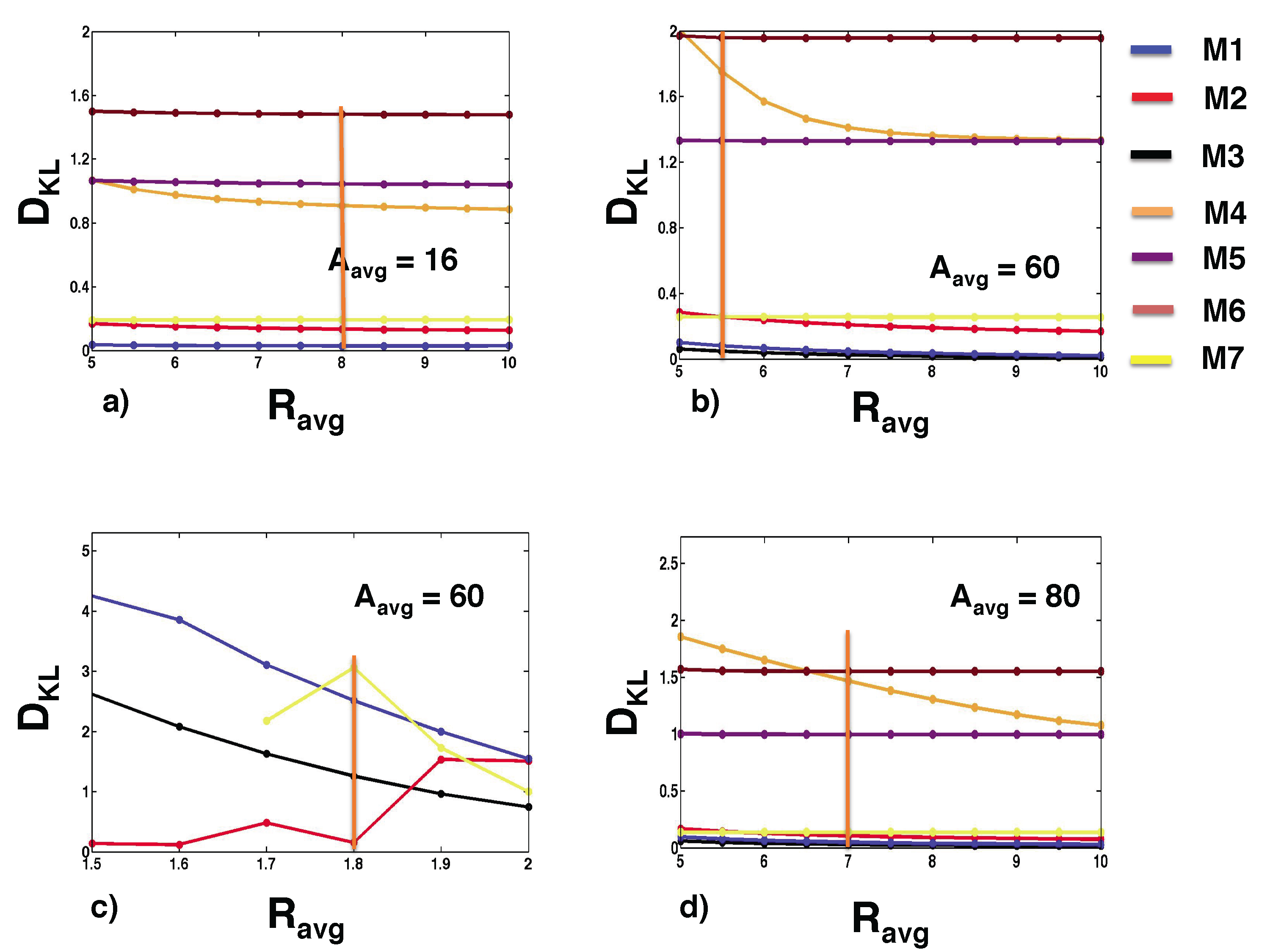

Supplement: Figure S20 — The plot of DKL for all the 7 models for a specific amplitude and different initial conditions for different doses of anti CD3 or anti CD3/CD4 antibodies. a) Itk0 = 40 and PIP3 0 = 130 molecules are used to emulate the 1 µg/mL anti CD3 stimulation. The τavg is held at 1 mins. The DKL is shown for an Aavg = 16 molecules. b) Same as plot a) but Itk0 = 100 and PIP3 0 = 370 molecules are used as the initial concentrations and Aavg = 60 molecules. c) Itk0 = 100 and PIP3 0 = 370 molecules are used to emulate the 1 µg/mL anti CD3/CD4 stimulation. The τavg is held at 5 mins. Aavg = 60 molecules. d) Itk0 = 140 and PIP3 0 = 530 molecules are used to emulate the 5 µg/mL anti CD3/CD4 stimulation. The τavg is held at 1 mins and Aavg is set equal to 80 molecules. The vertical orange bar shows the observed experimental values. (TIFF) [file pone.0073937.s020.tiff]

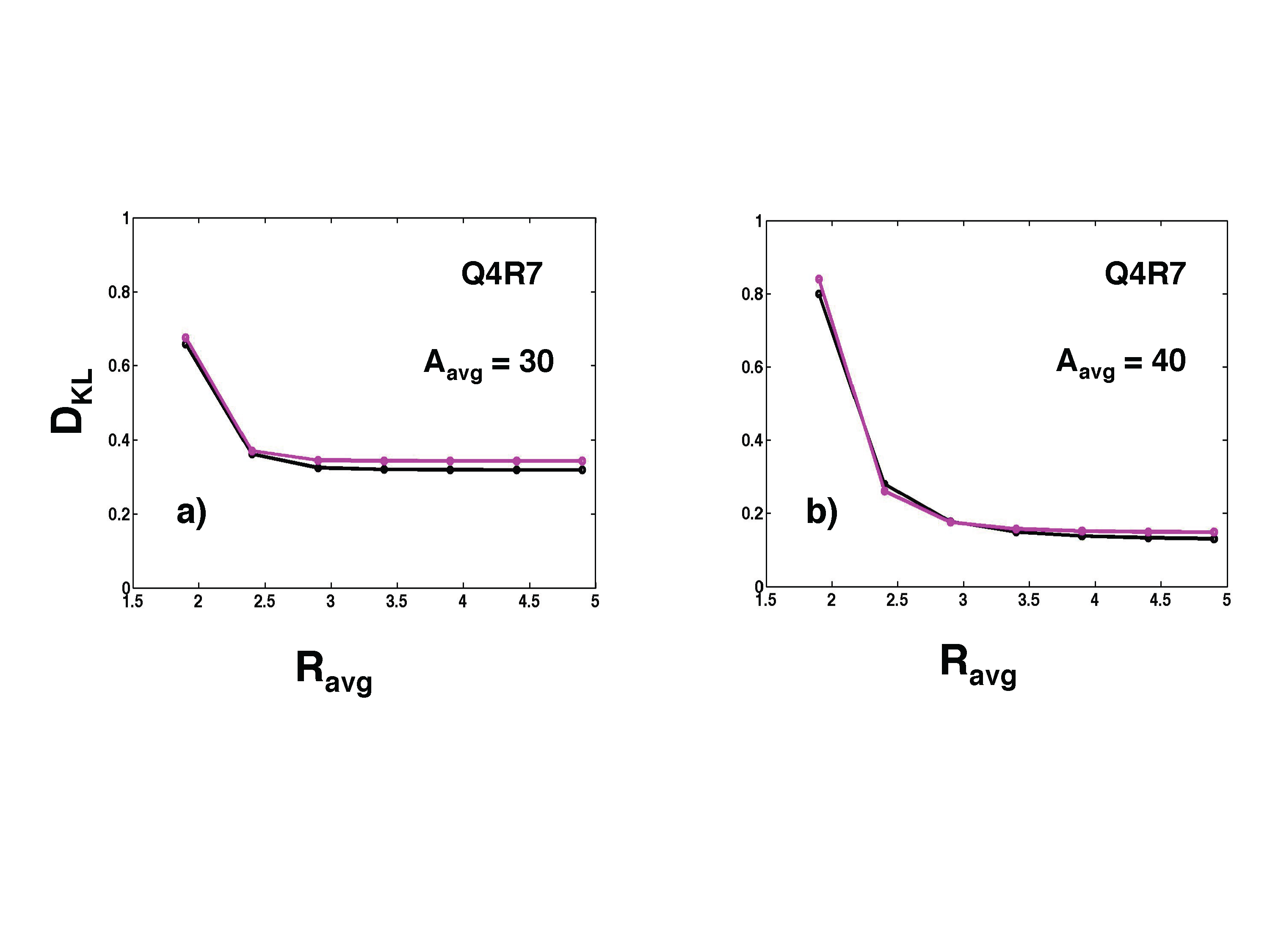

Supplement: Figure S21 — Addition of parameters which weakly affect the Itk-PIP3 kinetics, do not lead to any significant difference in the DKL. For Itk0 = 100 and PIP3 0 = 370, a) we have looked at the relative difference in the DKL of our old M3 (black) and M3 with the added reactions (magenta) for an amplitude average of 30 molecules and peak time average of 2 mins. b) We have looked at the relative difference in the DKL of our old M3 (black) and M3 with the added reactions (magenta) for an amplitude average of 40 molecules and peak time average of 2 mins. (TIFF) [file pone.0073937.s021.tiff]

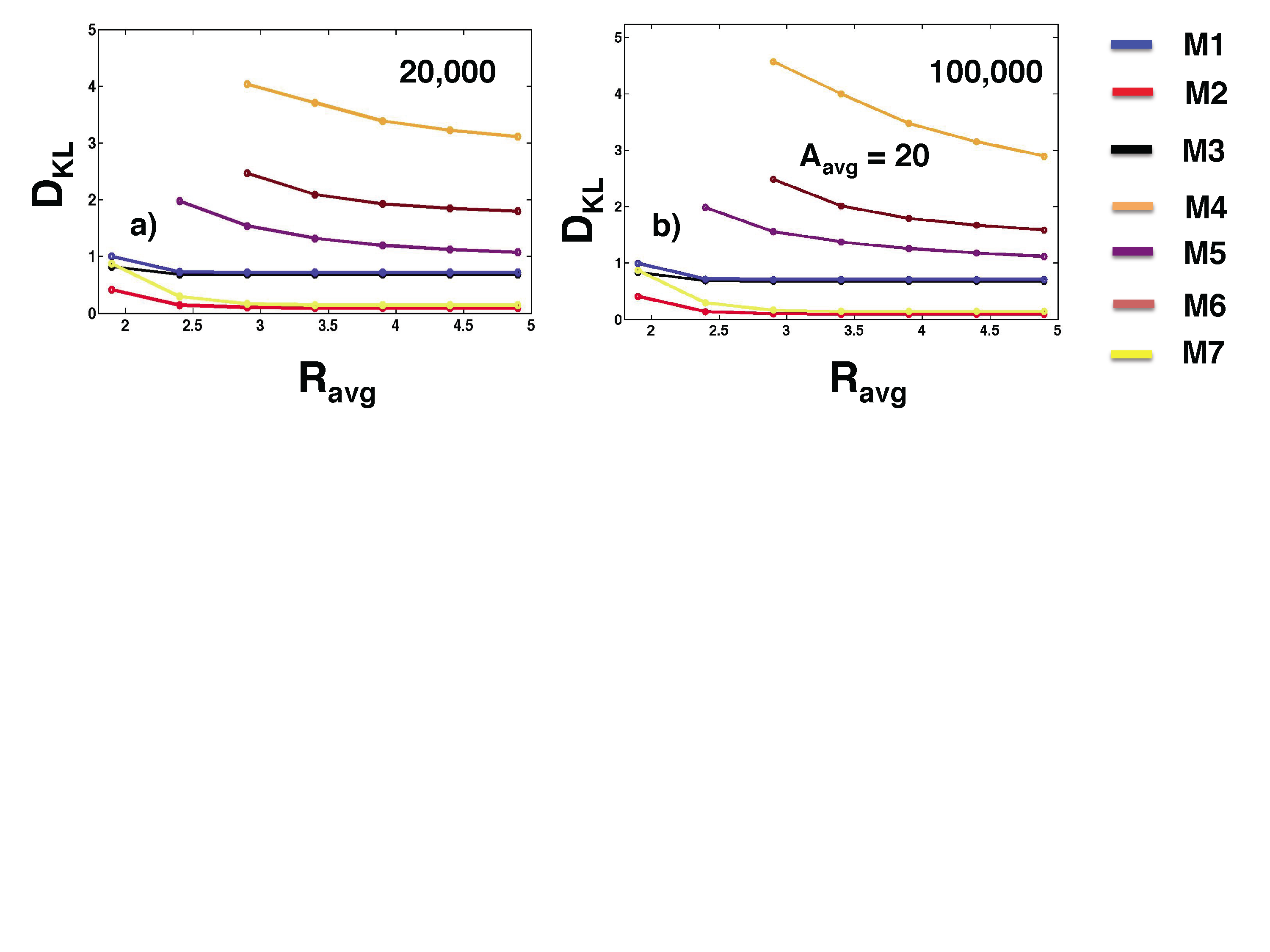

Supplement: Figure S22 — The sample set of 100,000 is a good sample size. We show the DKL of M1–M7 for Itk0 = 100 and PIP3 0 = 370 for a) 20,000 realizations and b) 100,000 realizations when the amplitude average is 20 molecules and the peak time average is 2 mins. The KL distances are identical. (TIFF) [file pone.0073937.s022.tiff]

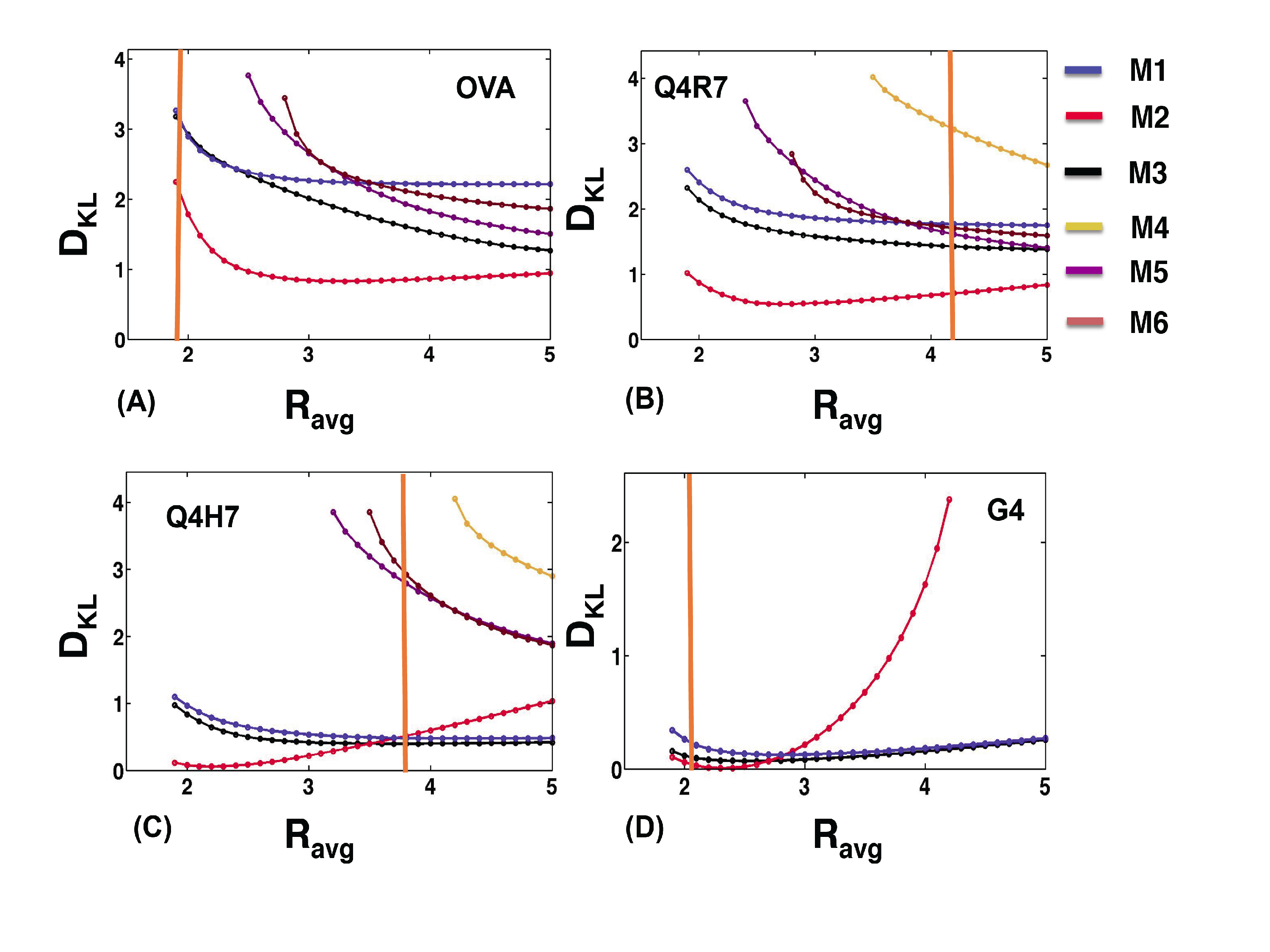

Supplement: Figure S23 — DKL without the constraint on amplitude. Lower DKL values (shown in log10 scale) denote higher robustness for any given Ravg. Based on the data in Fig. 4, the average peak time was fixed at 2 mins in all cases. Experimentally measured Ravg values are indicated by vertical orange lines. (A) Robustness for models M1–M3 and M5–M6 at high initial Itk (Itk0 = 140 molecules) and PIP3 concentrations (PIP30 = 530 molecules), simulating high-affinity OVA stimulation. M2 appears most robust in the experimentally observed Rave range. M4 fails produce any R value in the range investigated here. (B) M2 shows maximal robustness for moderate concentrations of initial Itk ( = 100 molecules) and PIP3 ( = 370 molecules), simulating Q4R7 stimulation. (C) For lower values of Itk0 ( = 40 molecules) and PIP30 ( = 130 molecules), simulating Q4H7 stimulation, M1–M3 are most robust with similar DKL values in the experimentally observed Rave range. (D) For low initial concentrations of Itk (Itk0 = 20 molecules) and PIP3 (PIP30 = 50 molecules), simulating stimulation by the low affinity peptide G4, M1–M3 are again most robust inthe experimentally observed Ravg range. Models M4–M6 fail to produce any value of R in the range investigated here. Model M7 is not shown. (TIFF) [file pone.0073937.s023.tiff]
