# Supplementary material for: In Silico Modeling of Itk Activation Kinetics in Thymocytes Suggests Competing Positive and Negative IP4 Mediated Feedbacks Increase Robustness
Source: PLoS One. 2013 Sep 16;8(9):e73937. doi: 10.1371/journal.pone.0073937 (PMC3774804; doi:10.1371/journal.pone.0073937)
Supplement: Table S1 — Reactions and rate constants for model M1. (DOCX) [file pone.0073937.s024.docx]

**Table S1: Reactions and rate constants for model M1.**

| **Reactions** | **k_on_** (μM^-1^s^-1^) | **k_off_**  (s^-1^) | **K_D_**  (μM) | **k_cat_**  (μM^-1^s^-1^) |
| --- | --- | --- | --- | --- |
|  | 2.5  10^-4^ | 0.1 | 400 * |  |
|  | 0.01 | 0.003 | 0.3 * |  |
|  | 2.5 10^-3^ | 0.1 | 40 * |  |
|  | 0.1 | 0.003 | 0.03 $\dagger$ ([2](#_ENREF_2)) |  |
|  | 0.01 | 0.003 | 0.3 $\dagger$ ([2](#_ENREF_2)) |  |
|  | 0.1 | 0.003 | 0.03 $\dagger$([2](#_ENREF_2)) |  |
|  |  |  |  | 1.5  10^-4^ ** |
|  |  |  |  | 1.5  10^-4^  ** |
|  |  |  |  | 1.5  10^-4^  ** |

$\dagger$ The high affinity binding of IP_4_ to the PH domain is taken to be in the nano-molar range based on the binding affinity of the isolated Btk PH domain for both IP_4_ and PIP_3_ (Ref. ([2](#_ENREF_2)) in Text S1). The binding affinity of the Itk PH domain for IP_4_ or PIP_3_ however is not known. The K_D_ for PIP_3_ binding to the isolated Btk PH domain is reported to be 7 times higher than the IP_4_ binding (Ref. ([2](#_ENREF_2)) in Text S1). For convenience we have taken it to be 10 times larger.

* Estimated. The low affinity binding of Itk PH domains to IP_4_/PIP_3_ has been assumed to be 1000 times weaker than the high affinity binding.

** Estimated. Hydrolysis of PIP_2_ and subsequent production of IP_3_, release of Ca^2+^ from the endoplasmic stores, Ca^2+^/Calmodulin dependent activation of ItpkB, and, finally the enzymatic turnover of IP_3_ by ItpkB have all been subsumed into the one single step where membrane bound Itk cleaves PIP_2_ to produce IP_4_. The rate constant for the above reaction is chosen to match the time scale of PLCγ1 activation reported in the experiments (Ref ([3](#_ENREF_3)) in Text S1). PIP_2_ is denoted as S for convenience.
