# Supplementary material for: In Silico Modeling of Itk Activation Kinetics in Thymocytes Suggests Competing Positive and Negative IP4 Mediated Feedbacks Increase Robustness
Source: PLoS One. 2013 Sep 16;8(9):e73937. doi: 10.1371/journal.pone.0073937 (PMC3774804; doi:10.1371/journal.pone.0073937)
Supplement: Table S3 — Reactions and rate constants for model M3. (DOCX) [file pone.0073937.s026.docx]

**Table S3: Reactions and rate constants for model M3.**

| **Reactions** | **k_on_**  (μM^-1^s^-1^) | **k_off_ (**s^-1^) | **K_D_** (μM) | **k_cat_**  (μM^-1^s^-1^) |
| --- | --- | --- | --- | --- |
|  | 2.5  10^-4^ | 0.1 | 400 |  |
|  | 2.5  10^-3^ | 0.1 | 40 |  |
|  | 0.1 | 0.003 | 0.03 |  |
|  | 0.01 | 0.003 | 0.3 |  |
|  | 0.1 | 0.003 | 0.03 |  |
|  |  |  |  | 1.5  10^-4^ |
|  |  |  |  | 1.5  10^-4^ |

All the high and low affinity binding of Itk PH domains with PIP_3_ and IP_4_, and, the IP_4_ production rate are taken to be the same as that shown for model M1 (Table S1).
