# Supplementary material for: In Silico Modeling of Itk Activation Kinetics in Thymocytes Suggests Competing Positive and Negative IP4 Mediated Feedbacks Increase Robustness
Source: PLoS One. 2013 Sep 16;8(9):e73937. doi: 10.1371/journal.pone.0073937 (PMC3774804; doi:10.1371/journal.pone.0073937)
Supplement: Table S5 — Reactions and rate constants for model M5. (DOCX) [file pone.0073937.s028.docx]

**Table S5: Reactions and rate constants for model M5.**

| **Reactions** | **k_on_** (μM^-1^s^-1^) | **k_off_** (s^-1^) | **K_D_** (μM) | **k**_cat_ (μM^-1^s^-1^) |
| --- | --- | --- | --- | --- |
|  | 1.25  10^-4^ | 0.05 | 400 |  |
|  | 1.25  10^-4^ | 0.05 | 400 |  |
|  | 1.25  10^-3^ | 0.05 | 40 |  |
|  | 1.25  10^-3^ | 0.05 | 40 |  |
|  | 1.25  10^-4^ | 0.05 | 400 |  |
|  | 1.25  10^-3^ | 0.05 | 40 |  |
|  |  |  |  | 1.5  10^-4^ |
|  |  |  |  | 1.5  10^-4^ |
|  |  |  |  | 1.5  10^-4^ |

All the low affinity binding of Itk PH domains with PIP_3_ and IP_4_, and, the IP_4_ production rate are taken to be the same as that shown for model M1 (Table S1).
