# Supplementary material for: In Silico Modeling of Itk Activation Kinetics in Thymocytes Suggests Competing Positive and Negative IP4 Mediated Feedbacks Increase Robustness
Source: PLoS One. 2013 Sep 16;8(9):e73937. doi: 10.1371/journal.pone.0073937 (PMC3774804; doi:10.1371/journal.pone.0073937)
Supplement: Table S7 — Reactions and rate constants for model M7. (DOCX) [file pone.0073937.s030.docx]

**Table S7: Reactions and rate constants for model M7.**

| **Reactions** | **k_on_**  (μM^-1^s^-1^) | **k_off_**  (s^-1^) | **K_D_**  (μM) | **k_cat_**  (μM^-1^s^-1^) |
| --- | --- | --- | --- | --- |
|  | 2.5  10^-4^ | 0.01 | 40 |  |
|  | 0.01 | 0.1 | 10 |  |
|  | 2.5  10^-3^ | 0.01 | 4 |  |
|  | 2.5  10^-3^ | 0.01 | 4 |  |
|  | 0.01 | 0.1 | 10 |  |
|  | 0.1 | 0.1 | 1 |  |
|  |  |  |  | 1.5  10^-4^ |
|  |  |  |  | 1.5  10^-4^ |
|  |  |  |  | 1.5  10^-4^ |

All the high and low affinity binding of Itk PH domains with PIP_3_ and IP_4_, and, the IP_4_ production rate are estimated to match the PLCγ1 kinetics in Ref. ([3](#_ENREF_3)) in Text S1.
