# Supplementary material for: In Silico Modeling of Itk Activation Kinetics in Thymocytes Suggests Competing Positive and Negative IP4 Mediated Feedbacks Increase Robustness
Source: PLoS One. 2013 Sep 16;8(9):e73937. doi: 10.1371/journal.pone.0073937 (PMC3774804; doi:10.1371/journal.pone.0073937)
Supplement: Table S8 — Values of the concentrations of different molecular species used in the models. (DOCX) [file pone.0073937.s031.docx]

**Table S8: Values of the concentrations of different molecular species used in the models.**

| **Molecules** | **Number** | **Comments** |
| --- | --- | --- |
| PIP_3_^0^ | Varied from 50-530 | Roughly 5% of the available PIP_2_ pool (Ref ([8](#_ENREF_8)) in Text S1) is taken to be the upper limit of PIP_3_ concentration. In a separate measurement, the PIP_3_ concentration reaches to about 150-200 μM in neutrophils, 10 seconds after stimulation (Ref ([6](#_ENREF_6)) in Text S1). |
| Itk-Itk^0^ (for dimers)/ Itk^0^(for monomers) | Varied from 20-300 | The upper limit of Itk is assumed to be the upper limit of phosphorylated LAT in thymocytes (Ref ([9](#_ENREF_9)) in Text S1). |
| S^0^ | 17000 | 3.5 mM, 10 seconds after stimulation in neutrophils (Ref ([6](#_ENREF_6)) in Text S1). |
| IP_4_ | We do not have any basal level of IP_4_ in the models. IP_4_ is generated via the cleavage of PIP_2_ (S). | The IP_4_ level in Jurkat T-lymphocytes increased to 1125 $\pm$ 125 pmol/10^9^ cells after stimulation by anti-CD3 antibody OKT3 (Ref ([7](#_ENREF_7)) in Text S1). This number, when converted to molecules/cell, is roughly two times the number we have used as an upper limit for IP_4_ (i.e. the initial PIP_2_ concentration) in our simulations. |
