# Supplementary material for: In Silico Modeling of Itk Activation Kinetics in Thymocytes Suggests Competing Positive and Negative IP4 Mediated Feedbacks Increase Robustness
Source: PLoS One. 2013 Sep 16;8(9):e73937. doi: 10.1371/journal.pone.0073937 (PMC3774804; doi:10.1371/journal.pone.0073937)
Supplement: Table S13 — Reactions and rate constants for model M5lck. (DOCX) [file pone.0073937.s036.docx]

**Table S13: Reactions and rate constants for model M5^lck^.**

| **Reactions** | **k_on_** (μM^-1^s^-1^) | **k_off_** (s^-1^) | **K_D_** (μM) | **k_cat_** (μM^-1^s^-1^) | **k_act_** (s^-1^) | **k_deact_**  (s^-1^) |
| --- | --- | --- | --- | --- | --- | --- |
|  | 1.25  10^-4^ | 0.05 | 400 |  |  |  |
|  | 1.25  10^-4^ | 0.05 | 400 |  |  |  |
|  | 1.25  10^-3^ | 0.05 | 40 |  |  |  |
|  | 1.25  10^-3^ | 0.05 | 40 |  |  |  |
|  | 1.25  10^-4^ | 0.05 | 400 |  |  |  |
|  | 1.25  10^-3^ | 0.05 | 40 |  |  |  |
| $IP_{4}-Itk-Itk^{act}-PIP_{3}+S\to IP_{4}+IP_{4}-Itk-Itk^{act}-PIP_{3}$ |  |  |  | 1.5  10^-4^ |  |  |
| $Itk-Itk^{act}-PIP_{3}+S\to IP_{4}+Itk-Itk^{act}-PIP_{3}$ |  |  |  | 1.5  10^-4^ |  |  |
| $PIP_{3}-Itk-Itk^{act}-PIP_{3}+S\to IP_{4}+PIP_{3}-Itk-Itk^{act}-PIP_{3}$ |  |  |  | 1.5  10^-4^ |  |  |
| $Itk-Itk-PIP_{3}\leftrightarrow Itk-Itk^{act}-PIP_{3}$ |  |  |  |  | 0.3 | 0.09 |
| $IP_{4}-Itk-Itk-PIP_{3}\leftrightarrow IP_{4}-Itk-Itk^{act}-PIP_{3}$ |  |  |  |  | 0.3 | 0.09 |
| $PIP_{3}-Itk-Itk-PIP_{3}\leftrightarrow PIP_{3}-Itk-Itk^{act}-PIP_{3}$ |  |  |  |  | 0.3 | 0.09 |
| $Itk-Itk^{act}-PIP_{3}\to Itk-Itk+PIP_{3}$ |  | 0.05 |  |  |  |  |
| $IP_{4}-Itk-Itk^{act}-PIP_{3}\leftrightarrow Itk-Itk^{act}-PIP_{3}+IP_{4}$ | 1.25  10^-3^ | 0.05 | 40 |  |  |  |
| $IP_{4}-Itk-Itk^{act}-PIP_{3}\leftrightarrow Itk-Itk-IP_{4}+PIP_{3}$ | 1.25  10^-4^ | 0.05 | 400 |  |  |  |
| $PIP_{3}-Itk-Itk^{act}-PIP_{3}\leftrightarrow Itk-Itk^{act}-PIP_{3}+PIP_{3}$ | 1.25  10^-4^ | 0.05 | 400 |  |  |  |
