# Supplementary material for: In Silico Modeling of Itk Activation Kinetics in Thymocytes Suggests Competing Positive and Negative IP4 Mediated Feedbacks Increase Robustness
Source: PLoS One. 2013 Sep 16;8(9):e73937. doi: 10.1371/journal.pone.0073937 (PMC3774804; doi:10.1371/journal.pone.0073937)
Supplement: Table S14 — Reactions and rate constants for model M6lck. (DOCX) [file pone.0073937.s037.docx]

**Table S14: Reactions and rate constants for model M6^lck^.**

| **Reactions** | **k_on_** (μM^-1^s^-1^) | **k_off_** (s^-1^) | **K_D_** (μM) | **k_cat_** (μM^-1^s^-1^) | **k_act_** (s^-1^) | **k_deact_** (s^-1^) |
| --- | --- | --- | --- | --- | --- | --- |
|  | 1.25  10^-4^ | 0.05 | 400 |  |  |  |
|  | 1.25  10^-3^ | 0.05 | 40 |  |  |  |
| $Itk-PIP_{3}\leftrightarrow Itk^{act}-PIP_{3}$ |  |  |  |  | 0.3 | 0.09 |
| $Itk^{act}-PIP_{3}\to Itk+PIP_{3}$ |  | 0.05 |  |  |  |  |
| $Itk^{act}-PIP_{3}+S\to IP_{4}+Itk^{act}-PIP_{3}$ |  |  |  | 1.5  10^-4^ |  |  |
