# Supplementary material for: In Silico Modeling of Itk Activation Kinetics in Thymocytes Suggests Competing Positive and Negative IP4 Mediated Feedbacks Increase Robustness
Source: PLoS One. 2013 Sep 16;8(9):e73937. doi: 10.1371/journal.pone.0073937 (PMC3774804; doi:10.1371/journal.pone.0073937)
Supplement: Table S16 — Values of peak time, peak width, and asymmetry ratio R calculated from the PLCγ1 activation kinetics in Figure S18. (DOCX) [file pone.0073937.s039.docx]

**Table S16: Values of peak time, peak width, and asymmetry ratio R calculated from the PLCγ1 activation kinetics in Figure. S18**

| **CD3 μg / mL** | **   τ_p_) (mins.)** | **  (τ_w_) (mins.)** | **R** |
| --- | --- | --- | --- |
| 1 | 1.0 | 8.0 | 8.0 |
| 5 | 1.0 | 5.5 | 5.5 |

| **CD3 & CD4 μg / mL** | **   τ_p_) (mins.)** | **  (τ_w_) (mins.)** | **R** |
| --- | --- | --- | --- |
| 1 | 5.0 | 9.0 | 1.8 |
| 5 | 1.0 | 7.0 | 7.0 |
