# Supplementary material for: In Silico Modeling of Itk Activation Kinetics in Thymocytes Suggests Competing Positive and Negative IP4 Mediated Feedbacks Increase Robustness
Source: PLoS One. 2013 Sep 16;8(9):e73937. doi: 10.1371/journal.pone.0073937 (PMC3774804; doi:10.1371/journal.pone.0073937)
Supplement: Table S17 — New reactions added to M3. (DOCX) [file pone.0073937.s040.docx]

**Table S17: New reactions added to M3**

| **Reactions** | **k_forward_**) | **k_back_ (s^-1^)** | **k_cat_** |
| --- | --- | --- | --- |
| $Itk-PIP_{3}+PLC\gamma\to Itk-PIP_{3}+PLC\gamma^{*}$ | 5.0 μM^-1^s^-1^ |  |  |
| $IP_{4}-Itk-PIP_{3}+PLC\gamma\to IP_{4}-Itk-PIP_{3}+PLC\gamma^{*}$ | 5.0 μM^-1^s^-1^ |  |  |
| $PLC\gamma^{*}\to PLC\gamma$ |  | 8.0 s^-1^ |  |
| $PLC\gamma^{*}+S\to IP_{3}+PLC\gamma^{*}$ |  |  | 1.5  10^-4^ μM^-1^s^-1^ |
| $IP_{3}\to IP_{4}$ |  |  | 0.7 s^-1^ |
