# Supplementary material for: In Silico Modeling of Itk Activation Kinetics in Thymocytes Suggests Competing Positive and Negative IP4 Mediated Feedbacks Increase Robustness
Source: PLoS One. 2013 Sep 16;8(9):e73937. doi: 10.1371/journal.pone.0073937 (PMC3774804; doi:10.1371/journal.pone.0073937)
Supplement: Text S1 — Supporting calculations and discussions. (DOCX) [file pone.0073937.s041.docx]

**Details of the models and simulations:**

We used a set of ODEs to describe the signaling kinetics of concentrations of proteins and lipids in the system. The descriptions of the kinetics using ODEs neglect stochastic fluctuations arising from intrinsic noise fluctuations and assume a spatially well-mixed system. This is a good approximation when diffusion time scales are fast compared to the reaction time scales. Since the time scales for diffusion depend on the spatial extent of the system, the system can be assumed to be spatially homogeneous in a region of volume V (Figure S1A), which is small compared the to the total volume of a T cell. In addition, the signaling reactions in our system can take place between a pair of molecules, where both species reside in the plasma membrane. For these reactions, we convert the values of binding rates given in three dimensions to approximate two dimensional rates. We provide details regarding the above approximations below. The reactions are shown in Tables S1-S7, and the corresponding ODEs are shown below the tables. The model reactions are also shown graphically in Figure S1B. The software package, BIONETGEN, was used to construct and solve the ODEs. The BIONETGEN codes are available at <http://planetx.nationwidechildrens.org/~jayajit/>.

The simulation box (Figure S1A) is divided into two compartments, plasma membrane and cytosol. The molecules in the cytosol can react with the plasma membrane bound molecules only when they are in a close proximity (~*l*=2 nm, Fig. S1). This length scale is used to convert three dimensional reaction rates to two dimensional reaction rates describing reactions in the plasma membrane. The area of the plasma membrane is taken to be 4 μm^2^ (LxL in Fig. S1). The volume of the cytosol is 0.08 μm^3^ (LxLxd in Figure S1) and the volume of the plasma membrane compartment is 0.008 μm^3^ (LxLx0.002 μm). All the molecular species were distributed homogenously across the simulation box. This is a good assumption when the time scales of diffusion of the molecules are much faster compared to the fastest reaction time scale. Since the fastest reaction time scale (~10 s, corresponding to unbinding rate of Itk – PIP_3_, see the tables below) is comparable to the slowest diffusion time scales (D ~ 0.1 μm^2^/s for membrane bound species ([1](#_ENREF_1))) on the length scale (2 μm) of the simulation box, we assumed the plasma membrane bound species to be homogeneously distributed in space. The cytosolic molecules diffuse with a much faster time scale (D ~ 10 μm^2^/s ([1](#_ENREF_1))) compared to the plasma membrane bound molecules, therefore, we also assumed that those molecules are also homogeneously distributed in the simulation box.

ODEs for Model 1: The reactions for Model 1 are given in the Table S1. The rules for construction of the ODEs are discussed below.

*Construction of the ODEs describing the signaling kinetics.*

The molecular species*,* Itk-Itk (we denote this species by Itk_D_), representing Itk-Itk dimers bound to the TCR and LAT signalosome, resides at the interface of the plasma membrane and the cytosol. The lipid PIP_3_, and the Itk-Itk-PIP_3_ complexes (Itk-Itk-PIP_3_, IP_4_-Itk-Itk-PIP_3_), and PIP_2_ (denoted as S) also reside in the plasma membrane. The molecules IP_4_ and IP_4_-Itk-Itk-IP_4_ are soluble and dwell in the cytosol. We use two well-mixed compartments representing plasma membrane and cytosol (Figure S1A) and use ODEs to describe the kinetics for the molecules in those compartments. E.g., concentration of Itk_D_ or [Itk_D_] at a time t is given by,

$[Itk_{D}](t)=\frac{N_{Itk_{D}}(t)}{Al}\theta(z+l)$ , where, $N_{Itk_{D}}(t)$ denotes the total number of Itk dimers in the system, and $\theta(z)$ is the Heaveside step function defined as, $\theta(z)=0$ for $z<0$, and, $\theta(z)=1$for $z\geq0$. In the simulation box, z ranges from z=0 to z=-d. The region between $0\geq z\geq-l$ represents the plasma membrane and $-l\geq z\geq-d$ represents the cytosolic part in the simulation. All the plasma membrane bound species concentrations are defined as above. The concentration of cytosolic IP_4_ is given by, $[IP_{4}](t)=\frac{N_{IP_{4}}(t)}{A(d-l)}\theta(z+d)\theta(z-l)\approx\frac{N_{IP_{4}}(t)}{Ad}\theta(z+d)\theta(z-l)$. The last approximation follows as $l(=2nm)\ll d(=0.02\mu m)$. Concentrations of all the cytosolic species are defined in the same way. Using the scheme described above we write down the ODEs describing kinetics for the total concentrations of the species present in the model. Below we show how the ODEs are constructed from the reactions using an example.

Consider the following reaction

, where *K_on_* and *K*_off_ are the binding and the unbinding rates respectively. Using the law of mass action the time evolution of the Itk-Itk-IP_4_ complex can be written as

$\frac{d[Itk-Itk-IP_{4}]}{dt}=K_{on}[Itk-Itk][IP_{4}]-K_{off}[Itk-Itk-IP_{4}]$.

The species Itk-Itk-IP_4_ and Itk-Itk reside in the plasma membrane whereas IP_4_ resides in the cytosol. Therefore, as described above, , , and [IP_4_](t) is related to N_IP4_ as described previously.

We denote,V_membrane_=*Al* and V_cytosol_=*Ad*. Multiplying the above rate equation by V_membrane_, we get,

$\frac{dN_{Itk-Itk-IP_{4}}}{dt}=\frac{K_{on}}{V_{cytosol}}N_{Itk-Itk}N_{IP_{4}}-K_{off}N_{Itk-Itk-IP_{4}}$. Note that the rate, *K_on_* , for a binding reaction between a plasma membrane bound molecule and a cytosolic molecule, is scaled by the volume of the cytosol whereas the *K_off_* remains the same. For a reaction like , where all the molecular species reside in the plasma membrane we find,

$\frac{dN_{Itk-Itk-PIP_{3}}}{dt}=\frac{K_{on}}{V_{membrane}}N_{Itk-Itk}N_{PIP_{3}}-K_{off}N_{Itk-Itk-PIP_{3}}$.

The on rate, K_on_, is scaled by the volume of the membrane V_membrane_  instead of the V_cytosol_= *Ad*. Following the above procedure we write the ODEs for the model M1. For convenience we denote the Itk-Itk dimer as Itk_D_. We do not show the volume scaling factors explicitly, all the rates shown in the ODEs have the unit of [time]^-1^. These rates are calculated using the values given in Table S1 and then scaling those values with appropriate volume factors.

There are three conservation laws, namely

$$\begin{matrix} N_{Itk_{D}}+N_{Itk_{D}-PIP_{3}}+N_{PIP_{3}-Itk_{D}-PIP_{3}}+N_{IP_{4}-Itk_{D}-PIP_{3}}+N_{IP_{4}-Itk_{D}-IP_{4}}=N_{Itk_{D}}(t=0) \\ N_{PIP_{3}}+N_{Itk_{D}-PIP_{3}}+N_{IP_{4}-Itk_{D}-PIP_{3}}+2*N_{PIP_{3}-Itk_{D}-PIP_{3}}=N_{PIP_{3}}(t=0) \\ N_{IP_{4}}+N_{Itk_{D}-IP_{4}}+N_{IP_{4}-Itk_{D}-PIP_{3}}+2*N_{IP_{4}-Itk_{D}-IP_{4}}+N_{S}=N_{S}(t=0) \end{matrix}$$

the equations above give the time evolution of all the proteins and the protein complexes.

ODEs for Model 2: The reactions for Model 2 are given in Table S2. The corresponding ODEs are

The conservation laws are,

$$\begin{matrix} N_{Itk_{D}}+N_{Itk_{D}-PIP_{3}}+N_{IP_{4}-Itk_{D}-PIP_{3}}+N_{IP_{4}-Itk_{D}-IP_{4}}=N_{Itk_{D}}(t=0) \\ N_{PIP_{3}}+N_{Itk_{D}-PIP_{3}}+N_{IP_{4}-Itk_{D}-PIP_{3}}=N_{PIP_{3}}(t=0) \\ N_{IP_{4}}+N_{Itk_{D}-IP_{4}}+N_{IP_{4}-Itk_{D}-PIP_{3}}+2*N_{IP_{4}-Itk_{D}-IP_{4}}+N_{S}=N_{S}(t=0) \end{matrix}$$

ODEs for Model 3: The reactions for Model 3 are given in the Table S3. The corresponding ODEs and the conservation laws are same as Model2.

ODEs for Model 4: The reactions for Model 4 are given in the Table S4. The corresponding ODEs are

$$\begin{matrix} \frac{dN_{Itk}}{dt}=k_{-1}N_{Itk-PIP_{3}}-k_{1}N_{Itk}N_{PIP_{3}}+k_{-2}N_{Itk^{*}-IP_{4}}-k_{2}N_{Itk}N_{IP_{4}} \\ \frac{dN_{Itk-PIP_{3}}}{dt}=k_{1}N_{Itk}N_{PIP_{3}}-k_{-1}N_{Itk-PIP_{3}} \\ \frac{dN_{Itk^{*}-IP_{4}}}{dt}=k_{2}N_{Itk}N_{IP_{4}}-k_{-2}N_{Itk^{*}-IP_{4}}+k_{3}N_{Itk^{*}-PIP_{3}}N_{IP_{4}}-k_{3}N_{Itk^{*}-IP_{4}}N_{PIP_{3}} \\ \frac{dN_{Itk^{*}-PIP_{3}}}{dt}=k_{3}N_{Itk^{*}-IP_{4}}N_{PIP_{3}}-k_{3}N_{Itk^{*}-PIP_{3}}N_{IP_{4}} \\ \frac{dN_{PIP_{3}}}{dt}=k_{-1}N_{Itk-PIP_{3}}-k_{1}N_{Itk}N_{PIP_{3}}+k_{3}N_{Itk^{*}-PIP_{3}}N_{IP_{4}}-k_{3}N_{Itk^{*}-IP_{4}}N_{PIP_{3}} \\ \frac{dN_{IP_{4}}}{dt}=k_{-2}N_{Itk^{*}-IP_{4}}-k_{2}N_{Itk}N_{IP_{4}}+k_{3}N_{Itk^{*}-IP_{4}}N_{PIP_{3}}-k_{3}N_{Itk^{*}-PIP_{3}}N_{IP_{4}} \\ \text{ }+k_{4}\left\{N_{Itk-PIP_{3}}+N_{Itk^{*}-PIP_{3}}\right\}N_{S} \\ \frac{dN_{S}}{dt}=-k_{4}\left\{N_{Itk-PIP_{3}}+N_{Itk^{*}-PIP_{3}}\right\}N_{S} \end{matrix}$$

Here we have used Itk not Itk_D_ to denote the monomeric Itk molecules. The conservation laws are,

$$\begin{matrix} N_{Itk}+N_{Itk-PIP_{3}}+N_{Itk^{*}-IP_{4}}+N_{Itk^{*}-PIP_{3}}=N_{Itk}(t=0) \\ N_{PIP_{3}}+N_{Itk^{*}-PIP_{3}}+N_{Itk-PIP_{3}}=N_{PIP_{3}}(t=0) \\ N_{IP_{4}}+N_{Itk^{*}-IP_{4}}+N_{S}=N_{S}(t=0) \end{matrix}$$

ODEs for Model 5: The reactions for Model 5 are given in the Table S5. The ODEs are the same as model 1 but the rate constants are different.

ODEs for Model 6: The reactions for Model 6 are given in the Table S6. The ODEs are

$$\begin{matrix} \frac{dN_{Itk}}{dt}=k_{-1}N_{Itk-PIP_{3}}-k_{1}N_{Itk}N_{PIP_{3}}+k_{-2}N_{Itk-IP_{4}}-k_{2}N_{Itk}N_{IP_{4}} \\ \frac{dN_{Itk-PIP_{3}}}{dt}=k_{1}N_{Itk}N_{PIP_{3}}-k_{-1}N_{Itk-PIP_{3}} \\ \frac{dN_{Itk-IP_{4}}}{dt}=k_{2}N_{Itk}N_{IP_{4}}-k_{-2}N_{Itk-IP_{4}} \\ \frac{dN_{PIP_{3}}}{dt}=k_{-1}N_{Itk-PIP_{3}}-k_{1}N_{Itk}N_{PIP_{3}} \\ \frac{dN_{IP_{4}}}{dt}=k_{-2}N_{Itk-IP_{4}}-k_{2}N_{Itk}N_{IP_{4}}+k_{4}N_{Itk-PIP_{3}}N_{S} \\ \frac{dN_{S}}{dt}=-k_{4}N_{Itk-PIP_{3}}N_{S} \end{matrix}$$

The conservation laws are

$$\begin{matrix} N_{Itk}+N_{Itk-PIP_{3}}+N_{Itk-IP_{4}}=N_{Itk}(t=0) \\ N_{PIP_{3}}+N_{Itk-PIP_{3}}=N_{PIP_{3}}(t=0) \\ N_{IP_{4}}+N_{Itk-IP_{4}}+N_{S}=N_{S}(t=0) \end{matrix}$$

ODEs for Model 7: The reactions for Model 7 are given in the Table S7. The corresponding ODEs are the same as Model 1 but the rate constants are different.

**Description of the models:**

Models M1-M4 and M7 contain IP_4_ mediated feedbacks. Models M5-M6 lack those feedbacks. Models M4 and M6 contain monomeric Itk molecules. Models M1-M3, M5 and M7 contain dimeric Itk molecules. In model M1 (Figure S1B), PIP_3_ binding to one of the PH domains increases the affinity of the other PH domain for both IP_4_ and PIP_3_. M2, M3 and M7 (Figure S1B) are variants of this model where PIP_3_ binding to one PH domain in a dimer does not change the affinity of the other PH domain for either IP_4_ or PIP_3_ (M2), or allosterically increases the affinity of the other PH domain for IP_4_ but *not* PIP_3_ (M3), or allosterically increases the affinity of the other PH domain for PIP_3_ but *not* IP_4_ (M7). These models probed potential secondary interactions between Itk dimers and the membrane lipids. In the monomeric model, M4 (Figure S1B), IP_4_ binds the single Itk PH domain with a weak affinity and induces a conformational change (denoted by Itk* in Table S4) that increases the affinity of this PH domain for both PIP_3_ and IP_4_. In the models lacking IP_4_ mediated positive feedbacks, the PH domains of dimeric (M5) or monomeric (M6) Itk molecules bind both PIP_3_ and IP_4_ without an allosteric modification of the binding affinities. The models are summarized in Table I in the main text. We describe the reactions used for each of the models in the Tables S1-S7. In all models we make the following assumptions to make the models simpler while preserving important general features. The initial species numbers are given in Table S8.

**Assumptions (M1-M7).**

1. The initial concentration of the molecular species LAT bound Itk, is referred to as Itk^0^, and, has been used as a surrogate of signal strength given by antigen dose or antigens of different affinities. The stronger the stimulation, the larger is the value of Itk^0^.
2. The details of signal dependent recruitment of PI3K and consequent production of PIP_3_ by PI3K have not been considered. PIP_3_ is produced within seconds after stimulation ([4-6](#_ENREF_4)). Hence, we started our simulations with an initial concentration of PIP_3_, referred to as PIP_3_^0^ from now on. For a given strength of stimulation (i.e. binding of a peptide of a particular affinity), the concentration of total PIP_3_ (bound plus unbound) has been held fixed to the initial PIP_3_ concentration for the entirety of the simulation. The number of PIP_3_^0^ is decreased as the peptide affinities are decreased in order to mimic decreasing PI3K-mediated PIP_3_ production with decreasing intensity of TCR engagement. For example, we have used PIP_3_^0^ = 530 molecules for OVA stimulation but only 50 molecules of PIP_3_^0^ for G4 stimulation.
3. As PLCγ1 is the immediate downstream effector of PIP_3_ bound Itk, we have assumed that the kinetics of the concentration of PIP_3_ bound Itk directly represents PLCγ1 activation kinetics.
4. A 5-phosphatase, responsible for the turnover of IP_4_, is absent in our models. Following TCR ligation, the experimentally determined IP_4_ level in thymocytes/ T cells increases until it reaches its peak at around 10 mins ([7](#_ENREF_7)). Then owing to the 5-phosphatase activity, IP_4_ levels decrease, decaying to half of its peak value at around 30 mins ([7](#_ENREF_7)). Activation of PLCγ1 on the other hand is much faster, peaking at around 1 to 2 mins (main text, ([3](#_ENREF_3))). Based on these data, we believe that the role of 5-phosphatase, if any, in the activation of PLCγ1, is insignificant.

**Unit Conversion Table**

1μM = 600 molecules/(μm)^3^

k_on_^3D^ = 1 (μM^-1^s^-1^) = 0.16 x 10^-2^ (μm)3/ (molecules) s^-1^

Note that the reactions involving two plasma membrane bound complexes take place only at the plasma membrane. Hence we have to convert our k_on_^3D^ to the corresponding k_on_^2D^ in order to describe a binding reaction on a plane. This is done by dividing the k_on_^3D^ by the lengths *l* = 2 nm (Figure S1A).

**Effects of intrinsic noise fluctuations**

The above Figure S2 shows that the stochastic trajectories spread around the solution of the ODEs (shown in red). In the Figure S3 we show how the ODE solution compares with stochastic trajectories when averaged over a small number of *in silico* “cells” for Itk^0^=20 molecules and PIP_3_^0^=50 molecules, where the effect of the stochastic fluctuations is expected to be the largest. The smaller the difference between the two, the more accurate the ODEs are in describing the kinetics for the cell population, even in the presence of intrinsic stochastic fluctuations. We observed that for all the models the ODE solutions produce qualitatively similar shapes as the average stochastic trajectories. Model M7 is not shown. These observations lead us to believe that the intrinsic noise fluctuations are not strong enough to qualitatively change the outcome of our models.

**Understanding mechanisms that regulate of the shape of the temporal profiles of PIP_3_ bound Itk**

We constructed simpler models that effectively describe M1-M4 to analyze the effect of the feedbacks and the binding-unbinding reactions in controlling the shape of the kinetics of PIP_3_ bound Itk. The simpler models could be analytically tractable under certain conditions which allowed us to characterize the dependence of kinetics on the reaction rates and concentrations. Such calculations are usually very useful in gleaning mechanistic understanding into the system.

Effect of the positive feedback: We aimed to understand how the positive feedback controls τ_p_ as the initial concentrations of Itk (Itk^0^) and PIP_3_ (PIP_3_^0^) were varied. In models M1-M4, multiple reactions occurring at different time scales work in concert to create the positive feedback which in essence increases the binding affinity of Itk PH domains for PIP_3_. Therefore, in order to analyze the initial concentration dependence of the peak time, *τ_p_*, we constructed an effective binding unbinding reaction between Itk and PIP_3_, where, the reaction rates (*k_1_* and *k_-1_*) are initial concentration dependent.

Reaction 1

The concentration dependence in the above reaction rates can arise because the effective reaction captures the kinetics of binding of Itk to PIP_3_ in models M1-M4, where multiple second order reactions associated with different time scales induce positive feedback interactions between Itk and PIP_3_. In order to compare reaction 1 with the effect of only the positive feedback, we removed the negative feedback interactions from models M1-M4. Therefore, in all the models, the concentration of Itk bound PIP_3_ reached a non-zero concentration at the steady state (Figure S6A, black curve). Then we estimated the effective rates, *k_1_* and *k_-1_* that will produce similar kinetics (same *τ_1/2_* and the same steady state) (Figure S6B, blue curve) following the scheme below. The kinetics of *x* = Itk – PIP_3_ in Reaction 1 is given by,

(1)

where, Itk^0^ and PIP_3_^0^ denote the initial concentrations of Itk and PIP_3_, respectively, and, K_D_ = *k_-1_/k_1_*. The solution of the above equation is,

(2)

and being the two steady state (one stable another unstable) solutions given by

(3)

The time () taken by *x*, to reach the half of the steady state (the stable fixed point in Eq. (3)) concentration is given by,

. (4)

For a particular set of initial concentrations, we calculated the steady state concentration of PIP_3_ bound Itk, and, τ_1/2_, by numerically solving the corresponding ODEs for models M1-M4 with the negative feedbacks being turned off. Then using Eqns. (3) and (4), we estimated the rate constants, *k_1_* and K_D_ (or, equivalently, *k_1_* and *k_-1_*) for each set of initial concentrations (Figure S7 and S8). Both *k_1_* and K_D_ varied with initial concentrations of Itk and PIP_3_. However, K_D_ did not change appreciably with concentrations for M1 and M3 as compared to M4 or M2 (Figure S7). M1 and M3 showed qualitatively similar variations in K_D_ and *k_1_* with increasing initial concentrations. This demonstrates a large degree of similarity between the models. For models M1-M3, the values of K_D_ in the effective binding-unbinding reaction are substantially smaller (<100 times) to bare Itk and PIP_3_ interaction (K_D_ = 2000) used in Tables S1-S3, in the absence of any IP_4_ feedback. This again demonstrates that the feedback reactions convert the low affinity interactions between Itk and PIP_3_ to a high affinity binding unbinding reaction.

Next we analyzed the concentration dependence of τ_1/2_ in the effective binding-unbinding reaction. This provided us with an estimate of concentration dependence of τ_p_ in model M1-M4, when the negative feedbacks do not contribute appreciably to τ_p_. When, , as in Figure S7, the Eqn (4) can be well approximated by,

(5)

For the range of concentrations of Itk^0^ and PIP_3_^0^ that we have considered in the simulation of our models,

*constant*, therefore,

(6)

When *k_1_* does not depend on concentrations, a tenfold increase in PIP_3_^0^ – Itk^0^, will lead to a tenfold decrease in τ_1/2_. However, our calculations showed that the effective *k_1_* also changes with Itk^0^ and PIP_3_^0^ (Figure S8). E.g., *k_1_* decreased roughly two fold in models M1 and M3 (Figure S8) as Itk^0^ and PIP_3_^0^ were increased, implying that when PIP_3_^0^ – Itk^0^ is increased ten times, the decrease in will be roughly 5 times instead of 10 times. For M2, however, owing to the four times increase in *k_1_*, *τ*_1/2_ will decrease twenty fold for the same increase in PIP_3_^0^ – Itk^0^. This is similar to what we observe in Figure S5 for the variation of τ_p_ with initial PIP_3_ and Itk concentrations. For M1 and M3, the concentration dependence in *k_1_* actually restricts the variation in (~5 times), while in M2 it helps in the variation in (~20 times).

For model M4, K_D_ showed a monotonic increase with increasing Itk^0^ and PIP_3_^0^ (Figure S7). However, values of K_D_ are much smaller than (Itk^0^ + PIP_3_^0^) for the range of concentrations we considered, therefore, we can still use Eq. (6) to estimate τ_1/2_. In contrast, for model M4, *k_1_* did not vary appreciably with Itk^0^ and PIP_3_^0^, therefore, the variation in τ_1/2_ is largely determined by the change in with Itk^0^ and PIP_3_^0^ as given by Eq. (6). This is reflected in the dependence of τ_p_ on initial Itk and PIP_3_ concentrations.

Models (M5 and M6) lacking positive feedbacks:

For models M5 and M6, when the negative feedbacks are turned off, the effective binding unbinding reaction can represent the kinetics of Itk – PIP_3_ with constant K_D_ and *k_1_* for all concentrations. The estimated K_D_ values for the effective reaction were much larger (K_D_~2000) compared to the values of Itk^0^ and PIP_3_^0^ we considered in the simulations, i,e, . Therefore, in this situation can be approximated by

. (7)

where, , is solely determined by the unbinding rate and does not show any concentration dependence. This is in agreement with the results (Figure S5) for models M5-M6.

**Dependence of the decay time τ_d_ of PIP_3_ bound Itk on the initial concentrations of Itk and PIP_3_ due to the negative feedbacks**

The decay time characterizes the time scale for the decay of the concentration of PIP_3_ bound Itk from its peak value as IP_4_ molecules outnumber PIP_3_ molecules. We defined as the difference of the time (t_2_) taken to decay to the half maximum value after the peak value is reached and the peak time, . The dependence of on the initial concentrations of Itk and PIP_3_ manifests in the variations of the asymmetry ratio R with increasing Itk^0^ and PIP_3_^0^ as shown in Fig. 2 in the main text. We aim to characterize the concentration dependence of for the different models in this section.

Feedback models (M1-M3) with Itk dimers:

Owing to the strong positive feedback, most of the PIP_3_ bound Itk molecules exist in PIP_3_ – Itk – Itk – IP_4_ heterodimers. Itk is sequestered into the cytosol via the reactions inducing negative feedbacks in the system as a result of formation of the soluble complex IP_4_ – Itk – Itk – IP_4_. This complex is produced by reactions occurring via two channels:

Channel I

Reaction 2

Channel II

Reaction 3

When the concentration of IP_4_ is much larger than that of PIP_3_^0^, formation of the soluble IP_4_ – Itk – Itk – IP_4_ is more likely to occur through channel I, because, in channel II when IP_4_ unbinds from PIP_3_ – Itk – Itk – IP_4_, the complex, Itk – Itk – PIP_3_ is quickly transformed back to PIP_3_ – Itk – Itk – IP_4_*,* due to the presence of large number of IP_4_ molecules. The rate of change of can be described by (8)

,where, and are high affinity binding unbinding rates of IP_4_ and PIP_3_ to the Itk PH domains. The terms in the first and the second parentheses in the right hand side describe the binding unbinding reactions in channel I and II, respectively. We have considered the rates to be the same for IP_4_ and PIP_3_, therefore, the above reaction is more appropriate for models M1 and M3. However, the general conclusions drawn in this calculation will apply for M2 as well. As argued above, when concentration of IP_4_ is much larger than that of PIP_3_, the first set of binding unbinding reactions in channel II occur at faster time scales, and . In addition, a large number of IP_4_ molecules quickly convert the unstable complexes into stable complexes (reactions in channel I). Therefore, we can write down the following inequality, . Keeping in mind $\widetilde{k_{1}}.[Itk-Itk-IP_{4}][PIP_{3}]\ll\widetilde{k_{1}}.[Itk-Itk-IP_{4}][IP_{4}]$, *x* in Eq. (8) can be approximated as,

(9)

Therefore, in this situation, the decay time does not appreciably depend on the initial concentrations of Itk and PIP_3_, and is determined by the unbinding rate of PIP_3_ from the complex (Figure S9).

The above results change for M2, as IP_4_ binding does not stabilize binding of PIP_3_ to the Itk dimers. Therefore, the reactions in channel II play a greater role in the decay of *x*, and consequently, the decay time scales in M2 vary appreciably with the initial concentrations of Itk and PIP_3_ (Figure S9).

Models lacking feedbacks (M5-M6):

Since both IP_4_ and PIP_3_ bind to Itk PH domains with a low affinity in M5 and M6, a large excess of IP_4_ is required to sequester Itk into the cytosol. We quantify the amount of IP_4_ that is required to effectively sequester Itk into the cytosol below.

Let us consider model M6. The rates of change of and are

given by

(10)

, where, Itk^0^ and PIP_3_^0^ denote the initial concentrations of Itk and PIP_3_, respectively. Using, , where, denotes the initial concentration of PIP_2_, we find that at the steady state,

(11)

*x*_s_ can be calculated from the above equations,

(12)

In order to get a finite τ_w_, the kinetic of Itk-PIP_3_ has to decay to half of its peak value (*A*) i.e., *x*_s_(S^0^) < *A*/2, for a given K_D_, PIP_3_^0^ and Itk^0^. It is however hard to analytically write down a closed form of *A*. Instead, we can use *x*_s_(S^0^=0) as an approximate upper bound of *A* (*A*_max_). The reason being, for S^0^=0 i.e., when PIP_2_ concentration is zero, we recover the steady state for the binding unbinding process in absence of any competition from IP_4_. This will represent the largest value *A* can ever attain. We used this as our approximation for the peak value (*A*) of PIP_3_ recruited Itk in M6. The variation of as a function of for two separate K_D_ is shown in Figure S10.

In Figure S10, the values of *x_s_* at S_0_=0 denote the values for *A*_max_. Figure S10A shows that for high K_D_ values the system requires a large concentration of S^0^ (roughly 3000 molecules) to reach a steady state just about the half of *A*_max_. Moreover, when K_D_ is high, *A*_max_ is small (Figure S10A to Figure S10B), which in turn slows down the production rate of IP_4_. We can estimate the initial concentration dependence of this time scale as follows. The fastest time scale for IP_4_ production is given by , where, *k_cat_* is the rate at which PIP_2_ is converted into IP_4_ by Itk – PIP_3_ by a one step reaction (Tables S1-S6). Since, *A*_max_ grows in a graded fashion with the increase in Itk^0^ and PIP_3_^0^ (Eqn 12), the production timescales for IP_4_ generation decrease slowly with increasing Itk^0^ and PIP_3_^0^. Both these facts play hand in hand to give rise to a larger τ_d_ in M5 and M6 as compared to models M1-M3.

Model M4:

In model M4, in addition to the low affinity binding unbinding reactions, there is a bi-directional high affinity augmentation process. Following the same procedure as described above we compute the steady state concentration of PIP_3_ bound Itk for M4. Denoting concentrations of , and *Itk-IP_4_* by *x*_1_, *x*_2_ and *y*, respectively, the rate equations are given by,

(13)

where, and are the usual binding unbinding rates while is the high affinity augmentation rate. is the sum of and . In the steady state we have,

(14)

The subscript “s” is used denote steady state concentrations. From Eqn (14) it is apparent that

(15)

which implies *x_1s_*=*x_2s_* for nonzero values of *y_s_*. Making use of this fact we have,

. (16)

Eqn(16) is a cubic equation yielding three real roots for the values of K_D_, PIP_3_^0^ and Itk^0^ we have used. However, only one root provides physically meaningful result, the other roots create an unphysical situation where *x_s_* > Itk^0^. We show the variation of *x_s_* with two different values of K_D_ below (Figure S11). Following similar analysis as in Figure S10, we find that a large number of substrate is required to bring down the activation of PIP_3_ bound Itk. *A*_max_ in this case was calculated by taking a limit , note, *x_s_* has a discontinuity at S^0^=0.

**Variation of R with increasing initial concentrations of Itk and PIP3 for models M1-M6**

The asymmetry ratio, R, is calculated using, , where, τ_w_ is the width of the temporal profile of the concentration of PIP_3_ bound Itk. τ_w_ can be expressed in terms of the decay time, τ_d_, t_1_ (the time system takes to reach half of the peak concentration) and the peak time τ_p_. We aimed to understand the variation of R with increasing Itk^0^ and PIP_3_^0^ (Fig. 2) based on the results described in the last two sections.

Models M1 and M3

The asymmetry ratio, R, increases as Itk^0^ and PIP_3_^0^ are increased. Our calculations show (Figure S9) that τ_w_ does not vary appreciably as Itk^0^ and PIP_3_^0^ are increased in the range of moderate to high values. This occurs because the decay time, τ_d_, is primarily determined by the small unbinding rate of PIP_3_ from the stable IP_4_ – Itk – Itk–PIP_3_ complex. Since τ_d_ is much larger than the peak time in this range, τ_w_ is mainly determined by τ_d_ in this range of concentrations. The increase in R, hence, arises from the decrease in τ_p_ as Itk^0^ and PIP_3_^0^ are increased. The concentration dependence of this variation is determined by Eq. (5). At lower concentrations of Itk^0^ and PIP_3_^0^, both τ_w_ and τ_d_ decrease, as Itk^0^ and PIP_3_^0^ are decreased, however, τ_p_ decreases at a faster rate compared to τ_d_, resulting in increasing R values.

Model M2

In this model, in contrast to models M1 and M3, the asymmetry ratio, R, initially decreases with increasing Itk^0^ and PIP_3_^0^, and, then at larger values of Itk^0^ and PIP_3_^0^ starts increasing with increasing Itk^0^ and PIP_3_^0^. At smaller values of Itk^0^ and PIP_3_^0^, τ_w_, decreases (Figure S9A) at a much faster rate as compared to τ_p_, as Itk^0^ and PIP_3_^0^ are increased. At higher values of Itk^0^ and PIP_3_^0^, τ_w_ does not change appreciably with increasing initial concentrations because of the same mechanisms as described for M1 and M3. However, in this range of concentrations τ_p_ decreases with increasing Itk^0^ and PIP_3_^0^ resulting in increased R as the initial concentrations increase.

Models M5 and M6

For these models, τ_d_ is substantially larger than τ_p_, therefore, τ_w_ is well approximated by τ_d_. Since τ_p_ does not change appreciably, but τ_d_ decreases with increasing Itk^0^ and PIP_3_^0^ we see a decrease in the ratio R as the initial concentrations are increased.

Model M4

τ_w_ behaves in a very similar manner to models M5 and M6. τ_p_ for M4 decreases with increasing Itk^0^ and PIP_3_^0^ following Eqn (5), however, the rate of the decrease is still smaller than that of τ_w_, which results in decrease in the values of R as the initial concentrations are increased.

Model 7 can be analyzed in a similar way.

**Relative Entropy calculation and MaxEnt Analysis**

The continuous relative entropy is defined as

$\text{D}_{\text{KL}}=\int_{x\in K} p(x)\log\frac{p(x)}{q(x)}dx$ (17)

where, is the distribution of the parameters denoted by *x* ∈*K*, which is subject to constraints imposed by experiments, and, is a uniform distribution, such that for all . Now we seek for which maximizes Eqn (17) under the constraints:

$$\int_{x\in K} A(x)p(x)dx=A_{avg}=A_{\exp}$$

(18)

We use three Lagrange multipliers (λ, λ_1_, λ_2_ and λ_3_) to incorporate the constraints in Eq. (18) and maximize the following function:

$$\begin{matrix} G(p,\lambda,\lambda_{1},\lambda_{2},\lambda_{3})=\int_{x\in K} p(x)\log\frac{p(x)}{q(x)}dx+\lambda\left(\int_{x\in K} p(x)-1\right)+\lambda_{1}\left(\int_{x\in K} R(x)p(x)dx-R_{avg}\right)+\lambda_{2}\left(\int_{x\in K} \tau(x)p(x)dx-\tau_{avg}\right) \\ \text{ +}\lambda_{3}\left(\int_{x\in K} A(x)p(x)dx-A_{avg}\right) \\ \frac{\partial G}{\partial p}=0\Rightarrow\log\frac{p(x)}{q(x)}+1+\lambda+\lambda_{1}R(x)+\lambda_{2}\tau(x)+\lambda_{3}A(x)=0 \\ \frac{\partial G}{\partial\lambda}=0\Rightarrow\text{ }\int_{x\in K} p(x)dx=1 \\ \frac{\partial G}{\partial\lambda_{1}}=0\Rightarrow\int_{x\in K} R(x)p(x)dx=R_{avg} \\ \frac{\partial G}{\partial\lambda_{2}}=0\Rightarrow\int_{x\in K} \tau(x)p(x)dx=\tau_{avg} \\ \frac{\partial G}{\partial\lambda_{3}}=0\Rightarrow\int_{x\in K} A(x)p(x)dx=A_{avg} \end{matrix}$$

(19)

From Eqn (19) it is clear that

$$p(x)=\frac{q(x)e^{-\lambda_{1}R(x)-\lambda_{2}\tau(x)-\lambda_{3}A(x)}}{\int_{x\in k} q(x)e^{-\lambda_{1}R(x)-\lambda_{2}\tau(x)-\lambda_{3}A(x)}}$$

$$R_{avg}=\frac{\int_{x\in k} R(x)q(x)e^{-\lambda_{1}R(x)-\lambda_{2}\tau(x)-\lambda_{3}A(x)}}{\int_{x\in k} q(x)e^{-\lambda_{1}R(x)-\lambda_{2}\tau(x)-\lambda_{3}A(x)}}$$

$$\tau_{avg}=\frac{\int_{x\in K} \tau(x)q(x)e^{-\lambda_{1}R(x)-\lambda_{2}\tau(x)-\lambda_{3}A(x)}}{\int_{x\in k} q(x)e^{-\lambda_{1}R(x)-\lambda_{2}\tau(x)-\lambda_{3}A(x)}}$$

$$A_{avg}=\frac{\int_{x\in K} A(x)q(x)e^{-\lambda_{1}R(x)-\lambda_{2}\tau(x)-\lambda_{3}A(x)}}{\int_{x\in k} q(x)e^{-\lambda_{1}R(x)-\lambda_{2}\tau(x)-\lambda_{3}A(x)}}$$

We substituted the p(x) from Eq. (19) in the equations for , and *A_avg_* (Eq. 18), and solved for , and λ_3_ when the values of R_avg_ and τ_avg_ were taken from the table II in the main text. Owing to the lack of knowledge about the absolute value of *A_avg_*, we have used some *A_avg_*, which all the models can yield. (We have also varied the *A_avg_*, to study how the dependence of the robustness on the choice of *A_avg_*). Then we calculate the Kullback-Leibler distance

(20)

for each model.

*Range of parameter variation:* For the results shown in Figures S12, 13, 14, 15,16, 17, 19, 20, 21, 22 and 23, the rate constants were chosen from uniform distributions with lower and upper bounds equal to 1/10 and 10 times, respectively, the base values shown in Tables S1-S7. We used 100,000 sample points, each point representing a set of rate constants and initial concentrations for all the models. For models M1-M3 the high affinity binding unbinding rates are drawn from a uniform distribution whilst the low affinity K_D_ is determined as K_D_^low^= α K_D_^high^, where α is drawn from a uniform distribution with lower and upper bound of 1 and 4000 respectively. For M7, while high affinity binding and unbinding rates are drawn from a uniform distribution, α is chosen uniformly from 1 to 50. The initial concentrations of Itk and PIP_3_ were varied within a 35% ([11](#_ENREF_11)) range from uniform distributions centered at the base values shown in Table S8.

**The effect of Lck mediated phosphorylation of PIP_3_ bound Itk complex.**

We have studied the effect of Lck mediated phosphorylation of membrane bound Itk at itsY511 residue. Here the membrane bound Itk is phosphorylated by Lck (modeled as a first order reaction). Upon activation it becomes active (denoted by *Itk^act^* except for M4 where it is denoted as *Itk_act_*). Only an active form of Itk is capable of producing IP_4_ from the hydrolysis of PIP_2_. The activation and de-activation rates of membrane bound Itk is chosen so that the kinetics of active Itk roughly agrees with the experimental data in the main text and ([3](#_ENREF_3)). The reactions and the reaction rates are detailed in Table S9-S15. We find that adding the Lck mediated phosphorylation of the membrane bound Itk does not alter the rank order of the models.

**Dependence of D_KL_ on parameters those weakly influence the Itk-PIP_3_ kinetics.**

Let us assume that the peak time τ_p_, the amplitude *A* and the ratio R depend on n parameters. If we add m new parameters, which do not in any way influence the outcome of the Itk-PIP_3_ kinetics then the joint probability distribution that maximizes the entropy with the constraints becomes

$$p\left(k_{1},...,k_{n+m}\right)=p\left(k_{1},...,k_{n}\right)p(k_{n+1},....,k_{n+m})$$

Now as *k_n+1_,....,k_n+m_* are drawn from a uniform distribution and they do not contribute anything to the observables, p(*k_n+1_,....,k_n+m_*) = *q*(*k_n+1_,....,k_n+m_*), where *q* is a uniform distribution. Therefore

$$\text{D}_{KL}^{new}=\int d^{n+m}k\text{ }p\left(k_{1},...,k_{n+m}\right)\ln\frac{p\left(k_{1},...,k_{n+m}\right)}{q\left(k_{1},...,k_{n+m}\right)}=\int d^{n}k\text{ }p\left(k_{1},...,k_{n}\right)\ln\frac{p\left(k_{1},...,k_{n}\right)}{q\left(k_{1},...,k_{n}\right)}\int d^{m}k\text{ }p(k_{n+1},....,k_{n+m})$$

In order to probe the effect of parameters those weakly influence the kinetics of Itk-PIP_3_ we have carried out a simulation for M3 with three added reactions. Instead of approximating the production of IP_4_ from PIP_2_ by a simple one step reaction, we have incorporated the fact that the membrane bound Itk phosphorylates PLCγ which in turn hydrolyses PIP_2_ to form membrane bound DAG and soluble IP_3_. IP_3_ then gets converted into IP_4_. In an effort to render the newly added parameters weak, we have chosen the rate constants in such a way that the PLCγ kinetics follow the Itk-PIP_3_ kinetics and the turnover of IP_3_ to IP_4_ happens very quickly. The variations of these new parameters are confined within a two folds range. The details of the reactions are given in Table S17. From Figure S21 we find that the D_KL_^new^ and D_KL_^Old^ are very similar, differing only in the second place of decimal.

1. Altan-Bonnet G, Germain RN. 2005. Modeling T cell antigen discrimination based on feedback control of digital ERK responses. *PLoS biology* 3: e356

2. Kavran JM, Klein DE, Lee A, Falasca M, Isakoff SJ, Skolnik EY, Lemmon MA. 1998. Specificity and promiscuity in phosphoinositide binding by pleckstrin homology domains. *The Journal of biological chemistry* 273: 30497-508

3. Huang YH, Grasis JA, Miller AT, Xu R, Soonthornvacharin S, Andreotti AH, Tsoukas CD, Cooke MP, Sauer K. 2007. Positive regulation of Itk PH domain function by soluble IP4. *Science* 316: 886-9

4. Costello PS, Gallagher M, Cantrell DA. 2002. Sustained and dynamic inositol lipid metabolism inside and outside the immunological synapse. *Nature immunology* 3: 1082-9

5. Insall RH, Weiner OD. 2001. PIP3, PIP2, and cell movement--similar messages, different meanings? *Developmental cell* 1: 743-7

6. Stephens LR, Jackson TR, Hawkins PT. 1993. Agonist-stimulated synthesis of phosphatidylinositol(3,4,5)-trisphosphate: a new intracellular signalling system? *Biochimica et biophysica acta* 1179: 27-75

7. Guse AH, Emmrich F. 1991. T-cell receptor-mediated metabolism of inositol polyphosphates in Jurkat T-lymphocytes. Identification of a D-myo-inositol 1,2,3,4,6-pentakisphosphate-2-phosphomonoesterase activity, a D-myo-inositol 1,3,4,5,6-pentakisphosphate-1/3-phosphatase activity and a D/L-myo-inositol 1,2,4,5,6-pentakisphosphate-1/3-kinase activity. *The Journal of biological chemistry* 266: 24498-502

8. Rebecchi MJ, Scarlata S. 1998. Pleckstrin homology domains: a common fold with diverse functions. *Annual review of biophysics and biomolecular structure* 27: 503-28

9. Ashok Prasad JZ, Jayajit Das, Jeroen P. Roose, Arthur Weiss and Arup Chakraborty. 2009. Origin of the sharp boundary that discriminates positive and negative selection of thymocytes. *PNAS* 106, no. 2: 528-33

10. Gillespie DT. 1977. Exact Stochastic Simulation of Coupled Chemical-Reactions. *Journal of Physical Chemistry* 81: 2340-61

11. Volfson D, Marciniak J, Blake WJ, Ostroff N, Tsimring LS, Hasty J. 2006. Origins of extrinsic variability in eukaryotic gene expression. *Nature* 439: 861-4
